# Impact of switching from a quadrivalent to a nonavalent HPV vaccine on HPV infections and cervical cancer in Colombia: a mathematical modelling study

**DOI:** 10.1016/j.lana.2026.101483

**Published:** 2026-05-05

**Authors:** Romina Tejada, Armando Baena, Lina Trujillo, Juliana Rodriguez, Mathieu Maheu-Giroux, Maribel Almonte, Eduardo L. Franco, Talía Malagón

**Affiliations:** a Division of Cancer Epidemiology, Gerald Bronfman Department of Oncology, McGill University, Montreal, Canada; b Division of Cancer Epidemiology and Genetics, National Cancer Institute, National Institutes of Health, Rockville, MD, USA; c Department of Gynecologic Oncology, Instituto Nacional de Cancerologia, Bogotá, Colombia; d Department of Gynecology, Obstetrics and Reproductive Medicine, Fundación Santa Fe de Bogotá, Bogotá, Colombia; e Department of Obstetrics and Gynecology, Universidad Nacional de Colombia, Bogotá, Colombia; f Department of Epidemiology, Biostatistics, and Occupational Health, School of Population and Global Health, McGill University, Montréal, Canada; g Department of Noncommunicable Diseases and Mental Health, World Health Organization, Geneva, Switzerland; h St Mary's Research Centre, Montreal West Island Integrated University Health and Social Services Centre, Montréal, Canada

**Keywords:** Papillomavirus infections, Models, Theoretical, Papillomavirus vaccines, Vaccination coverage

## Abstract

**Background:**

In 2022, Colombia reported 13.7 cases of cervical cancer per 100,000 females. Given the persistently low coverage of human papillomavirus (HPV) vaccination among girls (51%), alternative interventions are being considered. We assessed the population-level impact of switching from a quadrivalent to a nonavalent vaccine, as well as increasing vaccination coverage to the World Health Organization's target of 90% (≥1 dose).

**Methods:**

We developed a dynamic model of carcinogenic HPV transmission and vaccination in the population of Colombia aged 15+ years, stratified by health state, sex, age, sexual activity, and HPV vaccination status, accounting for latency. The model was calibrated to HPV prevalence data from Colombia and all Latin America. We evaluated gender-neutral and girl-only routine one-dose vaccination (<15 years) under current Colombian coverage levels and 90% coverage. We estimated age-standardised HPV prevalence, and the relative reduction in HPV prevalence and cervical cancer incidence over 2013–2100.

**Findings:**

Both vaccines reduced age-standardised HPV prevalence, with greater reductions observed at 90% coverage, under a gender-neutral scenario, and with a nonavalent vaccine. Switching to a nonavalent vaccine at current levels could reduce HPV prevalence by 39% (range: 33%–46%) in females by 2100, compared to 8% (range: 1%–17%) when only increasing quadrivalent vaccine coverage to 90% in a gender-neutral vaccination strategy. Only a nonavalent vaccine strategy reduced projected age-standardised cervical cancer incidence rates to below 4 cases per 100,000 females as early as 2058.

**Interpretation:**

Switching to a nonavalent vaccine will accelerate the reduction of HPV infections, thereby expediting progress toward cervical cancer elimination.

**Funding:**

Fonds de recherche du Québec—Santé and the Division of Cancer Epidemiology at McGill University.

**Research in context**

**Evidence before this study**

We searched PubMed (MEDLINE) for studies evaluating human papillomavirus (HPV) vaccination using mathematical or economic models in Colombia, from database inception to April 08, 2026, with no language restrictions. The search combined terms related to HPV vaccination and modelling, including “Papillomavirus Vaccines” [MeSH], “HPV”, “vaccin∗”, “model∗”, “transmission”, and “cost-effectiveness”, along with “Colombia”. We identified a couple of modelling studies assessing the impact and cost-effectiveness of HPV vaccination strategies in Colombia. However, to our knowledge, no previous study has specifically evaluated the impact of switching from the bivalent or quadrivalent to the nonavalent HPV vaccine in this setting.

**Added value of this study**

Previous mathematical models assessing a nonavalent HPV vaccine in low- and middle-income countries have focused on high vaccination coverages in girls-only schedules. Moreover, unlike other studies that use a generic model for low- and middle-income countries, our model specifically incorporates input parameters from the region, providing more accurate and locally relevant results. Finally, a key novelty of our study is the inclusion of latency and reactivation in the natural history of HPV, which avoids implausibly overly optimistic projections and earlier timelines for cervical cancer elimination.

**Implications of all the available evidence**

Our model predicts that achieving the WHO cervical cancer elimination target in Colombia, assuming no modifications to the screening program, requires switching to the nonavalent vaccine. To further accelerate the decline in cervical cancer incidence, it would also be necessary to increase HPV vaccination coverage to 90% in both boys and girls.

## Introduction

Human papillomavirus (HPV) is one of the most common sexually transmitted infections worldwide. It is associated with several anogenital and oropharyngeal cancers, as well as anogenital warts and recurrent respiratory papillomatosis.^1^^,^^2^ Infection with oncogenic HPV is a necessary cause of cervical cancer.^1^ In 2022, the global age-standardised incidence of cervical cancer was 14.1 cases per 100,000 females, with Latin America and the Caribbean accounting for 9.8% of these new cases.^3^ That same year, Colombia reported an age-standardised incidence of 13.7 cervical cancer cases per 100,000 females of all ages. Although this is below the global average, it is still much higher than the World Health Organization (WHO) cervical cancer elimination threshold of 4 per 100,000 females.^4^^,^^5^ Despite the decline in cervical cancer incidence over recent decades due to increased access to health care, screening services, and enhanced living standards, the disease burden remains high, particularly in low- and middle-income countries such as Colombia.^6^

The combination of high-coverage HPV vaccination, effective screening, and timely treatment of precancerous lesions can drastically reduce cervical cancer incidence and mortality.^5^ Unfortunately, challenges in vaccine implementation persist worldwide. Currently, Colombia has one of the lowest HPV vaccination coverages in South America.^7^^,^^8^ These challenges, driven by logistical, cultural, political, and financial barriers, have contributed to suboptimal population-level impact of HPV vaccination programs in eligible girls.^9^^,^^10^ Since 2012, Colombia has offered free, school-based three-dose HPV vaccination with a quadrivalent vaccine to nine-year-old girls. A two-dose schedule was introduced in 2017. In 2023, the program shifted to a single-dose regimen for girls aged 9–17 years and extended eligibility to nine-year-old boys. In 2024, vaccination eligibility was further expanded to include boys aged 9–17.^8^^,^^11^^,^^12^ The HPV vaccination program coverage was 97.5% in 2013, but plummeted to 20.4% in 2014 following rumours that the vaccine had caused illness (i.e. fainting) in girls in El Carmen de Bolِívar. Coverage has not returned to 2013 levels despite a concerted effort.^7^ Although several local and national campaigns attempted to raise awareness about cervical cancer,^13^ vaccination coverage remains low. As of 2023, only 51% of girls and 16% of boys aged 9–15 years had been vaccinated.^14^

Bivalent HPV vaccines protect against HPV 16 and 18, genotypes that are responsible for approximately 70% of cervical cancers.^15^ A quadrivalent HPV vaccine, currently used in Colombia, adds protection against HPV types 6 and 11, which cause anogenital warts and respiratory papillomatosis.^16^ A nonavalent vaccine provides additional protection against HPV types 31, 33, 45, 52, and 58, thus increasing protection against about 90% of cervical cancers.^17^ Several studies have assessed the health and economic impacts of switching from the bivalent or quadrivalent vaccine to a nonavalent vaccine.^18^, ^19^, ^20^, ^21^, ^22^, ^23^, ^24^ They suggest that this transition could prevent a higher number of additional HPV infections, related cancer cases, and deaths in both females and males. However, most have been conducted in high-income countries and assume high vaccination coverage levels achieved rapidly, which may not reflect the situation in low- and middle-income countries (LMICs). Moreover, there are also differences in demographic characteristics, sexual behaviours, and clinical practices related to screening and treatment of cervical cancer that prevent the transferability of the results.

Despite the availability of HPV vaccination and cervical cancer screening in Colombia, cervical cancer remains an important public health concern.^8^ Therefore, new strategies, such as switching to a nonavalent vaccine, are currently being considered by policymakers. These decisions are increasingly guided by health-decision models, which integrate data from multiple sources to estimate long-term population-level outcomes, including indirect benefits of vaccination to those not immunised, and evaluate scenarios that are difficult to directly evaluate in clinical trials.^25^ This evidence is instrumental to inform cervical cancer prevention strategies across low- and middle-income Latin American countries with similar disease dynamics and constrained health resources, using the findings from Colombia presented in this manuscript as a valuable case study for deriving broader regional insights.

In this context, we aimed to evaluate the potential population-level impact of introducing a nonavalent vaccine into Colombia's current vaccination program. Our mathematical model analysis considers both the current strategy of vaccinating boys and girls and the previously implemented female-only schedule, as well as the impact of achieving the WHO's target of 90% HPV vaccination coverage.

## Methods

### Model description

**Fig. 1:**
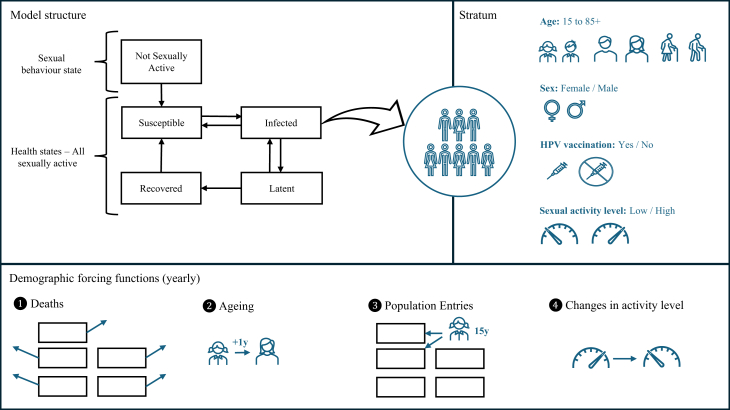
**Conceptual diagram of dynamic transmission model for HPV infection**. The top left panel illustrates the model structure representing the natural history of HPV transmission. Individuals transition between states, with the population in each state stratified according to the characteristics shown in top right panel: age, sex, HPV vaccination status, and sexual activity level. The bottom panel shows the demographic forcing functions applied annually to update the population based on background mortality, ageing (individuals age by one year, except for the open-ended 85+ age group), entry of 15-years-old individuals, and changes in sexual activity level according to age.

We developed a compartmental model to simulate HPV transmission dynamics and vaccination calibrated to the epidemiological context of Colombia. The model builds upon a previously developed ordinary differential equations HPV transmission dynamic model,^26^ which has been substantially expanded for this analysis to include population demography and a more complex natural history of HPV infection (See Supplemental Section Model Description). The system of equations was numerically solved using the “deSolve” R package.^27^ The model simulates an open population of individuals aged 15–85+ years, stratified by health state, sex, age, sexual activity level, and HPV vaccination status (Fig. 1 and Supplemental Figure S1). The term “sex” refers to sex assigned at birth. A forcing event function was used to update demographic parameters at the end of each calendar year.^28^ Data and projections of the age and sex structure of Colombia's population, as well as death rates by age and sex, were obtained from the World Population Prospects (medium fertility variant projections)^29^ (Supplemental Tables S4 and S5, Supplemental Figure S2). Methodology details are available in the supplemental material.

We independently modelled HPV transmission dynamics for three groups of carcinogenic HPV types: (1) HPV 16 and 18, (2) HPV 31, 33, 45, 52, and 58, and (3) other carcinogenic HPV types. The group formation was done considering HPV types included in a quadrivalent and a nonavalent HPV vaccine. The transmission dynamics for each group were considered separately, with no interactions between groups. The model simulates type-specific HPV transmission considering two sexual activity levels: low (≤2 opposite-sex partners/year) and high (>2 partners/year). We modelled instantaneous heterosexual partnership formation using previously established equations and methods to balance sexual mixing matrices.^30^^,^^31^ Partnerships were formed based on patterns of assortative sexual mixing by age group and sexual activity (Supplemental Table S6). Infection occurs when a susceptible individual forms a partnership with an infected person, with a per-partnership probability of infection. Following HPV infection, individuals can either clear the infection and become susceptible again, develop same-type natural immunity, or progress to a latent infection; latent infections may reactivate later. We defined latency as a period of non-detectability, where HPV is present in the epithelium but with low to no viral gene expression and no evidence of disease, hence undetectable.^32^ We assumed that when HPV is latent and undetectable it is untransmissible, as evidence shows extremely low incidence of new HPV infections in partnerships when a person's sex partner is HPV DNA negative.^33^ Both natural and vaccine-induced immunity provide protection against specific HPV types; however, natural immunity wanes over time and is lower than vaccine-induced immunity, which we assume to be lifelong.^34^^,^^35^ Finally, HPV vaccines are prophylactic, reducing HPV transmission by lowering the per-partnership risk of HPV acquisition in vaccinated individuals. In our model, we did not consider any effect of the vaccine on clearing existing infections. Vaccination status is implemented as a model stratification. Vaccine protection is modelled as a relative risk reduction of the probability of infection. The model capture both herd effects and breakthrough infections.

### Model parameters

Model parameters included demographic, sexual behaviour, and HPV natural history variables (Table 1 and Supplemental Table S6). Sexual behaviour parameters were derived from data on the Hispanic population in the National Survey on Family Growth^45^ conducted in the United States of America, as we were unable to find data specific to Colombia. When available, we used HPV natural history parameters stratified by sex and groups of HPV type. See the Supplement for more details on model parameters.

**Table 1:** Model parameters.

Parameter	HPV type	Sex	Prior values^a^	Posterior values^b^	Data source
***Natural history of HPV infection***					
Probability of infection transmission per partnership with an infected person	16/18	Both	0.87 (0.60–0.99)	0.62 (0.25–0.93)	Model based (POSCBOCAN)^36^
	31/33/45/52/58		0.79 (0.30–0.99)	0.77 (0.25–0.99)	
	Non-vaccine		0.87 (0.38–0.99)	0.84 (0.50–0.99)	
Duration of infection (months)	16/18	Female	18 (14–22)	18 (12–22)	Placebo arm of FUTURE study^37^
		Male	10 (6–20)	9 (5–16)	HIM study^38^
	31/33/45/52/58	Female	18 (13–23)	19 (15–26)	Placebo arm of FUTURE study^37^
		Male	7 (6–18)	8 (5–16)	HIM study^38^
	Non-vaccine	Female	13 (12–16)	13 (11–15)	Placebo arm of FUTURE study^37^
		Male	8 (6–18)	9 (5–14)	HIM study^38^
Proportion of infections conferring natural immunity	16/18	Female	0.568 (0.348–0.754)	0.58 (0.39–0.72)	University student Washington (1990–1998)^39^
		Male	0.05 (0.004–0.13)	0.06 (0.01–0.18)	Baseline from an RCT on HPV vaccine in males^40^
	31/33/45/52/58	Female	0.33 (0.27–0.35)	0.33 (0.28–0.36)	Secondary analysis from HPV vaccine trial^41^
		Male	0.07 (0.04–0.11)	0.07 (0.04–0.09)	Baseline from an RCT on HPV vaccine in males^40^
	Non-vaccine	Female	0.35 (0.19–0.55)	0.25 (0.15–0.41)	Cervical cancer screening program in Slovenia^42^
		Male	0.13 (0.02–0.61)	0.13 (0.02–0.40)	Young male seroconversion for HPV35^43^
Duration of immunity (years)	16/18	Both	0.5–12	7.13 (0.96–11.83)	Assumed
	31/33/45/52/58			4.57 (0.52–12.00)	
	Non-vaccine			7.69 (1.16–11.92)	
Proportion of HPV infections becoming latent	16/18	Both	0.5 (0.1–0.9)	0.87 (0.67–0.99)	Assumed
	31/33/45/52/58			0.40 (0.07–0.81)	
	Non-vaccine			0.03 (0.001–0.10)	
Rate of reactivations of latent HPV infections (per year)	16/18	Both	0.17 (0.11–0.24)	0.15 (0.11–0.22)	Ludwig-McGill cohort^44^
	31/33/45/52/58			0.17 (0.12–0.25)	
	Non-vaccine			0.16 (0.11–0.25)	
***Sexual behaviour and infection transmission***					
Proportion of sexual partnerships made exclusively with same sexual activity level	16/18	Both	0.2 (0.004–0.779)	0.23 (0.06–0.63)	Calibrated
	31/33/45/52/58			0.22 (0.07–0.63)	
	Non-vaccine			0.16 (0.06–0.57)	
Proportion of sexual partnerships made exclusively with same age group	16/18	Female	0.41 (0.1–0.84)	0.33 (0.05–0.68)	National Survey of Family Growth (2017–2019)^45^
		Male	0.48 (0.1–0.99)	0.27 (0.02–0.76)	
	31/33/45/52/58	Female	0.41 (0.1–0.84)	0.33 (0.05–0.47)	
		Male	0.48 (0.1–0.99)	0.09 (0.02–0.56)	
	Non-vaccine	Female	0.41 (0.1–0.84)	0.33 (0.08–0.76)	
		Male	0.48 (0.1–0.99)	0.50 (0.13–0.68)	
Compromise between female and male's desires for sexual partnerships^c^	All	Both	0.5		Assumed

HPV, human papillomavirus; RCT, randomized controlled trial.

Note: Minor differences in posterior sexual behaviour parameter values across HPV type groups reflect the calibration and resampling procedure rather than true behavioural differences, as the same underlying parameter sets were used for all groups.

a Mean (95% Confidence interval).

b Mean (range) of 50 best fitting parameter sets.

c A value of 1 indicates that the woman makes the choice, while a value of 0 indicates that the man makes the choice. Any value between 0 and 1 represents a compromise between both preferences.

### Calibration

We calibrated sexual behaviour parameters (assortativity by age and sexual activity level, and new partner acquisition rate), as well as natural history parameters (e.g., latency rate) specific to each group of HPV types (Supplemental Table S7). Using Latin Hypercube Sampling, we generated 10,000 parameter sets and selected the best 50 sets, for each group of HPV types, through a hybrid approach. First, we retained parameter sets that projected HPV prevalences within the confidence intervals reported in studies of age-stratified carcinogenic HPV prevalence in Colombia^46^, ^47^, ^48^, ^49^, ^50^, ^51^, ^52^, ^53^ and Latin America.^54^^,^^55^ Next, we selected the 50 sets with the highest log-likelihood, using this as a measure of goodness-of-fit, to assess the agreement between estimated and observed age-stratified carcinogenic HPV prevalence data for each group of HPV types in both female and male populations. For the calibration process, we assumed the population was unvaccinated against HPV, as the HPV prevalence data in the literature correspond to unvaccinated populations that were not eligible for vaccination due to age at the time of program implementation. Further details on the calibration process are provided in the Supplement.

### Vaccination scenarios

We evaluated routine vaccination of girls as well as gender-neutral vaccination with one dose before the age of 15. While in our model vaccine policies and parameters are defined by sex rather than gender, the historical convention has been to refer to HPV vaccination programs targeting both sexes as “gender-neutral vaccination”. Therefore, we use this term throughout the manuscript.

The base case scenario assumed the introduction of the quadrivalent vaccine in 2013, with an alternative scenario in which the program switched to the nonavalent vaccine in 2026. We also considered three vaccination coverage scenarios. In all scenarios, vaccination was assumed to begin in 2013 for girls at 51% coverage, and in 2024 for boys at 16% coverage.^14^ (i) Current coverage: 51% for girls and 16% for boys maintained over time; (ii) Equal coverage: starting in 2026, boys reach the same 51% coverage as girls; and (iii) WHO target: starting in 2026, coverage increases to 90% in girls for the female-only scenario, and to 90% in both girls and boys for the gender-neutral scenario.^5^ Vaccine efficacy was assumed to be 98.0% in girls and 87.4% in boys against HPVs 16 and 18 for both a quadrivalent and nonavalent HPV vaccines,^56^^,^^57^ and 96.9% in girls and 93.8% in boys against HPV 31, 33, 45, 52 and 58 for a nonavalent HPV vaccine.^18^^,^^58^

### Outcomes

The analysis covered a time horizon from 2013 to 2100. We present results for the year 2030, aligning with the WHO's target year for achieving the 90% vaccination target, for 2040 to assess progress a decade beyond this target, and for 2100 as the endpoint of the simulation. We calculated age-standardised carcinogenic HPV infection incidence and prevalence over time for each group of HPV types and vaccination and coverage scenarios. We age-standardised HPV infection outputs to the 2010 Colombian population age-structure as reported by the United Nations to allow comparison across scenarios.^29^ We also report absolute case counts based on the modelled population. Results for the three groups of HPV types were combined to estimate reductions in overall carcinogenic HPV prevalence and incidence using co-infection distributions derived from the ESTAMPA study.^53^ The ESTAMPA study is a multicenter study in Latin America that aims to evaluate the performance of different triage techniques to detect cervical pre-malignant lesions.^54^ We calculated age-standardised carcinogenic HPV prevalence by vaccination status to assess the frequency of breakthrough infections in the vaccinated individuals and herd effects to unvaccinated individuals. To account for uncertainty, we report the median and minimum–maximum range of results based on the 50 highest–likelihood parameter sets retained after calibration. These sets represent the best-fitting parameterisations rather than a random sample from the parameter space.

### Projections on cervical cancer cases

To assess if both quadrivalent and nonavalent HPV vaccines could achieve the WHO cervical cancer elimination goal of 4 cases per 100,000 females,^5^ assuming no changes in cervical cancer screening practices, we projected the age-standardised cervical cancer incidence resulting from the weighted age-specific relative reduction in carcinogenic HPV prevalence based on HPV type distributions in cervical cancer. Cervical cancer projections were not generated through explicit modelling of disease progression but were estimated by applying HPV type–specific weights. We used the reported type distribution for Colombia (Baena A, personal communication), where 74% of cervical cancers are associated with HPV 16 or HPV 18, and 24% with one of HPV 31, 33, 45, 52, or 58, and the remaining to other non-vaccinal HPV types which we assumed would not be impacted by vaccination. We age-standardised projected cervical cancer incidence rates to the 2015 World Female Population^29^ (a sensitivity analysis using the 1960 Segi/Doll world population for standardisation was also conducted).^59^ We then identified the year at which carcinogenic HPV prevalence will be sufficiently reduced for cervical cancer incidence to be below the elimination threshold of 4 per 100,000 females. Cervical cancer incidence projections were age-standardised using a world standard population (2015 World Female Population and 1960 Segi/Doll world population) to ensure comparability with international studies and the cervical cancer elimination threshold, whereas HPV prevalence and incidence were age-standardised to the local population age structure relevant for national interpretation.

We assumed that the 2022 cervical cancer incidence in Colombia^60^ represents the expected cancer incidence without vaccination; since vaccination started in 2013, the female population vaccinated against HPV is currently too young to have developed cervical cancer.^12^ Currently, cervical cancer screening in Colombia is conducted with cytology with a 1-1-3 years schedule for females between 25 and 29 years old and with a HPV-DNA test every five years between 30 and 65 years old.^61^ Screening coverage for the last three years in females aged 25–65 years old was 68% (62%–73%) in 2019.^62^

### Ethical statement

The present study consists in a mathematical model of HPV transmission, informed by secondary data sources, including published literature and publicly available datasets. No individual-level or identifiable personal data were used. Therefore, ethical approval and informed consent were not required.

### Role of funding source

The funding sources were not involved in study design; in the collection, analysis, and interpretation of data; in the writing of the report; nor in the decision to submit the paper for publication.

## Results

### Model fitting

The 50 selected parameter sets closely replicate the observed calibration data on carcinogenic HPV prevalence by age group and sex in Colombia (Supplemental Figures S9–S11).

### Age-standardised HPV prevalence

In all vaccination scenarios, and at all vaccination coverage levels, the age-standardised prevalence of carcinogenic HPV declined in both female-only (Supplemental Figure S14) and gender-neutral (Supplemental Figure S15) vaccination scenarios. The decline was more pronounced with higher coverage, with a nonavalent vaccine compared to a quadrivalent vaccine, and with gender-neutral vaccination compared to female-only in the case of a nonavalent vaccine, but it was similar for a quadrivalent vaccine. Without a vaccination program, the model predicted 2.63 million cases of carcinogenic HPV infections among female population aged 15 years and older, and an age-standardised carcinogenic HPV prevalence of 20.1% (range: 19.0%–21.8%) by 2030, the WHO target year for achieving 90% vaccination coverage. Under a gender-neutral vaccination program with a nonavalent vaccine, the model estimated 2.09 million cases and an age-standardised prevalence of 14.9% (range: 13.5%–16.8%) at current vaccination coverage (51% in girls and 16% in boys), and 1.89 million cases with an age-standardised prevalence of 12.8% (range: 11.6%–14.8%) at 90% coverage. For a quadrivalent vaccine under a gender-neutral scheme, the model predicted 2.21 million cases and an age-standardised prevalence of 16.6% (range: 15.3%–18.4%) at current coverage; and 2.16 million cases with an age-standardised prevalence of 15.9% (range: 14.9%–17.5%) at 90% coverage by 2030 (Supplemental Table S9).

**Fig. 2:**
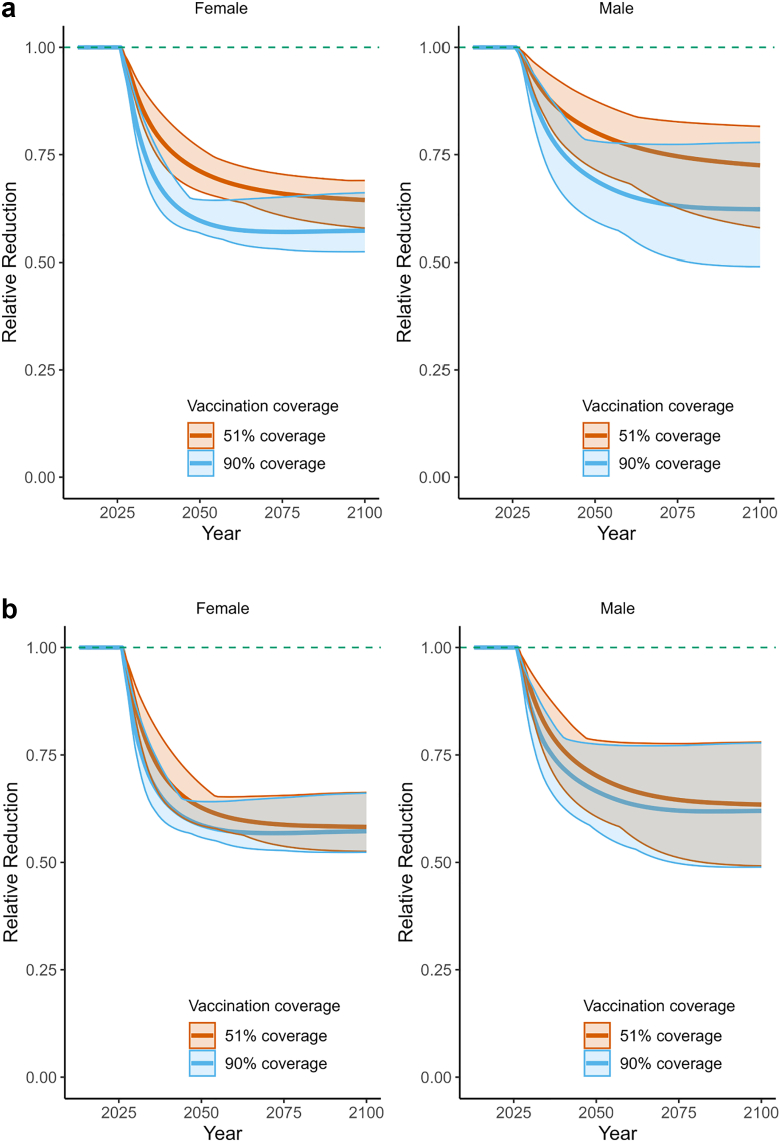
**Relative reduction in carcinogenic HPV prevalence with nonavalent HPV vaccine compared to quadrivalent vaccine: (a) female-only and (b) gender-neutral vaccination strategies, comparing a nonavalent and quadrivalent HPV vaccines at different vaccination coverage in Colombia from 2013 to 2100**. The thick lines represent the mean, and the shaded areas indicate the range of 50 simulations. 51% coverage represents the status quo and 90% coverage WHO target.

If Colombia were to switch to the nonavalent vaccine at current vaccination coverage, it would reduce carcinogenic HPV prevalence in females over 15 years old by 39% (prevalence ratio [PR]: 0.61; range: 0.54–0.67) and by 32% (PR: 0.68; range: 0.52–0.79) in males over 15 years old by 2100 (Table 2, Fig. 2) compared to a quadrivalent vaccine. On the other hand, if a quadrivalent vaccine is maintained while increasing the current gender-neutral vaccination coverage (raising it from 51% in girls and 16% in boys to 90% in both genders), there would be a 8% (PR: 0.92; range: 0.83–0.99) reduction in age-standardised carcinogenic HPV prevalence in girls and females over 15 years old, and 14% (PR: 0.86; range: 0.68–0.99) reduction in males over 15 years old by the end of the simulation compared with current coverage (Table 2).

**Table 2:** Prevalence ratio over time for all carcinogenic HPV,^a^ according to HPV vaccine type, vaccination scenario, and coverage.

Scenario	Vaccination schedule	Comparator	Female			Male		
			Year 2030	Year 2040	Year 2100	Year 2030	Year 2040	Year 2100
Girls-only	Nonavalent 51% coverage	No vaccination	0.76 (0.72–0.79)	0.61 (0.55–0.67)	0.48 (0.42–0.54)	0.84 (0.77–0.88)	0.71 (0.60–0.79)	0.54 (0.40–0.66)
Girls-only	Nonavalent 90% coverage	No vaccination	0.68 (0.64–0.72)	0.47 (0.43–0.53)	0.38 (0.34–0.43)	0.78 (0.70–0.83)	0.57 (0.45–0.65)	0.37 (0.29–0.50)
Gender-neutral	Nonavalent G51% & B16%	No vaccination	0.74 (0.70–0.77)	0.58 (0.52–0.64)	0.44 (0.38–0.51)	0.81 (0.74–0.86)	0.66 (0.54–0.74)	0.47 (0.33–0.58)
Gender-neutral	Nonavalent 51% coverage	No vaccination	0.71 (0.67–0.75)	0.52 (0.47–0.58)	0.39 (0.36–0.45)	0.77 (0.69–0.82)	0.59 (0.46–0.66)	0.39 (0.30–0.51)
Gender-neutral	Nonavalent 90% coverage	No vaccination	0.64 (0.60–0.69)	0.46 (0.42–0.51)	0.38 (0.34–0.43)	0.71 (0.63–0.76)	0.53 (0.42–0.60)	0.37 (0.29–0.49)
Girls-only	Quadrivalent 51% coverage	No vaccination	0.83 (0.81–0.85)	0.80 (0.77–0.83)	0.74 (0.68–0.79)	0.88 (0.81–0.92)	0.84 (0.75–0.90)	0.75 (0.62–0.84)
Girls-only	Quadrivalent 90% coverage	No vaccination	0.80 (0.78–0.81)	0.73 (0.70–0.75)	0.66 (0.60–0.70)	0.85 (0.79–0.90)	0.76 (0.67–0.83)	0.60 (0.48–0.75)
Gender-neutral	Quadrivalent G51% & B16%	No vaccination	0.90 (0.87–0.93)	0.74 (0.68–0.81)	0.61 (0.54–0.67)	0.94 (0.91–0.97)	0.82 (0.73–0.89)	0.68 (0.52–0.79)
Gender-neutral	Quadrivalent 51% coverage	No vaccination	0.81 (0.80–0.83)	0.76 (0.73–0.78)	0.67 (0.63–0.71)	0.85 (0.79–0.90)	0.77 (0.69–0.84)	0.62 (0.50–0.75)
Gender-neutral	Quadrivalent 90% coverage	No vaccination	0.79 (0.77–0.80)	0.72 (0.69–0.74)	0.66 (0.60–0.70)	0.83 (0.76–0.88)	0.73 (0.65–0.82)	0.59 (0.48–0.75)
Girls-only	Nonavalent 51% coverage	Quadrivalent 51% coverage	0.91 (0.89–0.94)	0.77 (0.72–0.83)	0.64 (0.58–0.69)	0.95 (0.93–0.98)	0.85 (0.78–0.92)	0.73 (0.58–0.82)
Girls-only	Nonavalent 90% coverage	Quadrivalent 90% coverage	0.85 (0.81–0.90)	0.65 (0.60–0.72)	0.57 (0.52–0.66)	0.92 (0.88–0.96)	0.75 (0.66–0.85)	0.62 (0.49–0.78)
Gender-neutral	Nonavalent G51% & B16% coverage	Quadrivalent G51% & B16% coverage	0.90 (0.87–0.93)	0.74 (0.68–0.81)	0.61 (0.54–0.67)	0.94 (0.91–0.97)	0.82 (0.73–0.89)	0.68 (0.52–0.79)
Gender-neutral	Nonavalent 51% coverage	Quadrivalent 51% coverage	0.87 (0.84–0.91)	0.69 (0.63–0.77)	0.58 (0.53–0.66)	0.90 (0.86–0.95)	0.76 (0.67–0.84)	0.63 (0.49–0.78)
Gender-neutral	Nonavalent 90% coverage	Quadrivalent 90% coverage	0.81 (0.76–0.87)	0.63 (0.59–0.69)	0.57 (0.52–0.66)	0.86 (0.80–0.92)	0.72 (0.63–0.79)	0.62 (0.49–0.78)
Girls-only	Nonavalent 90% coverage	Nonavalent 51% coverage	0.89 (0.86–0.93)	0.77 (0.72–0.84)	0.79 (0.68–0.92)	0.93 (0.89–0.95)	0.80 (0.72–0.87)	0.69 (0.53–0.88)
Gender-neutral	Nonavalent 90% coverage	Nonavalent G51% & B16% coverage	0.86 (0.82–0.91)	0.79 (0.74–0.86)	0.86 (0.73–0.97)	0.88 (0.82–0.92)	0.79 (0.72–0.89)	0.78 (0.58–0.95)
Gender-neutral	Nonavalent 90% coverage	Nonavalent 51% coverage	0.90 (0.87–0.94)	0.88 (0.84–0.93)	0.96 (0.85–1.00)	0.93 (0.89–0.95)	0.90 (0.85–0.96)	0.94 (0.78–1.00)
Girls-only	Quadrivalent 90% coverage	Quadrivalent 51% coverage	0.96 (0.94–0.97)	0.91 (0.87–0.94)	0.89 (0.80–0.96)	0.97 (0.94–0.98)	0.90 (0.83–0.95)	0.80 (0.63–0.96)
Gender-neutral	Quadrivalent 90% coverage	Quadrivalent G51% & B16% coverage	0.96 (0.93–0.97)	0.92 (0.88–0.96)	0.92 (0.83–0.99)	0.96 (0.92–0.98)	0.90 (0.82–0.96)	0.86 (0.68–0.99)
Gender-neutral	Quadrivalent 90% coverage	Quadrivalent 51% coverage	0.97 (0.95–0.98)	0.95 (0.92–0.98)	0.98 (0.91–1.00)	0.97 (0.95–0.99)	0.95 (0.90–0.98)	0.96 (0.84–1.00)

HPV, human papillomavirus; G, Girls; B, Boys.

The values correspond to the mean (range) of the 50 estimates from the parameter sets.

a Carcinogenic corresponds to infection with any of HPV types 16, 18, 31, 33, 35, 39, 45, 51, 52, 56, 58, 59, and 68.

The age-standardised carcinogenic HPV prevalence with female-only vaccination was slightly higher than the corresponding values observed with gender-neutral vaccination in scenarios of 51% coverage. However, in the case of 90% coverage, the difference between the two scenarios was negligible. Results for each group of HPV types and by age group are provided in the Supplement (Supplemental Figures S12, S13, S16 and S17). We observed similar results for age-standardised carcinogenic HPV incidence rates (Supplemental Tables S10 and S11, and Supplementary Figures S18–S21).

### Herd effects and breakthrough infections

For unvaccinated individuals, we observed a decrease in age-standardised prevalence of vaccine-type HPV over time due to herd effects, irrespective of the vaccination coverage, although it was more pronounced with 90% coverage, going from 7.5% (range: 5.8–10.8) in 2030 to 0.04% (range: less than 0.01%–0.12%) in 2100 with the nonavalent vaccine (Supplemental Table S12). Among vaccinated individuals, we also observed a reduction in age-standardised carcinogenic vaccine-type HPV prevalence in all scenarios but more markedly with 90% coverage of gender-neutral vaccination (from 0.12% to less than 0.01% between 2030 and 2100) due to herd effects reducing the risk of breakthrough infections.

### Projections for cervical cancer incidence

**Fig. 3:**
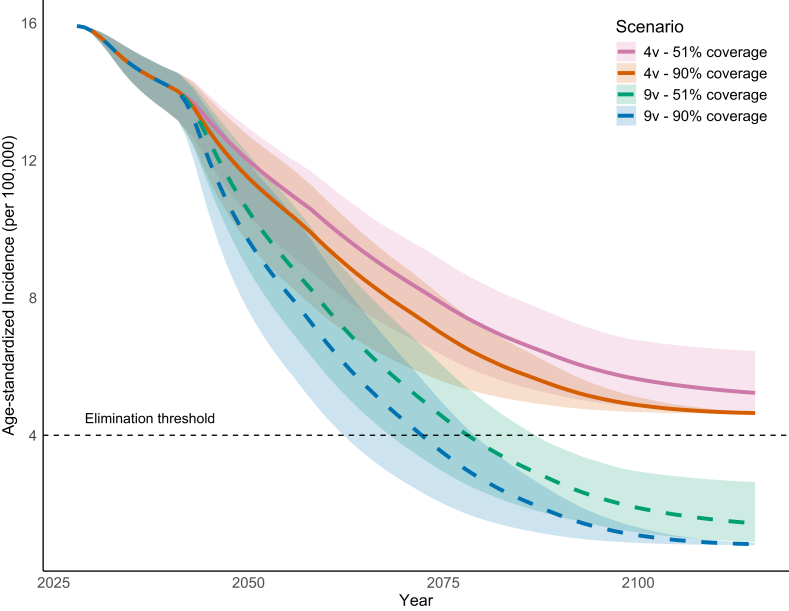
**Estimated age-standardised cervical cancer incidence projections in gender-neutral vaccination scenarios.** The thick lines represent the mean, and the shaded areas indicate the range of 50 simulations, considering age-specific prevalences and incidence rates. Age-standardisation was performed using the 2015 World Standard Population.^29^ 4v: quadrivalent HPV vaccine; 9v: nonavalent HPV vaccine.

Assuming no changes to screening practices, switching to a nonavalent vaccine led to cervical cancer incidence falling below the elimination threshold by 2058 (range: 2048–2064) with 90% vaccination coverage in a gender-neutral vaccination program, and by 2064 (range: 2054–2072) with 51% coverage (Supplemental Table S13 and Fig. 3). Similar trends were observed under the female-only vaccination scenario, although elimination was reached 2 and 18 years later, respectively (Supplemental Figure S22). Increasing the current coverage of a quadrivalent vaccine to 90% was unlikely to reach the elimination threshold within the modelled time horizon. A sensitivity analysis using the 1960 Segi/Doll world population age standard (Supplemental Table S14) showed similar results with elimination occurring slightly sooner. A sensitivity analysis accounting for waning of vaccine-induced immunity also did not show important differences from the main analysis (Supplemental Table S15).

## Discussion

Using a detailed transmission-dynamics model of HPV transmission and vaccination in Colombia, we found that, assuming no changes to the existing screening practices, to expedite progress toward achieving the cervical cancer elimination threshold of fewer than 4 cases per 100,000 females, Colombia would need to adopt a nonavalent HPV vaccine. The main additional benefit of a nonavalent vaccine is the prevention of infection with HPV 31, 33, 45, 52, and 58 (types not covered by a quadrivalent vaccine), as our model assumes no cross-protection from a quadrivalent vaccine. Moreover, if HPV vaccination coverage were increased to 90% for both boys and girls, the elimination threshold could be reached six years earlier than with current coverage. For the same reason, increasing vaccination coverage with a quadrivalent vaccine does not lead to a sufficient reduction in HPV infections to achieve cervical cancer elimination. Additionally, our model projected a decline in both the age-standardised carcinogenic HPV prevalence and incidence among unvaccinated populations due to herd immunity, with more substantial reductions observed at 90% vaccination coverage. Under the 90% coverage scenarios, HPV transmission is reduced to very low levels, leading to convergence of model trajectories and a progressive narrowing of uncertainty bands over time, which reflects convergence toward elimination rather than fixed demographic assumptions.

Several modelling studies conducted in high-income countries predicted large decreases in HPV prevalence following the switch to a nonavalent vaccine and increased vaccination coverage, similar to our findings.^20^^,^^21^^,^^63^, ^64^, ^65^, ^66^, ^67^, ^68^, ^69^ Our estimates on the relative reduction of carcinogenic HPV prevalence and cervical cancer incidence were similar to those predicted for Poland by Jakubczyk et al.,^67^ but lower than those reported in other studies.^21^^,^^64^^,^^68^ These differences could be attributed to variations in the model's characteristics and assumptions. First, models differed in how they consider sexual partnership mixing, including rates of new partner acquisition and the distribution of the population across sexual activity levels, which can have substantial impacts on HPV transmission. Second, while several previous models assumed 100% vaccine efficacy, we incorporated slightly lower vaccine efficacy (from 87.4% to 98.0%), as reported in clinical trials. Third, barring a few exceptions (e.g. van Schalkwyk 2019)^70^ nearly all previous models had not accounted for the possibility of HPV latency and reactivation, which could lead to overly optimistic projections and earlier predicted timelines for cervical cancer elimination. A key innovation of our study was to include these features. Substantial evidence, published after many of these models were developed, supports that following an active infection, HPV infection may remain with a viral load below the limit of detection and hence be undetected. Reactivations can occur when the host's immune response changes.^71^ It has been hypothesised that the increase in HPV prevalence seen in older females may be due to reactivation of previous infections rather than new infections.^72^ Accordingly, van Schalkwyk et al., have shown that failing to consider latency can lead to an overestimation of HPV vaccine effectiveness.^70^ Consistent with this, our model also predicted smaller reductions in carcinogenic HPV prevalence compared to models that assessed a quadrivalent vaccine under similar female-only vaccination coverage levels.

Considering that, on average, it takes between 15 and 20 years for a carcinogenic HPV infection to progress into cervical cancer,^73^ our model estimates that Colombia could potentially achieve cancer elimination between 2048 and 2064 if high coverage levels of a nonavalent vaccine are rapidly attained for both girls and boys by age 15, while maintaining current screening practices. These results are consistent with findings from other models of generic populations in low- and medium-Human Development Index (HDI) countries.^74^^,^^75^ Simms et al.^74^ reported that an extremely rapid and effective scale-up of prevention interventions will be necessary to reduce cervical cancer incidence below the threshold of 4 cases per 100,000 females-year before the end of the century. The authors reported that a combination of high-coverage nonavalent vaccination and two lifetime HPV DNA screenings would reduce cervical cancer incidence to below this threshold in medium-HDI countries between 2070 and 2079, and in low-HDI countries between 2090 and 2100. On the other hand, with a more gradual scaled-up vaccination and screening, cervical cancer incidence would decline to 4.4 cases per 100,000 females-year in medium-HDI countries, but only to 14 cases per 100,000 females-year in low-HDI countries by the end of the century. Similarly, Brisson et al.^75^ predicted that female-only vaccination against HPV 16, 18, 31, 33, 45, 52, and 58 with 90% coverage and lifetime protection would reduce the median age-standardised cervical cancer incidence in low- and middle-income countries from 19.8 to 2.1 cases per 100,000 females-years over the next century. We predicted that female-only vaccination in Colombia, based on a 90% coverage with a nonavalent vaccine, would reduce the age-standardised cervical cancer incidence from 13.7 to 0.74 cases per 100,000 females-years by 2100.

Our model shows that, compared to female-only vaccination, gender-neutral vaccination could further accelerate the decline in carcinogenic HPV prevalence and, consequently, in cervical cancer incidence, consistent with previous reports.^22^^,^^76^ However, the magnitude of this effect likely depends on achieving and maintaining high vaccination coverage, which may be more challenging in LMIC such as Colombia. Furthermore, the relative reduction in carcinogenic HPV prevalence largely overlaps across vaccination coverage levels, indicating that increasing coverage has a modest incremental effect compared with switching vaccine type, particularly under gender-neutral vaccination. We also found that the impact of switching to a nonavalent vaccine in a gender-neutral vaccination program is greater when vaccination coverage is low (as it increases the number of vaccinated individuals), relative to female-only vaccination, aligning with earlier findings.^19^^,^^77^ Colombia faces particular challenges in increasing HPV vaccination coverage, even with one-dose schedules, due to low vaccine confidence.^7^^,^^8^ As a result, alternative strategies may be worth pursuing. One such strategy was the recent inclusion of boys in the vaccination program. Although current coverage among boys remains low (around 16%), this likely reflects the early phase of program implementation, as vaccination only began in the second half of 2024; therefore, there has been limited time to achieve higher uptake.

There are some limitations to our analysis. First, we did not directly model the natural history of progression from HPV infection to cervical cancer. Instead, we have estimated the necessary reduction in carcinogenic HPV prevalence required to achieve cervical cancer elimination, considering age-specific cancer incidence, HPV prevalence, and HPV type distribution in our population. Given the well-established link between HPV infection and cervical cancer, this simplification is unlikely to affect long-term predictions. However, some uncertainty may remain regarding the timing of the decline in cervical cancer incidence, which would not be fully captured by a model that does not explicitly include the transition from HPV to lesion development. Second, the natural history of HPV infection includes several stages where progression to cancer can be interrupted through screening and appropriate management of precancerous lesions. Our model only accounts for vaccination; incorporating cervical cancer screening and treatment of screen-detected lesions would likely accelerate the achievement of the elimination threshold. Because we focus on modelling HPV transmission, our model is well suited to address questions related to vaccination impact, but not to explore strategies for improving cancer screening practices. Third, due to the lack of detailed sexual behaviour data for Colombia, we relied on parameters priors derived from the non-English-speaking Hispanic population in the U.S. National Survey of Family Growth as a proxy. This assumption may introduce bias, as partner acquisition rates and assortative mixing patterns could differ from those in Colombia. Nevertheless, we calibrated the model to Colombian HPV prevalence, therefore the selected parameter sets include sexual behaviour values that reproduce HPV dynamics in Colombia. While future studies would benefit from country-specific sexual behaviour data or formal sensitivity analyses, we expect the main conclusions to be robust to plausible variation in these assumptions. Fourth, we used 10,000 parameter sets for calibration, which allowed good reproduction of calibration targets; however, larger samples could further refine posterior uncertainty estimates. Fifth, our study, did not account for the impact of human immunodeficiency virus (HIV) on HPV transmission, progression or reactivation. Nevertheless, we consider that including these would not qualitatively affect our results, as HIV prevalence among 15–49-year-olds in Colombia is less than 1%, similar to the rest of Latin America (values between 0.2% and 0.7%), with the exception of Panamá.^78^ Because HPV types were modelled in groups rather than individually, the model does not allow concurrent or sequential acquisition of multiple types within a group during periods of latency; the consequence of this would be more conservative estimates due to lower herd effects with a higher force of infection.

Despite limitations, our study has several methodological strengths. First, we developed a model specifically calibrated to the available demographic and epidemiological data from Colombia. Detailed country-level analyses that consider specific local factors affecting vaccination implementation are key to maximising the use of scarce resources and should be viewed as important complements to global and regional analyses. Second, through the calibration process, we selected 50 sets that best fit the epidemiological data on carcinogenic HPV prevalence. This allowed us to capture the uncertainty around key parameters for which empirical data are lacking or incomplete, thereby enhancing the robustness of our results.^79^^,^^80^ Third, we developed a transmission dynamic component, which allowed us to capture herd effects, different from previous work in the region.^81^, ^82^, ^83^, ^84^ Finally, unlike other models^21^^,^^74^^,^^85^ that assume 100% vaccine efficacy at preventing HPV acquisition, we assumed a lower efficacy, where vaccination reduces—but does not completely prevent—HPV transmission, allowing for measurable breakthrough infections.

Future research should incorporate screening and precancer outcomes to evaluate the cost-effectiveness of transitioning to a nonavalent vaccine. As in most HPV vaccination modelling studies, we assumed no changes in cervical cancer screening to isolate the effects of vaccination policies; however, results should be interpreted in this context, as future screening changes could modify the absolute impact of vaccination on cervical cancer outcomes. In scenarios of vaccination coverage lower than 90%, screening plays an important role in identifying precancerous lesions in unvaccinated females and preventing cancer development. Increases in screening coverage, adherence to screening program recommendations, or switching to a high-performance test such as HPV DNA would further accelerate the elimination of cervical cancer. The Pan American Health Organization Revolving Fund,^86^ can greatly contribute to keeping costs low and making the vaccine more cost-effective. Colombia has experienced vaccination coverage fluctuations since the start of the program; moreover, achieving and maintaining high vaccination coverage, such as 90%, is challenging for most countries. However, our results indicate that switching to the nonavalent vaccine yields substantial reductions in HPV prevalence even when current coverage levels are maintained, suggesting that the projected benefits are robust to uncertainty in long-term vaccination coverage. Regarding vaccine characteristics, we assumed lifelong immunity and a constant vaccination coverage. Although current evidence does not suggest a decline in protection following single-dose vaccination, future analyses could explore waning immunity scenarios should empirical data indicate such a trend. We acknowledge that assuming non-waning vaccine-conferred immunity may influence longer-term extrapolations beyond existing follow-up data. Nevertheless, our sensitivity analysis also showed that cervical cancer elimination was potentially achievable with the nonavalent vaccine.

In summary, our study found that, to expedite progress toward achieving the WHO cervical cancer elimination threshold—assuming no changes to screening—switching to a nonavalent vaccine is required to sufficiently reduce carcinogenic HPV infections. When evaluating new interventions for cervical cancer prevention, stakeholders should prioritise country-specific evidence, such as the findings presented here, while also considering the programmatic feasibility and acceptability of such interventions. Although our model was calibrated to the specific context of Colombia, policymakers from other South American countries may find our results relevant, particularly in settings where populations share similarities in sexual behaviour, HPV type distribution, age profile of HPV prevalence, and cervical cancer incidence, and especially in countries where vaccination coverage has stagnated despite multiple efforts to increase it.

## Contributors

RT and TM conceptualised the study and developed the mathematical model. RT conducted the formal analysis and visualisation. AB, MA, JR and LT provided parameters necessary for the model. Parameters, assumptions, and analyses were validated by all authors. TM, MMG and ELF supervised the work and contributed to the interpretation of the results. RT prepared the original draft of the manuscript, and all authors contributed to the writing, review, and editing. All authors had access to the underlying data, verified the results, and approved the final version of the manuscript.

## Data sharing statement

No new primary data was collected for this simulation study. The values of the parameters used in the model have been provided in the Supplement. Methodology details are available in the supplemental material. Results from model simulations, the code for projections on cervical cancer incidence, and further documentation on the model structure can be accessed on the Borealis repository (https://doi.org/10.5683/SP3/PD1CKX).

## Disclaimer

MA is a staff member of the World Health Organization. The author alone is responsible for the views expressed in this article and they do not necessarily represent the decisions, policy or views of the World Health Organization. The funding sources were not involved in study design; in the collection, analysis, and interpretation of data; in the writing of the report; nor in the decision to submit the paper for publication.

## Declaration of interests

Through his institution, ELF has received supplemental grants from Merck in support of investigator-initiated studies that he led and that were also funded by Canadian agencies. He has also served as an occasional advisor to Merck on matters related to HPV vaccination. ELF holds a patent on methylation for cervical cancer screening. JR and LT received fees as speakers for MSD vaccines, LT received travel support from MSD. RT has received honoraria from the Society of Gynecologic Oncology of Canada and travel support from McGill University and Instituto Nacional de Salud Pública, Mexico. TM is a previous board member of the International Papillomavirus Society and has received travel support from them. The other authors have no relevant financial or non-financial interests to disclose.

## Appendix A Supplementary data

Supp_Impact of switching to 9vHPV in Colombia
