# Supplementary material for: Impact of switching from a quadrivalent to a nonavalent HPV vaccine on HPV infections and cervical cancer in Colombia: a mathematical modelling study
Source: Lancet Reg Health Am. 2026 May 5;58:101483. doi: 10.1016/j.lana.2026.101483 (PMC13158420; doi:10.1016/j.lana.2026.101483)
Supplement: Supp_Impact of switching to 9vHPV in Colombia [file mmc1.pdf]

Impact of switching from a quadrivalent to a nonavalent HPV vaccine on HPV infections and cervical cancer in Colombia: a mathematical modeling study

Romina Tejada, Armando Baena, Lina Trujillo, Juliana Rodriguez, Mathieu Maheu-Giroux, Maribel Almonte, Eduardo L. Franco, Talía Malagón

Supplementary material

Correspondence to Romina A. Tejada at romina.tejada@mail.mcgill.ca

Table of contents

MODEL DESCRIPTION 3
Model structure 3
Demographic characteristics 3
Natural history of HPV infection 3
Simplified ordinary differential equations showing transitions between states 5
Equations for the event function 6
Sexual partnership formation 7
Infection transmission 9
HPV vaccination 9
MODEL VARIABLES AND PARAMETERS 9
Demography 9
HPV natural history parameters 13
Vaccination parameters 16
Sexual behavior parameters 17
CALIBRATION 23
Prevalence studies 23
Burn-in simulation to obtain initial state values 23
Latin hypercube sampling 23
Selection of best fitting sets 27
ANALYSIS 31
Combination of groups of HPV types 31
Cervical cancer projections 31
RESULTS 32
Age-standardized HPV prevalence 32
Age-standardized HPV incidence 32
Breakthrough infections and herd immunity 52
Age-standardized cervical cancer incidence 53
REFERENCES 56

MODEL DESCRIPTION

Model structure

We developed a model of HPV transmission and vaccination; this model builds upon a framework previously developed by Malagon et al. [1] Our model is a compartmental, ordinary differential equation, transmission dynamic model programmed in R using the package deSolve. [2] The model simulates a population of heterosexual individuals stratified according to state *i* (not sexually active, susceptible, infected, latent, and recovered), sex *j* (female and male), age *m* (70 one-year age groups between 15 to 84 years and one age group for 85+ years old), sexual activity level *l* (low and high), and vaccination status *k* (vaccinated and unvaccinated). The model is programmed to run over calendar time and includes event functions to model demographic events. Individuals age in 1-year increments over calendar time and includes event functions to model demographic events. The population is open, with new cohorts of 15-year-olds entering the model each year, and individuals dying at an age- and sex-specific all-cause mortality rates (μ_{jm}) derived from the UN projections specific to Colombia (see **Mortality rate projections from all causes by age and sex** section). [3]

Demographic characteristics

Each year, individuals enter the model at age 15 (based on UN estimates and projections[3]) into either the "Not sexually active" or "Susceptible" states, depending on the proportion of individuals sexually active at age 15 ($\tau_{j,m=15}$) (see **Proportion of sexually active individuals** section). These individuals are assigned a vaccination status, and sexual activity level at entry, including individuals who are not yet sexually active (this represents the sexual activity level they will have once they become sexually active). Sexual activity level (see **Sexual activity level** section) was categorized as low or high, based on the number of sexual partners of the opposite sex in the past year (≤2, >2). Sexual activity level can change throughout a person's life, when they transition between the age groups 15-19, 20-24, 25-44, and 45+ (see **Probability of changing sexual activity level** section). At the start of each year, all individuals are aged by 1 year using an event function (see **Equations for the event function** section), except for those in the oldest age group (85+ years old) who remain in the same age group.

Natural history of HPV infection

We modeled the natural history of HPV infection considering five states (*i*) Not sexually active, (*ii*) Susceptible, (*iii*) Infected, (*iv*) Latent infection, and (*v*) Recovered. We illustrate in Figure S1 the natural history transitions between these states. The "Not sexually active" state includes individuals who are not yet sexually active and are not at risk for HPV infection; once they become sexually active at a rate η_{jm} (see **Rate of becoming sexually active** section) they transition to the "Susceptible" state. Fifteen-year-olds enter the model already vaccinated according to the sex-specific vaccination probability α_j defined for each scenario (ranging from 0% in no vaccination scenarios to 90% in line with the WHO vaccination target and including reported coverage levels for Colombia). Vaccination is implemented as a state stratification; thus, the natural history of infection and disease progression is identical for vaccinated and unvaccinated individuals, with differences arising only from the reduced rate of infection among those vaccinated. The rate of infection (λ_{jklm}) of susceptible vaccinated and unvaccinated individuals is detailed in the section **Infection transmission**. Individuals with an active HPV infection become HPV-negative at a rate γ_j. It is well established that loss of HPV detectability can represent several natural history events which we model as possible outcomes following an active infection: individuals could develop natural immunity with a sex-specific probability σ_j, develop a latent infection with a probability π, or clear the infection and become susceptible again. Latent infections can reactivate at a rate κ. Natural immunity wanes at a rate ω_j. Individuals whose natural immunity has waned return to the "Susceptible" state, where they are again at risk of HPV infection, at the same rate as individuals who have never been infected.

The model was fit independently to natural history parameters for three oncogenic groups of HPV type that have been associated with cervical cancer: (*i*) HPV16 and 18; (*ii*) HPV31, 33, 45, 52, and 58; and (*iii*) non vaccine carcinogenic HPV (e.g. HPV 35, 39, 51, 56, 59, and 68). We considered HPV groups to be independent; we did not consider possible interactions in transmission dynamics of different genotypes. Results were later combined to determine changes in overall carcinogenic HPV infection by weighting results from each group of HPV type by empirical coinfection distributions. The third group representing non vaccine carcinogenic HPV types was included to explicitly account for cervical cancers not preventable by current vaccines and to ensure that HPV prevalence and transmission dynamics reflect the full spectrum of carcinogenic infections when weighting reductions in cervical cancer incidence by HPV type distribution. Moreover, including this group allows for modeling the impact of HPV vaccines including additional carcinogenic HPV types in the future.

This approach is not equivalent to a fully strain-structured model, as it does not explicitly capture simultaneous infection or competing risks across multiple HPV type groups within the same individuals. Nevertheless, it was necessary to reduce model complexity and computational burden.

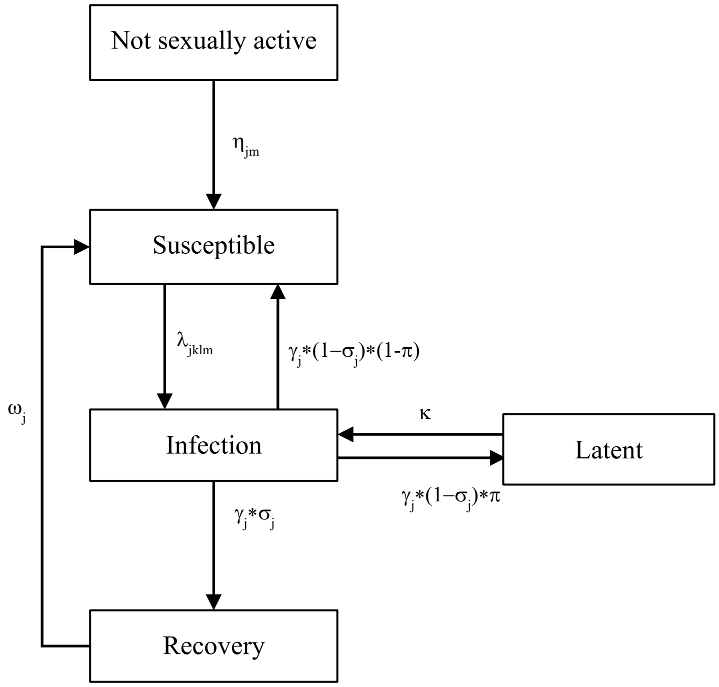

Figure S1. Dynamic transmission model for HPV infection.
η_{jm}: rate of becoming sexually active by sex j and age m, λ_{jklm}: force of infection by sex j, sexual activity level l and age m, γ_j: rate of loss of infection positivity rate by sex j, σ_j: probability of infections conferring natural immunity by sex j, π: probability of becoming latent infection, κ: reactivation rate, ω_j: rate of waning of natural immunity rate by sex j. This figure only illustrates the ODE natural history part of the model and does not include the demographic event functions.

Simplified ordinary differential equations showing transitions between states

The following are the differential equations (equations 1 to 5) for the transitions between states; they do not include transitions between strata (e.g., from low to high sexual activity level and vice versa) in the model. Population entries, deaths, ageing, and change in sexual activity level are not modeled as rates in the differential equations but rather using a forcing function (See next section **Equations for Events function**) or as proportions of entries in a specific stratum (e.g. vaccination). Therefore, they are not included in this section. A list of the parameters used in the differential equations is provided in Table S1.

Table S1. Notation for sexual behavior and states, and for natural history parameters.

Notation	Explanation
NSA_{jklm}	Not sexually active individuals
S_{jklm}	Susceptible individuals
I_{jklm}	Individuals infected with HPV
L_{jklm}	Individuals with latent HPV infection
R_{jklm}	Recovered individuals
η_{jm}	Rate of becoming sexually active
λ_{jklm}	Rate of infection
γ_j	Rate of loss of HPV positivity
σ_j	Proportion of infections conferring natural immunity
ω_j	Waning rate of natural immunity
π	Proportion of HPV infections becoming latent
κ	Rate of reactivations of latent HPV infections

Not sexually active: $\frac{dNSA_{jklm}}{dt} = -NSA_{jklm} * \eta_{jm}$; (1)

Susceptible: $\frac{dS_{jklm}}{dt} = NSA_{jklm} * \eta_{jm} + R_{jklm} * \omega_j + I_{jklm} * \gamma_j(1-\sigma_j)(1-\pi) - S_{jklm} * \lambda_{jklm}$; (2)

Infected: $\frac{dI_{jklm}}{dt} = S_{jklm} * \lambda_{jklm} + L_{jklm} * \kappa - I_{jklm} * \gamma_j$; (3)

Latent infection: $\frac{dL_{jklm}}{dt} = I_{jklm} * \gamma_j(1-\sigma_j)\pi - L_{jklm} * \kappa$; (4)

Recovered: $\frac{dR_{jklm}}{dt} = I_{jklm} * \gamma_j\sigma_j - R_{jklm} * \omega_j$; (5)

Equations for the event function

There are four events in our model that occur at discrete times (once a year) contrary to the differential equations that occur in a continuous time. These are deaths, ageing, entries, and change in sexual activity level (equations 6 to 12). For these, we used the events function option in the *ode* function to force the model to manually carry out these changes. A list of the parameters used in the event function is provided in Table S2.

Table S2. Model parameters for the event function.

Notation	Explanation
$\mu_{j,m,t}$	Mortality rate by year (t)
α_j	Vaccination coverage
$\theta_{j,l,m}$	Proportion of individuals in sexual activity level
$\tau_{j,m}$	Proportion of individuals sexually active
$\phi_{j,m}$	Probability of changing sexual activity level
i	Index for state (NSA, S, I, R, L)
j	Index for sex 1= female 2=male
k	Index for vaccination status 1=vaccinated 2=unvaccinated
l	Index for sexual activity level 1=low and 2=high
m	Index for age from 15 to 85+ years old
t	Index for time in years
StartDate	Simulation start time, assigned as 2010
$states_{i,j,k,l,m,t}$	State of the system at indices i, j, k, l and m at time t. It represents the entire population in the strata
$15yo_{j,t}$	Population of 15 years old entering the model in year t

Deaths

All individuals could die at a mortality rate by year, age and sex (μ_{jmt}) based on the United Nations projections (equation 6).[4]

$$states_{i,j,k,l,m,t+1} = states_{i,j,k,l,m,t} - states_{i,j,k,l,m,t} * \mu_{j,m,t} \quad ; (6)$$

Entries

A population of 15 years-olds[3] enters the model each year and is distributed between the "Not sexually active" and "Susceptible" states (equations 7 to 10), depending on the proportion of sexually activity by sex at age 15 ($\tau_{j,m=15}$). These individuals have a vaccination coverage (α_j) and sexual activity level ($\theta_{j,l,m}$) assigned at entry (even if not sexually active).

If vaccinated (k=1)

$$states_{i=NSA,j,k=1,l,m=15,t+1} = 15yo_{j,t+1} * \alpha_j * \theta_{j,l,m=15} * (1 - \tau_{j,m=15}) \quad ; (7)$$
$$states_{i=S,j,k=1,l,m=15,t+1} = 15yo_{j,t+1} * \alpha_j * \theta_{j,l,m=15} * \tau_{j,m=15} \quad ; (8)$$

If unvaccinated (k=2)

$$states_{i=NSA,j,k=2,l,m=15,t+1} = 15yo_{j,t+1} * (1 - \alpha_j) * \theta_{j,l,m=15} * (1 - \tau_{j,m=15}) \quad ; (9)$$
$$states_{i=S,j,k=2,l,m=15,t+1} = 15yo_{j,t+1} * (1 - \alpha_j) * \theta_{j,l,m=15} * \tau_{j,m=15} \quad ; (10)$$

Ageing

After deaths occur, all individuals transition from one age to the next, except for the 85+ years old, who will remain in the group and receive the individuals from the previous age (equations 11 and 12). The final age distribution of the simulated population is shown in Figure S3.

If 15 < m < 85, then

$$states_{i,j,k,l,m,t+1} = states_{i,j,k,l,m-1,t} \quad ; (11)$$

If m = 85, then

$$states_{i,j,k,l,m=85,t+1} = states_{i,j,k,l,m=85,t} + states_{i,j,k,l,m=84,t} \quad ; (12)$$

Change in sexual activity level

Changes in the sexual activity level occur in the population at ages 20, 25 and 45, because sexual activity level was calculated by age groups using these cut-off ages (equations 13 and 14). Individuals may go from the low

to the high sexual activity level and vice versa, based on a probability of changing sexual activity level by sex and age ($\phi_{j,m}$). Although equations 11 and 12 are written in a single direction, transitions from low to high sexual activity levels are also captured, as the parameter $\phi_{j,m}$ can take negative values. In practice, this formulation allows changes in both directions, but due to epidemiological patterns, most transitions occur from high to low as the population ages.

$$states_{i,j,k,l=2,m,t+1} = states_{i,j,k,l=2,m,t} - \phi_{j,m} * states_{i,j,k,l=2,m,t} \quad ;(13)$$

$$states_{i,j,k,l=1,m,t+1} = states_{i,j,k,l=1,m,t} + \phi_{j,m} * states_{i,j,k,l=2,m,t} \quad ;(14)$$

Sexual partnership formation

We modeled sexual partnership formation (see Table S3 for Notation on sexual partnership formation). This is an instantaneous partnership model, meaning that there is one per-partnership instantaneous probability of transmission. Most evidence suggests that HPV transmission occurs primarily at the beginning of sexual partnerships due to its very high transmissibility. Therefore, this simplification should lead to realistic results over the timescales we model while also reducing the computational burden. [5] There is no memory of previous sexual partnerships, and mixing does not depend on individuals' states, except for the "Not sexually active" state which has no partner acquisition rates. The parameters related to sexual partners are indicated by an apostrophe (´) in the equations.

Table S3. Notation for sexual partnership formation formula parameters.

Notation	Explanation
$\rho_{jlmj'l'm'}$	Probability that an individual of sex j, sexual activity level l and age m forms a sexual partnership with an individual of the opposite sex j´, sexual activity l´ and age m´
$\delta_{mm'}$ / $\delta_{ll'}$	Kronecker delta, has a value of 1 when m=m' or l=l' and 0 otherwise,
ε_{age}	Assortativity parameter for age; proportion of partnerships that are exclusively within same age group
ε_{sex}	Assortativity parameter for sexual activity level; proportion of partnerships that are exclusively within the same sexual activity level
$P_{j'l'm'}$	Total number of partnerships offered by people of sex j', sexual activity level l and age m
n_A	Number of age groups
n_S	Number of sexual activity groups
$D_{jlmj'l'm'}$	Desired partnership formation, i.e., the number of partnership that individuals in each sex want to form
$B_{jlmj'l'm'}$	Imbalance between supply and demand of sexual partners
υ	Compromise parameter to weight desired partnership formation for each sex
$SexAct_{jlm}$	Number of sexually active individuals in the population (sum of Susceptible, Infected, Latent and Recovered)
c_{jlm}	New partner acquisition rate
$c^*_{jlm\,j'l'm'}$	New partner acquisition rate with individuals of opposite sex j' of sexual activity l' and age m', corrected for imbalances in supply and demand

We followed Walker et al.'s proposed equations for sexual partnership formation and Garnet and Anderson formulas for imbalance correction. [6,7] The formation of sexual partnerships is a function of the overall desired new partner acquisition rate (c_{jlm}) considering the imbalance between male and female partnerships that are available ($B_{jlmj'l'm'}$). We describe hereafter the different components and present the equations for their calculations. First, the probability that an individual of sex j, sexual activity level l and age m, forms a sexual partnership with an individual of the opposite sex j´, age m´ and sexual activity l´ is $\rho_{jmlj'm'l'}$ (equation 15), as described by Walker et al. [6] This probability depends on the number of sexual partnership available ($P_{j'l'm'}$) and the assortativity parameters $\varepsilon_{j\text{-}age}$ and ε_{sex}. The Kronecker delta ($\delta_{mm'}$ for age and $\delta_{ll'}$ for sexual activity level) has a value of 1 when both partners have the same age (m=m') or the same sexual activity level (l=l') and 0 otherwise. These assortativity parameters are the proportion of sexual partnerships that are made exclusively with individuals of the same age group stratified by sex ($\varepsilon_{j\text{-}age}$) or same sexual activity level (ε_{sex}), while the remaining sexual partnerships ($1-\varepsilon_{j\text{-}age}$ & $1-\varepsilon_{sex}$) are made proportionately to the number of sexual partnerships available from each age group or sexual activity level. The population is stratified into 14 age groups for assortativity by age: 15 to 19 years, 20 to 24 years, 25 to 29 years, 30 to 34 years, 35 to 39 years, 40 to 44 years, 45 to 49 years, 50 to 54 years, 55 to 59 years, 60 to 64 years, and 65 to 69 years, 70 to 74 years, 75 to 79 years, and 80 years or more.

$$\rho_{jlmj'l'm'} = \varepsilon_{age}\varepsilon_{sex}\delta_{mm'}\delta_{ll'} + \varepsilon_{age}\,(1-\varepsilon_{sex})\delta_{mm'}\frac{P_{j'm'l'}}{\sum_{\beta=1}^{n_S} Pj'm'\beta} + (1-\varepsilon_{age})\varepsilon_{sex}\delta_{ll'}\frac{P_{j'm'l'}}{\sum_{\alpha=1}^{n_A} Pj'\alpha l'} + (1-\varepsilon_{age})(1-\varepsilon_{sex})\frac{P_{j'm'l'}}{\sum_{\alpha=1}^{n_A}\sum_{\beta=1}^{n_S} P\alpha\beta} \quad ;(15)$$

Secondly, the desired number of sexual partnerships ($D_{jlmj'l'm'}$, equation 16) considers the initial new partner acquisition rate (c_{jlm}) by sex j, sexual activity level l and age m (see **New partner acquisition rate** section), the probability of forming a sexual partnership ($\rho_{jmlj'm'l'}$), and the number of sexually active individuals in the population ($SexAct_{jlm}$; equation 17).

$$D_{jlmj'l'm'} = c_{jlm} * \rho_{jlmj'l'm'} * SexAct_{jlm} \quad ;(16)$$

$$SexAct_{jlm} = S_{jlm} + I_{jlm} + L_{jlm} + R_{jlm} \quad ;(17)$$

Thirdly, the difference between the desired number of sexual partnerships in females ($D_{FlmMl'm'}$) and in males ($D_{MlmFl'm'}$) creates an imbalance ($B_{jlmj'l'm'}$, equation 18) in desired partnership formation.

$$B_{jlmj'l'm'} = \frac{D_{FlmMl'm'}}{D_{MlmFl'm'}} \quad ;(18)$$

Finally, we balanced the desired number of sexual partnerships between sexes, sexual activity levels, and ages using the method described by Garnet and Anderson, [7] where new partner acquisition rates are adjusted ($c^*_{jlm\,j'l'm'}$) by the imbalance between females and males partnership formation desires ($B_{jlmj'l'm'}$) using a compromise

parameter υ (equation 19), where υ = 1 if females´ desires determine sexual partnership formation, υ = 0 if males´ desires determine sexual partnership formation; and a value between 0 and 1 represents a compromise between both preferences. These balancing parameters enable reconciling differences that exist in surveys regarding the number of sexual partners reported by females and males, as well as balancing supply and demand of partnerships in the model.

$$c^{*}_{jlmj'l'm'} = c_{jlm} * B^{\upsilon}_{jlmj'l'm'} \quad ; (19)$$

Infection transmission

Susceptible individuals can only be infected while they are in a sexual partnership with an infected partner. The rate of infection (λ_{jklm}, equation 20) depends on the rate of contact with infectious partners (Ic_{lm}), probability of infection by sex j (β_j), and vaccine efficacy by sex j and vaccination status k (χ_{jk}). Vaccine efficacy is, therefore, modeled as a per-partnership reduction in transmission. The rate of contact with infectious partners (equation 21) was calculated using the prevalence of HPV infection in the sexually active population, the corrected new partner acquisition rate by sex ($c*_{jlmj'l'm'}$), and the probability of sexual partnership formation ($\rho_{jmlj'm'l'}$).

$$\lambda_{jklm} = Ic_{lm} * \beta_j * (1 - \chi_{jk}) \quad ; (20)$$

$$Ic_{lm} = \sum_{l'} \sum_{m'} \left[\frac{\sum I_{j'l'm'}}{SexAct_{j'l'm'}} * c^{*}_{jlmj'm'l'} * \rho_{jmlj'm'l'} \right] \quad ; (21)$$

HPV vaccination

Vaccination-derived immunity is assumed to be lifelong. We assumed that vaccination occurs before sexual debut and before entry in the model since WHO recommendations are to target ages prior to sexual debut. [8] HPV vaccines are prophylactic, they do not affect the natural history of HPV infection, or related diseases, among individuals infected by an HPV type pre-vaccination. The vaccinated population is still susceptible to other carcinogenic HPVs not included in the vaccine (either quadrivalent or nonavalent). Depending on a vaccination coverage rate (α_j), the population enter the model in a vaccinated or unvaccinated status. Unlike many models that consider that the vaccinated population cannot be infected, we assumed that vaccination works by decreasing the risk of HPV transmission per partnership depending on the vaccine efficacy (χ_{jk}), allowing for breakthrough infections.

MODEL VARIABLES AND PARAMETERS

Demography

For demographic parameters, we provide parameters for Colombia as a case example. These values could be changed depending on the country in future research.

Initial population size

The model started with a population of 15 to 85+ years old based on the United Nations (UN) World Population Prospects for 2010 for Colombia (Table S4). [3]

Fifteen years old population entering the model

Each year, from 2011 to 2100, a new cohort of 15-year-olds enters the model (Table S5). The number of 15-year-olds entering the model corresponds to the UN population estimations (2011 to 2021) and projections (2022 to 2100) for Colombia based on medium fertility variant. [3]

Table S4. Initial population by age and sex. [3]

Age (years)	Females	Males	*Cont'd*	Age (years)	Females	Males
15	424091	438163		51	253780	234956
16	424995	438484		52	243125	224561
17	424359	437171		53	232253	214048
18	422273	434482		54	221476	203756
19	418850	430449		55	210877	193815
20	414191	424748		56	200563	184209
21	408995	418143		57	190474	174826
22	403682	411449		58	180544	165602
23	398520	404961		59	171397	157103
24	393236	398280		60	162571	148843
25	388166	391590		61	151584	137297
26	383159	384855		62	141896	127022
27	378529	378477		63	134882	120334
28	373499	371649		64	127415	113009
29	367128	363478		65	120099	105763
30	360603	355325		66	113007	98725
31	352947	346277		67	106120	91917
32	344393	336469		68	99489	85422
33	336361	327275		69	93194	79330
34	328697	318489		70	87191	73584
35	321911	310582		71	81403	68082
36	316838	304365		72	76122	63069
37	313370	299833		73	71545	58710
38	311451	297071		74	67399	54737
39	310652	295752		75	63410	50883
40	310445	295061		76	59654	47206
41	310811	294856		77	55881	43496
42	310953	294609		78	51757	39493
43	309982	293396		79	47490	35403
44	307815	290895		80	43389	31506
45	304502	287149		81	39413	27789
46	299105	281339		82	35323	24098
47	291882	273708		83	31137	20482
48	283624	265096		84	26965	17039
49	274263	255498		85+	108024	56589
50	264166	245277				

Reference: United Nations, Department of Economic and Social Affairs, Population Division (2024). World Population Prospects 2024, Online Edition. Available at: https://population.un.org/wpp/Download/Standard/Population/

Table S5. 15 year-old population entering the model by sex. [3]

Year	Females	Males	*Cont'd*	Year	Females	Males	*Cont'd*	Year	Females	Males
2011	421929	436232		2041	330202	344138		2071	254241	265732
2012	418940	433448		2042	326755	340588		2072	252595	264007
2013	415609	430303		2043	323317	336995		2073	249835	261135
2014	412687	427594		2044	320104	333674		2074	247317	258501
2015	409701	424818		2045	316932	330405		2075	244916	256017
2016	406549	421934		2046	313574	326866		2076	242361	253373
2017	404236	419740		2047	310184	323386		2077	240295	251198
2018	401976	417542		2048	306794	319949		2078	237881	248664
2019	398173	413683		2049	303643	316686		2079	235907	246592
2020	392627	407917		2050	301180	314145		2080	233923	244539
2021	385734	400919		2051	298936	311846		2081	231383	241900
2022	378120	393242		2052	295860	308666		2082	229430	239840
2023	370968	386113		2053	292882	305605		2083	227621	237948
2024	365679	380820		2054	290011	302661		2084	225952	236214
2025	362374	377372		2055	287480	300073		2085	224318	234517
2026	360440	375275		2056	285342	297905		2086	222743	232882
2027	359555	374206		2057	282732	295203		2087	221001	231056
2028	359095	373698		2058	279860	292217		2088	218806	228763
2029	358623	373254		2059	277208	289476		2089	216834	226704
2030	358500	373112		2060	275571	287772		2090	215320	225100
2031	358996	373546		2061	273969	286132		2091	213516	223219
2032	358695	373155		2062	272125	284236		2092	211882	221538
2033	357325	371791		2063	270361	282399		2093	210640	220239
2034	356544	371164		2064	268006	279988		2094	208801	218332
2035	354807	369592		2065	266319	278264		2095	207061	216521
2036	351338	366138		2066	264649	276548		2096	205741	215137
2037	346644	361325		2067	262034	273857		2097	204816	214176
2038	342152	356601		2068	259934	271668		2098	203553	212846
2039	338262	352529		2069	258118	269780		2099	201799	211012
2040	334057	348165		2070	255820	267395		2100	200192	209357

Reference: United Nations, Department of Economic and Social Affairs, Population Division (2024). World Population Prospects 2024, Online Edition. Available at https://population.un.org/wpp/Download/

Mortality projections from all causes by age, sex, and country

We calculated the mortality rates from all causes based on the UN projections specific to Colombia for the number of deaths and population by sex j and age m (μ_{jm}) from 2010 to 2100. [3] In Figure S2, we present the projected mortality rates by thousand population for selected ages (15 years old, 25 years old, 35 years old, 45 years old, 55 years old, 65 years old, 75 years old and 85 years old and more).

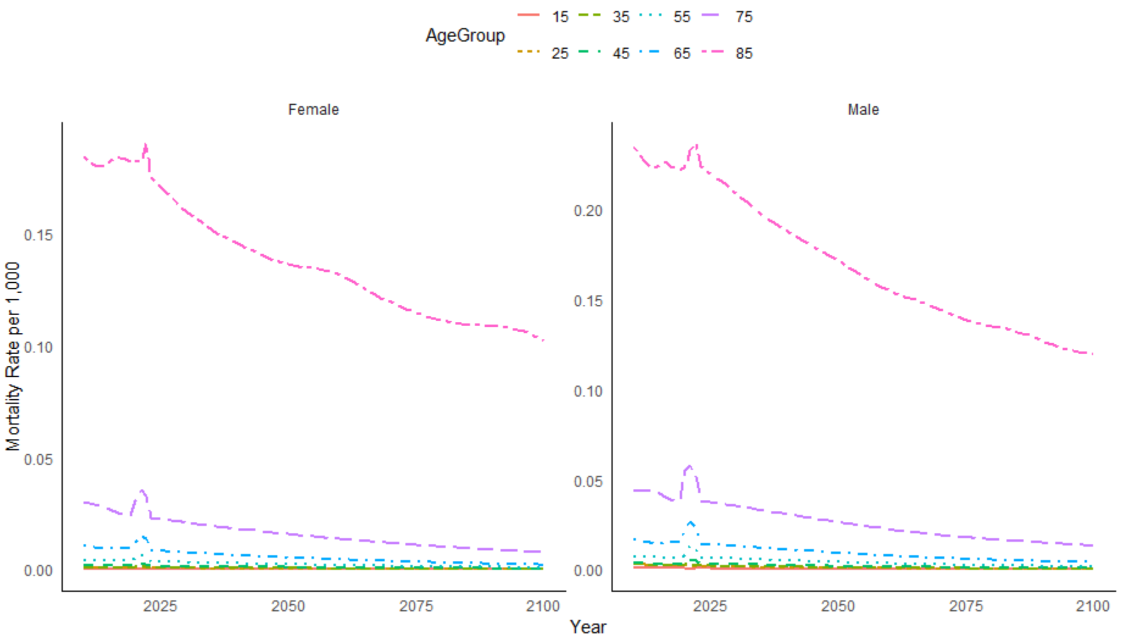

Figure S2. Mortality rates projections for 2010-2100 in Colombia.[a]

[a] The mortality rate calculations include the impact of the COVID pandemic, as registered deaths by age and sex available through 2021 were used. Reference: United Nations, Department of Economic and Social Affairs, Population Division (2022). World Population Prospects 2022, Online Edition. Available at https://population.un.org/wpp/Download/

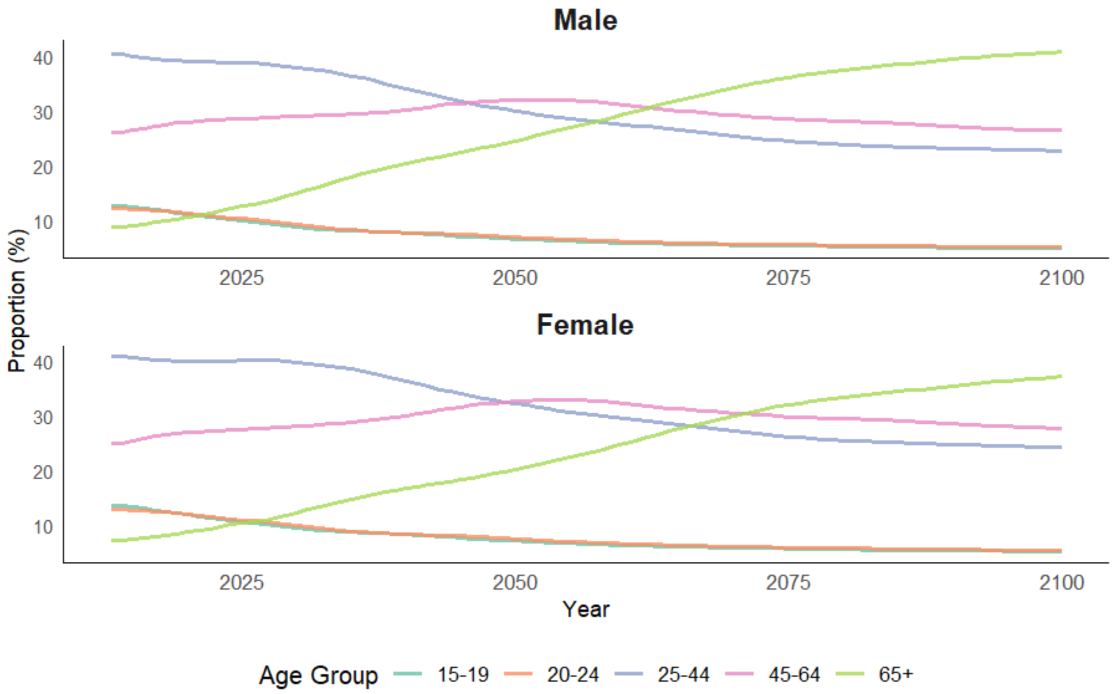

Figure S3. Age distribution of the simulated population over time by sex in Colombia, 2010 to 2100.

HPV natural history parameters

Here, we describe the parameters used in modeling the natural history of HPV (Table S6), previously introduced in Table S1. We calibrated all these parameters given the numerous challenges in accurately measuring such variables (obtaining precise estimates would require large cohorts of recently formed sexual partnerships along with frequent testing).

Probability of infection transmission per sex act with an infected person

Per-partnership infection transmission probability (β_j) was calibrated (see **Calibration** section) for each HPV type group using the lowest and highest values of the combined posterior density reported by Bogaards et al., as confidence intervals confidence for our calibration. [9]

Table S6. Model parameters values from the literature search used in the model.

Parameter	Symbol	Stratification	HPV type	Value (Range)	Calibrated	Reference
Demographic variables						
Mortality rate	$\mu_{j,m,t}$	Year, sex and age	All	Figure S2	No	United Nations [3]
Entries	$15yo_{j,t}$	Year and sex	All	Table S5	No	United Nations [3]
Natural history of HPV infection						
Probability of infection transmission per sex act with an infected person	β_j	None	16/18	0·87 (0·60 - 0·99)	Yes	Model based (POBASCAM) [9a]
			31/33/45/52/58	0·79 (0·30 - 0·99)		
			Non-vaccine	0·87 (0·38 - 0·99)		
Duration of infection (months)[b]	γ_j	Female	16/18	18 (14 - 22)	Yes	Placebo arm of FUTURE study[10c]
		Male		10 (6 - 20)		HIM study [11d]
		Female	31/33/45/52/58	18 (13 - 23)		Placebo arm of FUTURE study[10]
		Male		7 (6 - 18)		HIM study [11]
		Female	Non-vaccine	13 (12 - 16)		Placebo arm of FUTURE study[10]
		Male		8 (6 -18)		HIM study [11]
Probability of infections conferring natural immunity[e]	σ_j	Female	16/18	0·568 (0·348 – 0·754)	Yes	University students Washington [12]
		Male		0·05 (0·004 – 0·13)		Baseline from an RCT on HPV vaccine in men [13f]
		Female	31/33/45/52/58	0·33 (0·27-0·35)		Secondary analysis from HPV vaccine trial [14]
		Male		0·07 (0·04 - 0·11)		Baseline from an RCT on HPV vaccine in men[13]
		Female	Non-vaccine	0·35 (0·19 – 0·55)		National screening program in Slovenia [15g]
		Male		0·13 (0·02 - 0·61)		Seroconversion for HPV35 [16]
Duration of immunity	$1/\omega_j$	None	All	0·5 - 12 years	Yes	Assumed
Probability of HPV infections becoming latent	π	None	All	0·5 (0·1-0·9)	Yes	Assumed
Rate of reactivations of latent HPV infections	κ	None	All	0·17 (0·11-0·24)reactivareactiva	Yes	Ludwig-McGill cohort [17]
Sexual behavior and infection transmission						
Proportion of individual sexually active	$\tau_{j,m}$	Sex and age	All	Figure S4	No	National Survey of Family Growth (2017-2019) [18]
Rate of becoming sexually active	$\eta_{j,m}$	Sex and age	All	Figure S5	No	National Survey of Family Growth (2017-2019) [18]
Proportion of individuals in sexual activity level l	$\theta_{j,l,m}$	Sex, sexual activity level and age	All	Figure S6	No	National Survey of Family Growth (2017-2019) [18h]
Probability of changing sexual activity level	$\phi_{j,m}$	Sex and age	All	Figure S7	No	National Survey of Family Growth (2017-2019) [18]
New partner acquisition rate	$c_{j,l,m}$	Sex, sexual activity level and age	All	Figure S8	Yes	National Survey of Family Growth (2017-2019) [18]

Parameter	Symbol	Stratification	HPV type	Value (Range)	Calibrated	Reference
Proportion of sexual partnerships made exclusively with same sexual activity level	ε_{sex}	None	All	0·2 (0·004 - 0·779)	Yes	Assumed [19-21,i]
Proportion of sexual partnerships made exclusively with same age group	$\varepsilon_{age,j}$	Female	All	0·41 (0·1 - 0·84)	Yes	National Survey of Family Growth (2017-2019) [18,j,k]
		Male	All	0·48 (0·1 - 0·99)		
Compromise between females' and males' desires for sexual partnerships	υ	Sex		0·5[l]	No	Assumed

hrHPV: high risk human papillomavirus. HPV: human papillomavirus, RCT: randomized controlled trial, NA: not applicable, WL: females in low sexual activity level, WH: females in high sexual activity level ML: males in low sexual activity level, MH: males in high sexual activity level, SD: standard deviation.

[a] Model-based estimation using data from two large-scale surveys on sexual behavior and a randomized controlled trial (POBASCAM) in the Netherlands. The value is the averaged over-all carcinogenic HPV types, and over HPV-16/18. Range corresponds to the posterior density; we selected the lowest and highest values among the individual HPV types included.

[b] Assuming the average value for HPV35 and 59 represent the entire non-vaccine high risk HPV group in females. For males, we used the average of HPV 35, 39, 51, 56, 59 and 66. Clearance rate was defined as one over duration of infection.

[c] Average of the mean durations of infection considering the Natural History perspective.

[d] Weighted average for median time to clearance between HPV 16 and HPV 18 (12·2*91) + (6·3*44) / (91+44) = 10·28 months equal to 0·86 years

[e] Correspond to the average between HPV types incident infections that seroconverted.

[f] Seroprevalence at baseline in heterosexual males for concordant HPV type.

[g] Correspond to the number of females who seroconverted for HPV 35, 39, 56 and 59 (three-year follow-up).

[h] Based on the proportion of Hispanic respondents who had ≤2 and >2 partners in the last 12 months during 2017-2019, among those who reported ever having sex.

[i] Calibrated for a mean of 0·2 and a 95% confidence interval of 0·064-0·425 to cover plausible values reported in the literature. [19-21]

[j] The standard deviation was high producing values outside of the possible range (0 to 1); therefore, we truncated the range when necessary.

[k] Newman's coefficient calculated considering five age groups: 15-19 years old, 20-24 years old, 25-44 years old and 45+ years old.

[l] A value of 1 means woman chooses; a value of 0 means man chooses; and any value between 0 and 1 means a compromise between both desires.

[m] Calibrated for a mean of 0·2 and a 95%Confidence Interval of 0·064 to 0·425 to cover plausible values reported in the literature (Malagon et al and Garnet et al)

Loss of HPV positivity rate

Infected individuals can become HPV negative at a rate (γ_j). We defined loss of positivity rate as the inverse of the duration of HPV DNA positivity, which varied by sex. Insinga et al., used data from the placebo arm of randomized clinical trials for the quadrivalent vaccine in females. We calculated the average of the mean duration for our HPV type groups considering the natural perspective (time until treatment for cervical intraepithelial neoplasia 3+) and selected the lowest and highest value of the confidence interval for our range in the calibration (see **Calibration** section). [10] In the case of males, we used the weighted average for median time to loss of positivity from the HIM study for the different HPV type groups; for example, in the case of HPV16 and HPV18, the value was (12·2*91) + (6·3*44) / (91+44) = 10·28 months. For our calibration range, we used the lowest and highest values of the confidence intervals for the group. [11] As the infection's duration was not reported for some of the HPV groups, we used the average value for HPV35 and HPV59 as a proxy for other non-vaccine carcinogenic HPV in females, and the average of HPVs 35, 39, 51, 56, 59 and 68 as a proxy for other non-vaccine carcinogenic HPV in males. We assumed that individuals who did not develop natural immunity or enter latency would clear the infection.

In the model, persistent HPV infection is not represented as a separate health state but arises implicitly from the balance between acquisition, clearance, latency, and reactivation, consistent with epidemiological definitions of persistence based on repeated detection over time rather than continuous detectable infection.

Probability of infections conferring natural immunity

The probability of developing natural immunity was different for females and males (σ_j) and by group of HPV type. For females, we averaged the values for HPVs 16 and 18 reported by Carter et al., in a cohort of 18-20 years-old university students using antibody seroconversion following incident infection; the ranges that we used in the calibration (see **Calibration** section) included the lowest and highest values of their confidence intervals. [12] In the case of HPVs 31, 33, 45, 52, and 58, we used the average reported by Brown et al. [14] And for non-vaccine carcinogenic HPV types, we used the values reported by Artemchuk et al. [15] For males, we averaged the seroprevalence values for concordant HPV types at baseline reported by Tota et al., [13] in 16-23 years-old heterosexual males enrolled in a randomized clinical trial for the quadrivalent vaccine, for HPVs 16 and 18 as well as for HPVs 31, 33, 45, 52, and 58; for the ranges, we also selected the lowest and highest values of their confidence intervals. For non-vaccine carcinogenic HPV types, we used the HPV35 seroconversion rate reported by Edelstein et al. [16]

Waning rate of natural immunity

We assumed that natural immunity would wane with time. The rate of waning of natural immunity (ω) was calculated as one over the duration of natural immunity. To estimate the duration of natural immunity, we used antibody seropositivity as a proxy of immunity and assumed seropositivity wanes at a constant rate. Wang et al., showed that 45% of females lose their HPV seropositivity (and 55% of initially seropositive females at baseline remain seropositive) after 6.4 years, which corresponds roughly to an average duration of immunity of 10 years, assuming a constant rate of immunity decay (equation 22). [22] Considering the scarce literature, we decided to calibrate the duration of immunity using a uniform distribution from 0.5 to 12 years (see **Calibration** section). This range includes the calculated mean duration of 10 years but also considers the possibility of shorter and longer durations.

$$\frac{1}{\omega} = \frac{6\cdot 4}{\ln\left(\frac{1}{0\cdot 55}\right)} = 10 \cdot 7 \qquad ; (22)$$

Probability of HPV infections becoming latent

To our knowledge, there is no available information about the probability of infections becoming latent (π). Therefore, we decided to calibrate this value with a mean of 0·5 and a range from 0·1 to 0·9 (see **Calibration** section).

Rate of reactivation

For the rate of reactivation of latent infections (κ), we based our priors on the cumulative incidence of additional redetections (third or more detections) of the same HPV genotype in females from the Ludwig-McGill cohort, one year after loss of positivity of the previous detection (15·8%). [17] We assumed the rate of reactivation would not vary by sex or age or HPV type (equation 23).

$$\kappa = -\ln(1 - 0 \cdot 158) = 0 \cdot 17 \qquad ; (23)$$

Vaccination parameters

We have considered two parameters related to HPV vaccination: vaccination coverage by sex (α_j) and 1 - vaccine efficacy (χ_j). For the calibration process, we assumed that the population was unvaccinated as prevalence reported in

literature corresponds to HPV prevalence prior to vaccination. The vaccine efficacy against placebo for the quadrivalent vaccine was 98% in girls for three doses on a composite outcome (cervical intraepithelial neoplasia grade 2+ with the detection of HPV 16 and 18 DNA in one or more of three adjacent sections of the same lesion); [23] and 87·4% in boys for persistent infection of HPVs 16 and 18. [24] The nonavalent vaccine has shown noninferiority to the quadrivalent vaccine in the prevention of persistent infection and precancerous lesions related to HPV16. We assumed it would be the same in the case of boys. For HPVs 31, 33, 45, 52, and 58, we used the 96·7% efficacy reported against cervical disease of any grade in females and the 93·8% efficacy against persistent infection in boys. We also assumed lifelong vaccine protection and non inferiority of one dose to two doses. [25,26]

Sexual behavior parameters

Demographic surveys carried out in Colombia and other South American countries do not provide sufficient detail to calculate the necessary sexual behavior parameters. Therefore, we used data from the population of Hispanic origin from the 2017-2019 National Survey on Family Growth (NSFG) in the United States of America to estimate several parameters related to sexual behavior. [18] The NSFG is a survey developed by the Centers for Disease Control and Prevention that collects information on family life as well as general and reproductive health including variables such as sexual activity, the number of lifetime opposite-sex partners, and the number of opposite-sex partners in the last year, which were relevant for our analysis. We used the population of Hispanic origin (1,705 females and 1,356 males) as a proxy for the population in South American countries. When data was available, we validated the values with reports from South American countries. Below, we describe the calculations for each parameter using the NSFG. Some sexual behavior parameters (new partner acquisition rate and assortativeness coefficients) were later calibrated to ensure that the sexual behavior parameter values reproduced HPV infection epidemiology in the modeled countries (see **Calibration** section).

Proportion of sexually active individuals

We calculated the proportion ever having intercourse (either vaginal, oral, or anal intercourse) with someone of the opposite sex (variable oppsexany) for ages 15 to 24 years and for 25 years or more with in the NSFG (Figure S4). [18] We chose a cutoff of 25 years or older because, after this age, the differences in the proportion of sexually active individuals from one age to the other were minimal. Due to the small sample size of Hispanic respondents in the survey, there was some instability in estimates across ages, so we used smoothing techniques to ensure an increasing proportion of sexually active individuals with age. When the proportion of sexually active individuals at a given age was lower than that of the previous age, we replaced it with a 3-year smoothed average (including the previous and next age values). For 19-year-old males, we used the average of only the previous and next year. We used the survey weights provided by the National Center for Health Statistics to calculate the proportions. We validated our estimates against local surveys; we obtained similar proportions of sexually active individuals to those reported in Bolivia, Colombia, and Peru for 15 years old; [27] as well as in Argentina [28] for 16 to 17 years old. The proportion of individuals sexually active was used to assign the initial population proportion of not sexually active, and to calculate the rate of becoming sexually active as individuals age in the model (see **Rate of becoming sexually active** section below).

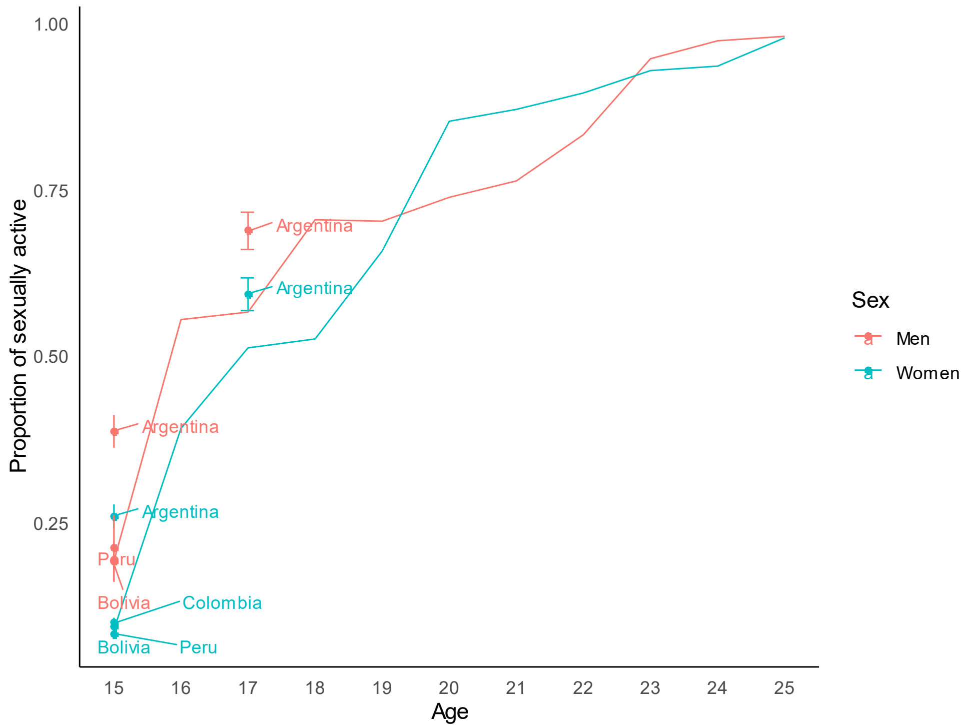

Figure S4: Proportion of individuals sexually active by age and sex. [18,27,28]

Rate of becoming sexually active

We based the rate of becoming sexually active by sex and age (η_{jm}) on reported proportions of individuals sexually active by age calculated from the NSFG above.[18] We used a discrete approximation of the instantaneous rate of change for a proportion over time (equation 24).

$$\frac{p_{t1}}{p_{t0}} = exp^{-rate*(t1-t0)}$$

$$\eta = -\frac{ln\frac{1-p_{t1}}{1-p_{t0}}}{t1-t0} \quad ; \qquad ; (24)$$

where p is the proportion of sexually active Hispanic respondents and t the ages, e.g., if 39% of 16-years-old females and 9% of 15-years-old females are sexually active, the rate for females becoming sexually active at 16 years is equal to:

$$-\frac{ln\frac{(1-0\cdot39)}{(1-0\cdot09)}}{16-15} = 0 \cdot 40\text{/per person-year}$$

We calculated the rate yearly for individuals between ages 16 and 24, and then we used a single rate for individuals aged 25 and older (Figure S5).

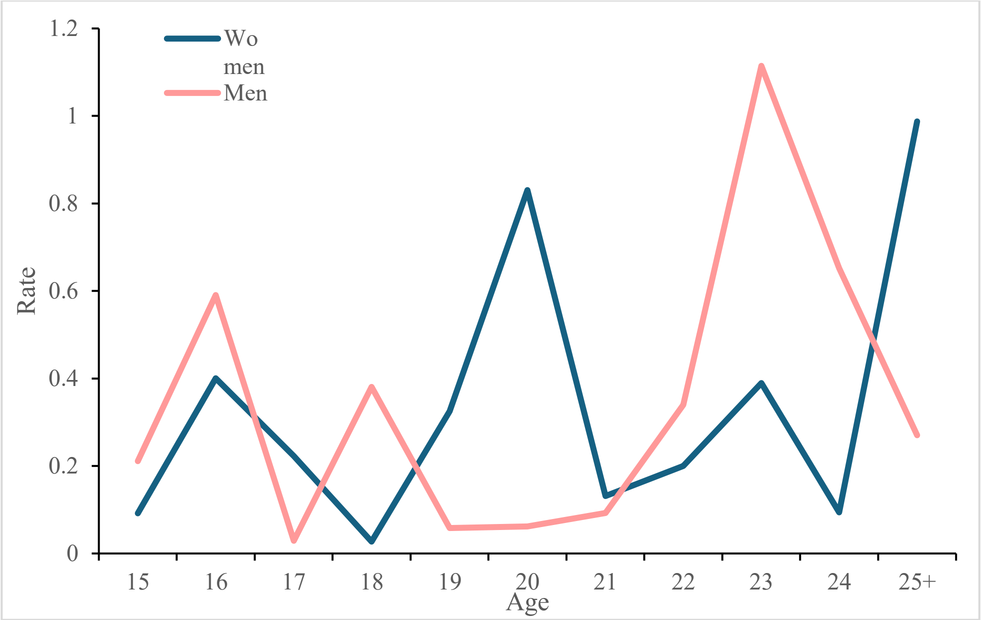

Figure S5. Rate of becoming sexually active per year by age and sex. [18]
Reference: National Center for Health Statistics. Centers for Disease Control and Prevention. NSFG - National Survey of Family Growth Homepage. 2023. Available from: https://www.cdc.gov/nchs/nsfg/index.htm

Sexual activity level

We defined the proportions in each sexual activity level (θ_{jlm}) based on the number of sexual partners of the opposite sex reported in the last 12 months among those who reported having sex in the last year in the NSFG (variable oppyearnum). [18] We used a cutoff of two or fewer sexual partners of the opposite sex to define the "Low" sexual activity level and three or more for the "High" sexual activity level. We calculated the proportion of individuals in each sexual activity level by age group (15-19 years old, 20-24 years old, 25-44 years old and 45+ years old); these proportions were assigned for the initial population and for the entries. In Figure S6, we show the proportions by sex and age groups.

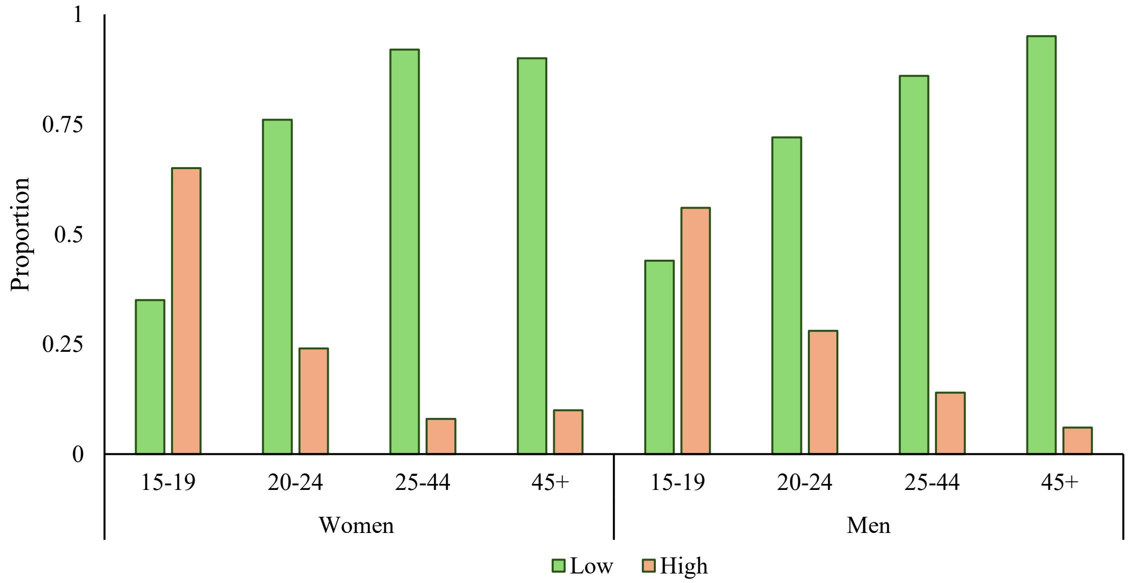

Figure S6. Proportion of individuals by sexual activity level, age group and sex. [18]

Low sexual activity level was defined as two or less opposite sexual partners in the last year, and high sexual activity level as more than two sexual partners in the last year. Reference: National Center for Health Statistics. Centers for Disease Control and Prevention. NSFG - National Survey of Family Growth Homepage. 2023. Available from: https://www.cdc.gov/nchs/nsfg/index.htm

Probability of changing sexual activity level

We assumed our population could change (ϕ_{jm}) sexual activity levels over time (at 20, 25, and 45 years of age, as proportions were calculated for these age groups). We calculated the probability of moving from the high to the low sexual activity level between age group m-1 and m as the fractional decrease in the proportion of individual in the high activity level (equation 25). In cases where the proportion in the high activity level increased (e.g., females aged 45 years), this approach produced a negative probability. When applied in the model, such negative probabilities resulted in an addition of individuals to the high activity level, maintaining consistency with the observed data.

$$\phi_{jl=high\rightarrow low,m} = \frac{\theta_{j,l=high,m-1} - \theta_{j,l=high,m}}{\theta_{j,l=high,m-1}} \quad ; (25)$$

New partner acquisition rate

Due to decreasing partner acquisition with age, we assumed a log-linear relationship between age and partner acquisition rate to fit new partner acquisition rates (c_{jlm}). First, we created a centered log-age variable defined as ln(AGE_R – 13). The subtraction of 13 ensures that an individual aged 14 yields a ln(1) = 0, so with a regression without intercept the predicted number of lifetime partners at age 14 is zero. We then fitted a linear regression (no intercept) of lifetime opposite-sex partners on the centered log-age, stratified by sex and sexual activity level (equation 26). [18] With these coefficients, we calculated the predicted number of lifetime sexual partners by age, sex and sexual activity level, and compared it to the observed values from the population of Hispanic origin from the 2017-2019 NSFG (Figure S7). As there was good model fit, we used the fitted coefficient in equation 26 to obtain the new partner acquisition rates (equation 27):

$$\text{Lifetime sexual partners}_{jl} = \psi_{jl} * \ln(AGE_R - 13); (26)$$

$$c_{jlm} = ln\left(\frac{m}{m-1}\right) * \psi_{jl} \quad ; (27)$$

For males in the age groups 15 to 19 and 20 to 24 in the low sexual activity level, the predicted values in the linear-log model overestimated the observed number of lifetime sex partners (Figure S7). Hence, we decided for these males to use a spline regression of the number of lifetime sexual partners over age restricted to the low sexual activity level, with a spline for the 15 to 19 age group and another for the 20 to 24 age group. For the rest (25+ year-old males), we used the coefficient from the linear-log model. Figure S8 shows the new partner acquisition rate by sex, sexual activity level, and age following these fitting procedures (c_{jlm}). The coefficients and their fitted confidence intervals were used as priors for calibration (see **Calibration** section).

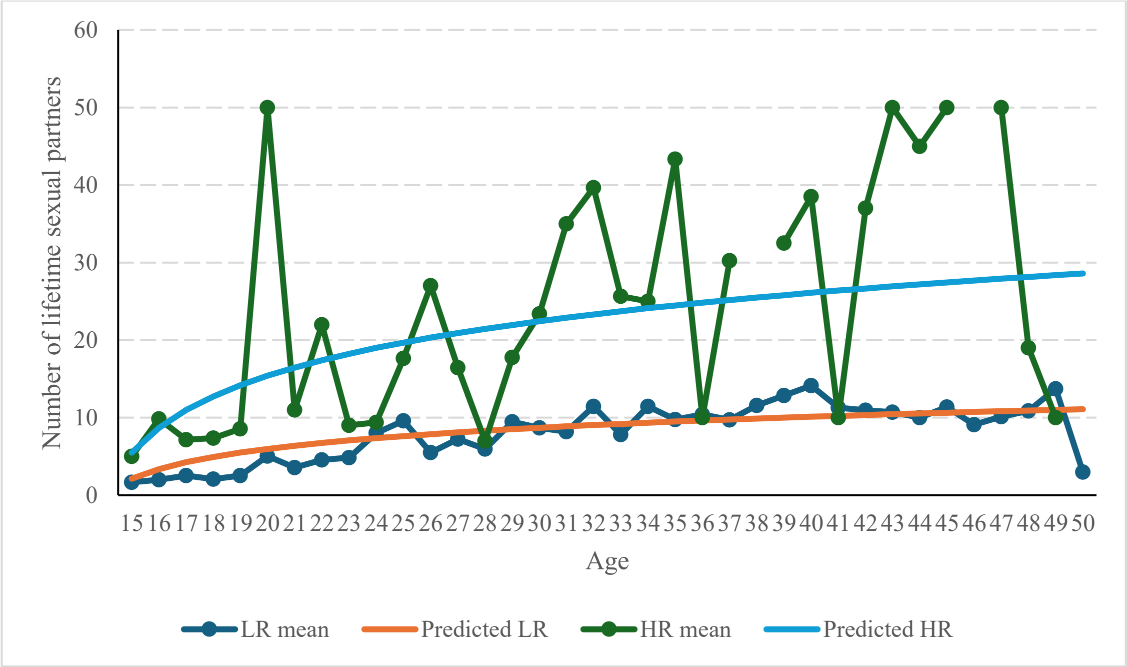

(A)

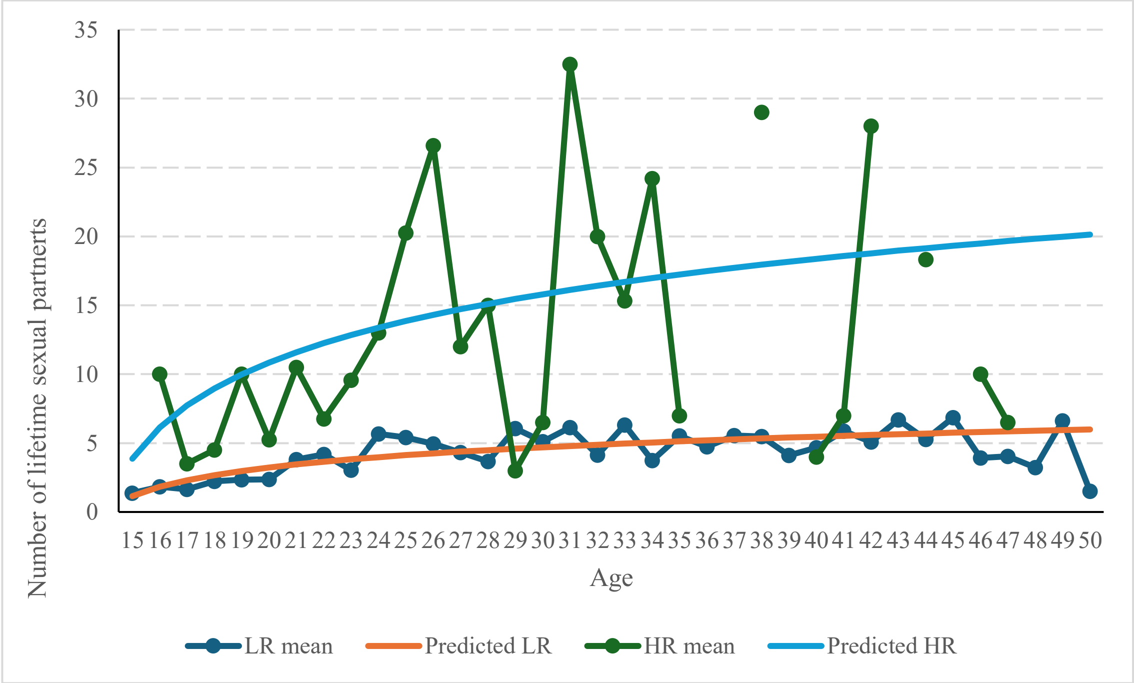

(B)

Figure S7. Observed vs predicted number of lifetime sexual partners in low and high sex activity level Hispanic males (A) and females (B).

LR: low risk; HR: high risk. The variable “number of lifetime sexual partners of the opposite sex” was truncated at 50 (e.g., the value 50 represents 50 or more partners) in the NSFG survey. Reference: National Center for Health Statistics. Centers for Disease Control and Prevention. NSFG - National Survey of Family Growth Homepage. 2023. Available from: https://www.cdc.gov/nchs/nsfg/index.htm

Newman's coefficient for assortativity by age and sex

To estimate the value of assortativity parameter, we calculated the Newman's coefficient[29] for assortativity by sexual activity level (ε_{sex}) and age ($\varepsilon_{j\text{-}age}$) considering five age groups: 15-19 years old, 20-24 years old, 25-44 years old and 45+ years old (equation 26).

$$\varepsilon = \frac{\sum_i e_{ii} - \sum_i a_i b_i}{1 - \sum_i a_i b_i} \qquad ; (26)$$

where e is a matrix, whose element are e_{ij}; e_{ii} represents a cell where both individuals had the same sexual activity level or same age, a_i the sum of the rows and b_i the sum of the columns.

We calculated the assortativity parameter by age in each sex ($\varepsilon_{age,j}$) using the NSFG data. [18] The NSFG has different sets depending on the sex of the respondent, and hence, the variables varied across datasets. In the case of females, we first categorized the respondent's age at first sexual intercourse with the last partner (P1YRAGE) and the reported last partner's age at the time they first had sexual intercourse (P1YHSAGE) into age groups, as previously mentioned. With these variables, we constructed the two-by-two tables necessary for the calculations. For males, we used the respondent's current age (AGE_R) and the reported current age of their partner (cwpage) to construct the matrix. We used the Jacknife method to calculate the standard deviation. The assortativity parameter by age in females is 0·41 (SD: 0·20) and 0·48 (SD: 0·32) in males. We used these values as priors for calibration (see **Calibration** section). We were not able to calculate the assortativity parameter by sexual activity level using data from the NSFG; hence we decided to calibrate it based on several reports from the literature, [19-21] considering a mean value of 0·2 with a confidence interval between 0·004 and 0·779 (see **Calibration** section) for both males and females.

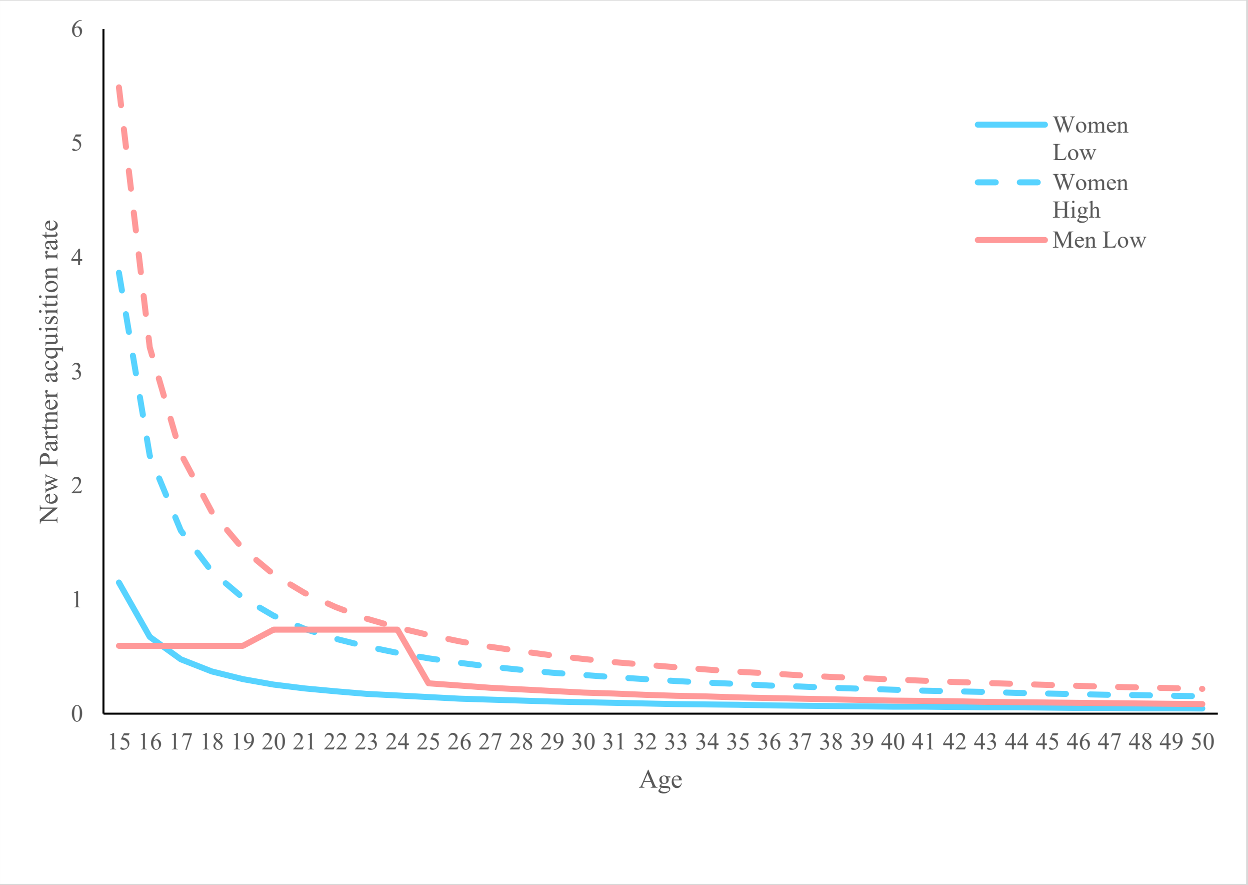

Figure S8. New partner acquisition rate among sexually active individuals by sexual activity level and age. Reference: National Center for Health Statistics. Centers for Disease Control and Prevention. NSFG - National Survey of Family Growth Homepage. 2023. Available from: https://www.cdc.gov/nchs/nsfg/index.htm

CALIBRATION

Calibration is an important step in mathematical modeling to estimate parameter values that are not well characterized in the literature and therefore carry greater uncertainty. The main objective is to identify parameter sets that best reproduce the observed data. We first ran a burn-in simulation of 90 years to attain a stable distribution of states in the population so as to use these as initial state value inputs. Secondly, we carried out the calibration in two steps. We first calibrated sexual behavior parameters and HPVs 16 and 18 natural history parameters based on their prevalence. Then, we calibrated natural history parameters for the remaining HPV type groups using the sexual behavior parameters obtained in the previous step. We describe below each process in detail.

Prevalence studies

We used the prevalence in females from Colombia reported by Molano et al., [30] del Rio et al., [31] Puerto et al., [32] Leon et al., [33] Muñoz et al., [34] Velicer et al., [35] and the ESTAMPA study. [36,37] As we only found one study reporting prevalence in males in Colombia[38] we also considered the results from Sudenga et al., [39] from Mexico as a proxy (Table S7).

Burn-in simulation to obtain initial state values

We started the model with the initial parameters (see **Model variables and parameters** section) and an infected population based on the carcinogenic HPV prevalence in females by age groups (15 to 29 years old, 30 to 34 years old, 35 to 39 years old, 40 to 44 years old, and 45 or more years old) reported by Molano et al., [30] in Colombia. Because there are no actual data for the proportion of the population in the latent and recovered states, we started the burn-in with a hypothetical proportion of 10% each.

We used the proportions of the population distribution in each stratum after 90 years to generate the initial state values for the calibration process. We considered that, at this point, an equilibrium was achieved, as there were only minimal changes in HPV prevalence for all ages after year 50.

Latin hypercube sampling

We generated 10,000 parameters sets for calibration using Latin Hypercube Sampling. [40] Sexual behavior parameters included in the calibration were: new partner acquisition rate, assortativity parameter by age in females and in males, and assortativity parameter by sexual activity level. Natural history parameters included in the calibration were: probability of infection transmission (β_j), proportion of infections conferring natural immunity (σ_j), loss of positivity rate (γ_j), waning rate of natural immunity (ω_j), probability of becoming latent (π), and reactivation rate (κ).

In the case of the new partner acquisition rate, we sampled the regression coefficients, that were used to calculate the rates, using normal or normal truncated distribution when the lower range bound was negative. The standard deviations of the assortativity parameters were high. To address this issue, we calculated the 95% confidence interval for females and truncated the lower bound to 0·01. For males, we used the calculated standard deviation and truncated the values to be between 0·01 and 0·99. Regarding the infection clearance rate, we calibrated the time to clearance (i.e., the duration of infection). Similarly, for the waning rate of natural immunity, we calibrated the duration of immunity (i.e., protection time). With this value we calculated the rates as described above (se **Natural history parameters** section).

Sexual behaviour parameters were calibrated using HPV16/18 data together with natural history parameters, resulting in 50 parameter sets for that group. For the subsequent calibrations of the remaining two HPV groups, the sexual behaviour parameter values were drawn from the 50 preselected sets. Each of the 50 sets was used an equal number of times across 10,000 priors (i.e., 200 repetitions per set). Minor differences in posterior sexual behaviour parameters across groups of HPV types reflect the sampling procedure rather than biological differences and should be interpreted accordingly. The distributions, as well as the prior and posterior means, and ranges for each parameter and HPV type group are shown in Table S8.

Table S7. Prevalence parameters used for calibration.

Author (Year)	Age range	HPV type	Value
Del Río (2016) [31]	15-19	16/18	0·214
		31/33/45/52/58	0·087
ESTAMPA (2022) [36,37]	30-64	16/18	0·033
		31/33/45/52/58	0·039
		Non-vaccine	0·052
Leon (2009) [33]	30-50	16/18	0·039
		31/33/45/52/58	0·063
Molano (2002) [30]	25-34	16/18	0·032
		31/33/45/52/58	0·018
		Non-vaccine	0·016
	35-44	16/18	0·019
		31/33/45/52/58	0·010
		Non-vaccine	0·009
Muñoz (1996) [34]	30-50	16/18	0·055
Puerto (2018) [32]	18-25	16/18	0·144
		31/33/45/52/58	0·163
		Non-vaccine	0·215
Velicer (2009) [35]	30-50	16/18	0·066
Castellsagué (1997) [38]	30-50	16/18	0·016
		31/33/45/52/58	0·011
		Non-vaccine	0·021
Sudenga (2017) [39]	18-44	16/18	0·070
		31/33/45/52/58	0·093
		Non-vaccine	0·197

Table S8. Parameters distributions mean and range used in the Latin Hypercube Sampling and in selected sets.

Parameter	Distribution	HPV type	Priors mean	Priors range	Posteriors mean	Posteriors range
Sexual behavior parameters						
Epsilon coefficient for age in females	Inverse logit transformation of normal	16/18	0·41	0·01-0·84	0·33	0·05-0·68
		31/33/45/52/58			0·33	0·05-0·47
		Non-vaccine cHPV			0·33	0·08-0·76
Epsilon coefficient for age in males	Normal truncated	16/18	0·48	0·32 [a]	0·27	0·02-0·76
		31/33/45/52/58			0·09	0·02-0·56
		Non-vaccine cHPV			0·50	0·13-0·68
Epsilon coefficient for sex [b]	Beta	16/18	0·2	0·01-0·4	0·23	0·06-0·63
		31/33/45/52/58			0·22	0·07-0·63
		Non-vaccine cHPV			0·16	0·06-0·57
Coefficients for new partner acquisition rate						
Females low sexual activity level	Normal	16/18	1·66	1·55-1·77	1·65	1·52-1·75
		31/33/45/52/58			1·64	1·59-1·72
		Non-vaccine cHPV			1·66	1·58-1·75
Females high sexual activity level	Normal	16/18	5·58	4·53-6·62	5·65	4·51-7·50
		31/33/45/52/58			5·49	4·51-6·66
		Non-vaccine cHPV			5·51	4·80-6·66
Males low sexual activity level age 15 to 19	Normal	16/18	0·59	0·49-0·70	0·60	0·50-0·69
		31/33/45/52/58			0·60	0·52-0·69
		Non-vaccine cHPV			0·59	0·50-0·64
Males low sexual activity level age 20 to 24	Normal truncated	16/18	0·74	0-1·64	0·73	0·08-1·56
		31/33/45/52/58			0·73	0·08-1·10
		Non-vaccine cHPV			0·97	0·22-1·56
Males low sexual activity level age 25+	Normal	16/18	3·07	2·85-3·29	3·08	2·82-3·40
		31/33/45/52/58			3·13	2·82-3·40
		Non-vaccine cHPV			3·08	3·01-3·28
Males high sexual activity level	Normal	16/18	7·91	7·01-8·83	7·90	7·11-9·52
		31/33/45/52/58			7·91	7·11-8·70
		Non-vaccine cHPV			7·78	7·18-8·74
Natural history parameters						
Probability of infection transmission	Inverse logit transformation of normal	16/18	0·865	0·39-0·99	0·62	0·25-0·93
		31/33/45/52/58	0·800	0·39-0·99	0·77	0·25-0·99
		Non-vaccine cHPV	0·870	0·38-0·99	0·84	0·50-0·99
Proportion of infection conferring natural immunity in females	Inverse logit transformation of normal	16/18	0·568	0·348-0·754	0·58	0·39-0·72
		31/33/45/52/58	0·332	0·273 -0·354	0·33	0·28-0·36
		Non-vaccine cHPV	0·3548	0·1923-0·5463	0·25	0·15-0·41
Proportion of infection conferring natural immunity in males	Inverse logit transformation of normal	16/18	0·049	0·0038-0·127	0·06	0·01-0·18
		31/33/45/52/58	0·068	0·032-0·108	0·07	0·04-0·09
		Non-vaccine cHPV	0·125	0·019-0·613	0·13	0·02-0·40
Duration of infection in females (months)	Log normal	16/18	18·15	13·9-22	17·63	12·36-22·45
		31/33/45/52/58	17·68	13-22·5	19·36	15·11-25·53
		Non-vaccine cHPV	13·4	11·6-16	13·22	10·77-15·46
Duration of infection in males (months)	Log normal	16/18	10·28	6-20·2	8·99	4·78-16·40
		31/33/45/52/58	7·05	6-18·2	7·84	4·51-16·48
		Non-vaccine cHPV	7·8	6-18·4	8·50	4·97-13·91

Duration of immunity (years)	Uniform	16/18	NA	0·5-12	7·13	0·96-11·83
		31/33/45/52/58			4·57	0·52-12·00
		Non-vaccine cHPV			7·69	1·16-11·92
Proportion of infection becoming latent	Beta	16/18	0·5	0·1-0·9	0·87	0·67-0·99
		31/33/45/52/58			0·40	0·07-0·81
		Non-vaccine cHPV			0·03	0·001-0·10
Reactivation rate of a latent infection	Inverse logit transformation of normal	16/18	0·17	0·11-0·24	0·15	0·11-0·22
		31/33/45/52/58			0·17	0·12-0·25
		Non-vaccine cHPV			0·16	0·11-0·25

cHPV: carcinogenic HPV.

[a] The value corresponds to the standard deviation.

[b] We tested different alpha and beta parameters until we obtained a p50 of 0·2 and p95 of 0·4.

Selection of best fitting sets

When assessing the HPV prevalence reported from different studies, we found important heterogeneity across studies and, in some cases, non-overlapping confidence intervals for the same age group. Studies may have selection and measurement bias that can explain the different results. Hence, we decided to take a hybrid approach for the selection of the best 50 parameters sets for each HPV type group and used larger confidence intervals for calibration targets to account for uncertainty related to heterogeneity and bias across studies.

In the first stage, we ran the 10,000 parameters sets for 12 years and selected those sets that fell within the confidence intervals of HPV prevalence in younger cohorts of females (15 to 24 years old) based on prevalence data reported by Del Rio-Ospina et al.,[31] and Puerto et al.[32] We doubled the size of the reported confidence intervals to integrate higher uncertainty in HPV prevalence due to heterogeneity across studies, and to capture more sets in the first step. In the case of males, as only one HPV prevalence study was available for Colombia, we additionally used data from Sudenga et al.,[39] conducted in Mexico. For some prevalence targets, we restricted the age range to reflect the age distribution of the underlying study population. For example, Sudenga et al. reported that 87% of male participants were aged 18–44 years; therefore, we used this age range as the calibration target rather than the full reported age range (18–70 years).

Secondly, we ran the model with parameter sets selected in the first stage to select those sets that fall within the increased confidence interval (similarly to the previous step) of HPV prevalence in 25 to 64 years old females. Since running a simulation over the lifetime of a cohort takes significant time, our two steps approach improved computing time by allowing us to run a more restricted number of parameters sets to identify those that also fitted prevalence in older females during the second step.

Finally, among these parameters sets, we selected the 50 sets with the highest log-likelihood as a goodness of fit measure to assess the agreement between estimated and observed data (e.g., the log-likelihood that the observed data was generated by the parameter set) for all HPV prevalence studies in both females and males (Table S8). Figures S9 to S11 show the prevalence by age in females and males from the 50 selected sets in each HPV type group compared to the reported prevalence from the literature.

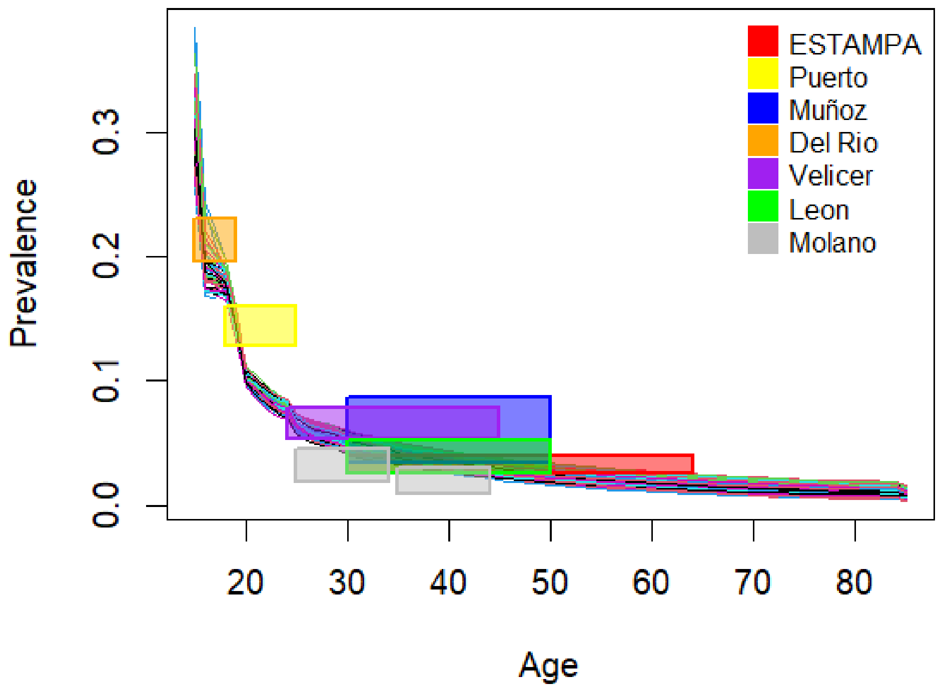

A

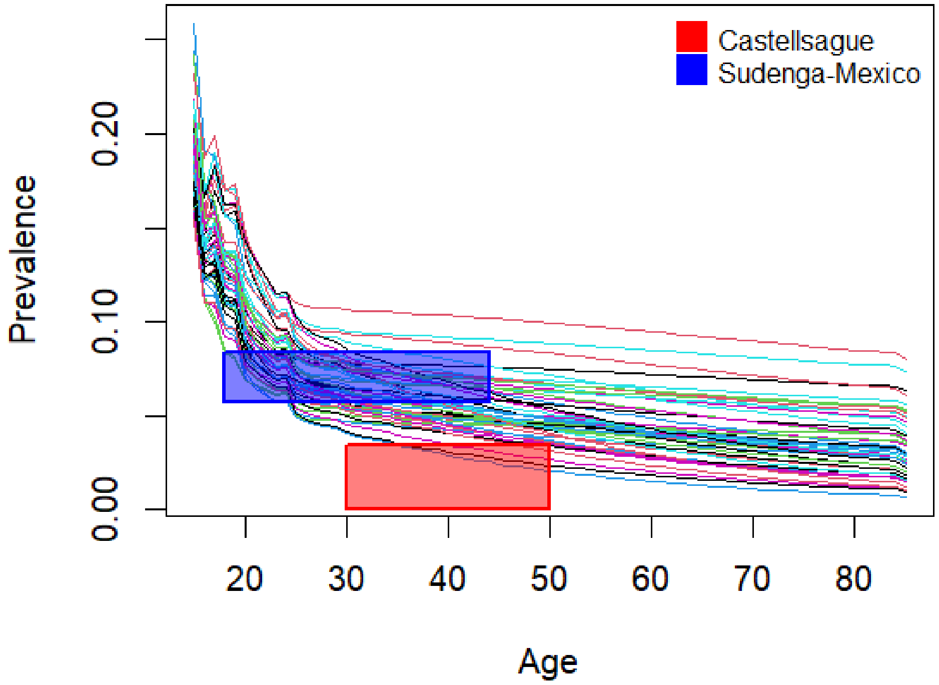

B

Figure S9. Age-specific prevalence of HPV-16 and HPV-18 in females (A) [31-37] and males (B). [38,39]
Note: Model-simulated results from selected sets (lines) compared with reported prevalence from the literature (boxes). The boxes represent the age range reported in studies (length) and the confidence interval (height) of the reported prevalence in the studies.

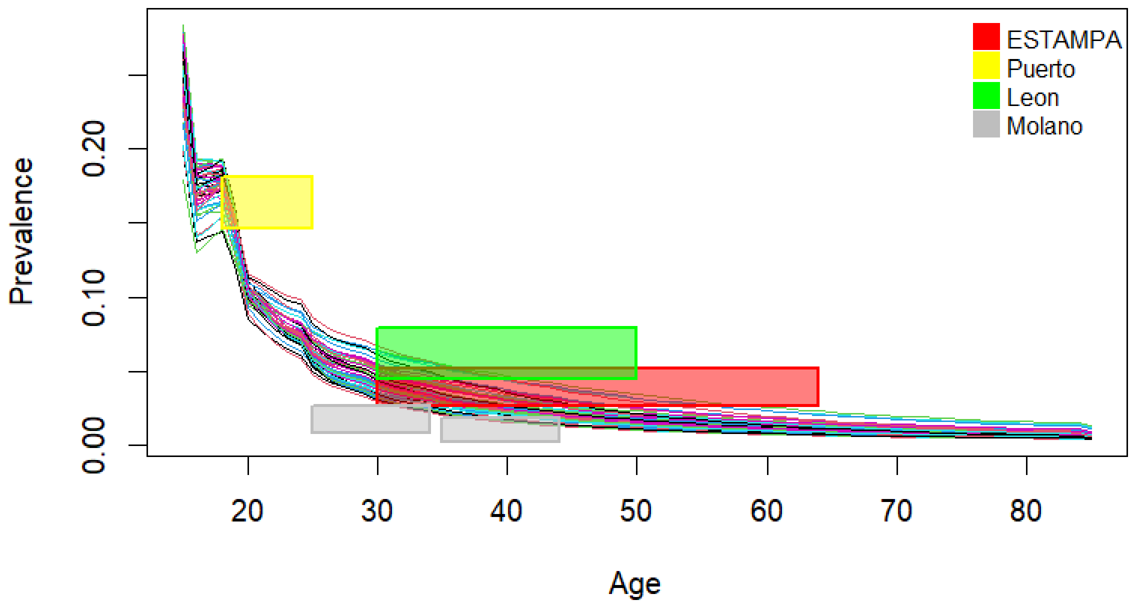

A

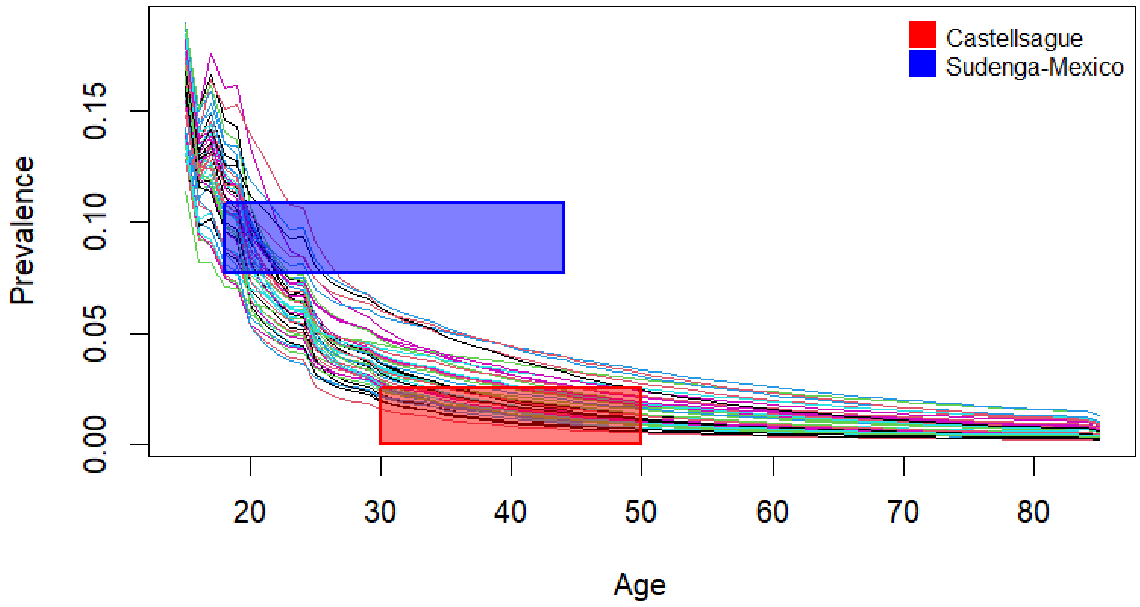

B

Figure S10. Age-specific prevalence of HPV 31/33/45/52/58 in females (A) [30-33,37] and males (B). [38,39]

Note: Model-simulated results from selected sets (lines) compared with reported prevalence from the literature (boxes). The boxes represent the age range reported in studies (length) and the confidence interval (height) of the reported prevalence in the studies.

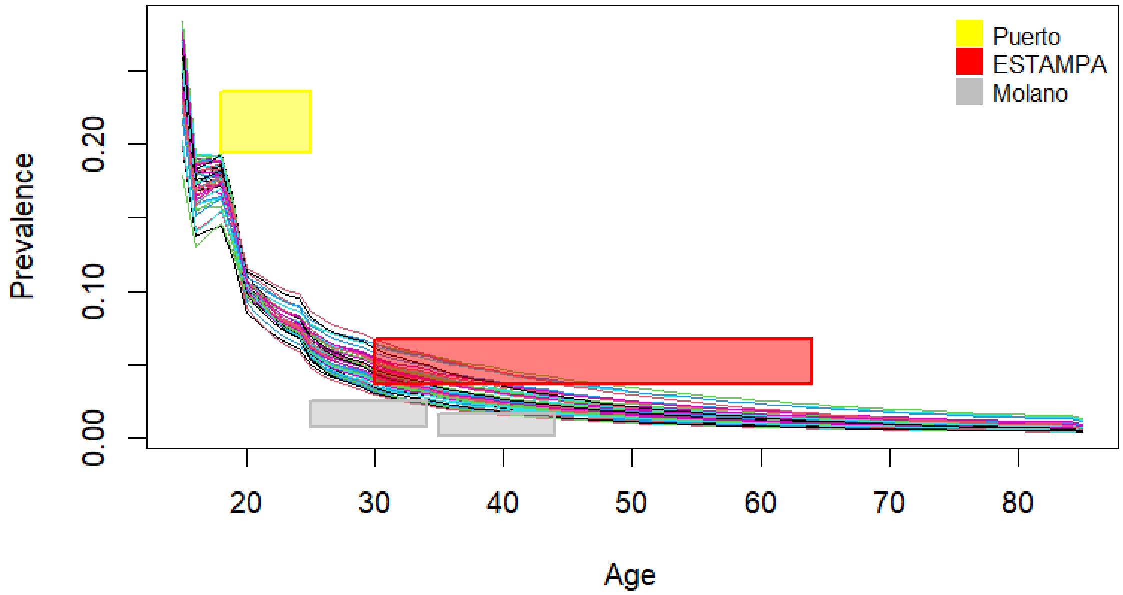

A

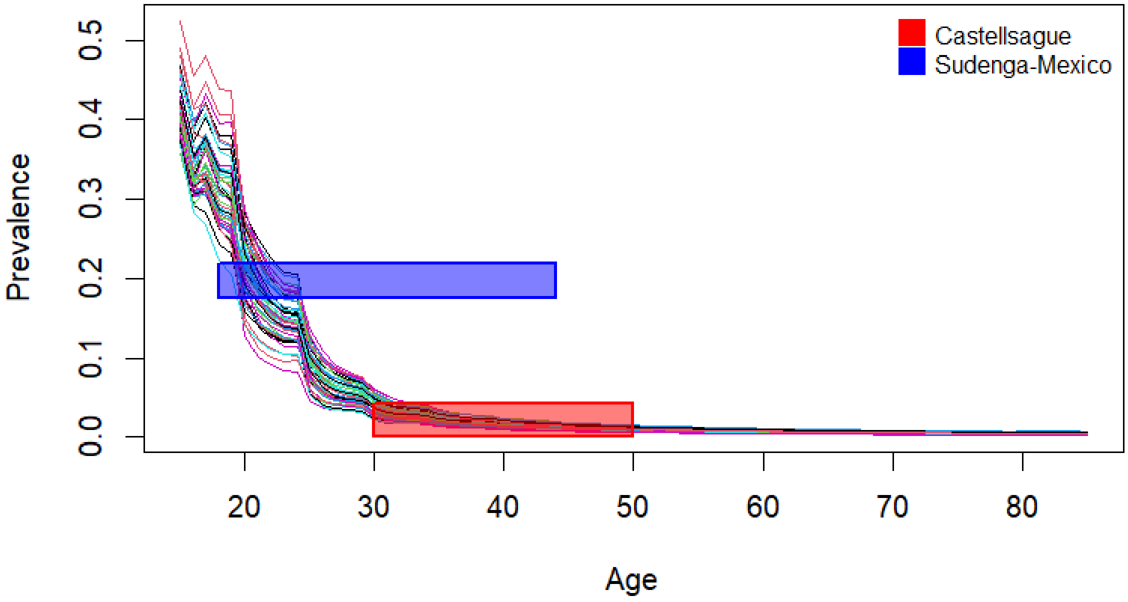

B

Figure S11. Age-specific prevalence of non-vaccine carcinogenic HPV in females (A) [30,32,37] and males (B). [38,39]
Note: Model-simulated results from selected sets (lines) compared with reported prevalence from the literature (boxes). The boxes represent the age range reported in studies (length) and the confidence interval (height) of the reported prevalence in the studies.

ANALYSIS

Combination of groups of HPV types

We later combined the results from the three groups taking into consideration co-infections distributions found in the ESTAMPA study. [37] Specifically, 7% of HPV 31, 33, 45, 52, or 58 infections were also positive for HPV 16 or 18; 16% of HPV 31, 33, 45, 52, or 58 infections were co-infected with other non-vaccine carcinogenic HPV types; 8% of non-vaccine carcinogenic HPV infections were also positive for HPV 16 or 18; and 1% of infections included at least one type from each of the three groups.

Prevalence and incidence calculations

To align model outputs with epidemiological data, prevalence and incidence were calculated among the sexually active population only. For prevalence, the numerator included individuals in detectable infected health states (including both new and persistent infections), while latent infections were excluded. For incidence, the numerator included both new infections and reactivations.

Cervical cancer projections

First, we calculated a weighted relative reduction in crude carcinogenic HPV prevalence in five-years age groups based on HPV type distributions observed in cervical cancer. We used reported HPV type distributions for Colombia, where 74% of cervical cancers are attributable to HPV 16 or HPV 18, and 24% to HPV 31, 33, 45, 52, or 58. Cervical cancers associated with other non-vaccine HPV types were assumed to remain unchanged (Baena A, personal communication). Then, we applied these relative reductions to the crude cervical cancer incidence within the following age groups: 15 to 29; 30 to 44, 45 to 59, 60 to 74 and 75, and older. [41] Because age-specific cervical cancer incidence data were available only for broader age categories, incidence rates were assumed constant within each category and applied to the corresponding narrower age groups used in the model. For example, we used crude cervical cancer incidence rates for 15 to 29 years olds to calculate expected cancer incidence in groups 15 to 20, 21 to 24 and 25 to 29 years old, and applied the prevalence relative reductions from these same age groups to obtain the corresponding cervical cancer incidence projections in the same age groups. Finally, we standardized the age groups crude incidence using 2015 World Female Population as previous studies, [3] and in a sensitivity analysis the Segi/Doll 1960 standard population, which is the standard used for international age-standardizations by the International Agency for Research on Cancer (IARC). [42]

Sensitivity analysis

Our model assumes lifelong vaccine-induced immunity based on current evidence. [26,43] We conducted a sensitivity analysis to explore the potential impact of waning protection following a single-dose schedule in gender-neutral nonavalent vaccination scenarios. In this analysis, we assumed that vaccine-induced immunity fully wanes after 20 years, such that vaccination no longer confers protection against HPV infection.

RESULTS

Age-standardized HPV prevalence

We observed a small decline in age-standardized carcinogenic HPV prevalence over time even in scenarios with no vaccination, due to changes in the age structure and its effect on HPV transmission dynamics (Table S9). A marked decline in age-standardized HPV prevalence for the types included in the nonavalent vaccine was observed in both females (Figure S12) and males (Figure S13), contributing to a reduction in carcinogenic age-standardized HPV prevalence (Figure S14 and S15). As expected, the magnitude of the decline was greater with higher vaccination coverage.

The nonavalent vaccine outperformed the quadrivalent vaccine in reducing age-standardized HPV prevalence in both females and males over time, particularly at a vaccination coverage level of 90%. Both vaccines achieved a rapid reduction in age-standardized HPV prevalence shortly after the implementation of higher vaccination coverage (2020–2040), with the nonavalent vaccine showing a steeper reduction. Over time (2040–2100), the reduction stabilized but remained consistently larger for the nonavalent vaccine in both females and males. Notably, in the case of the nonavalent vaccine, there was less uncertainty around the estimates. In contrast, for the quadrivalent vaccine, wider ranges were observed, reflecting the uncertainty around the estimates. Considering gender-neutral vaccination, the difference between coverage levels was less marked than with girls-only vaccination. The decline in carcinogenic HPV prevalence differed by age group, it occurred earlier in younger cohorts for both quadrivalent (Figure S16) and nonavalent vaccines (Figure S17).

Age-standardized HPV incidence

The model predicted a small decline in age-standardized HPV incidence over time in unvaccinated cohorts. In contrast, a marked decline was observed for the HPV types included in the nonavalent vaccine (Figures S18 and S19). As expected, the decline was more pronounced with higher vaccination coverage. The model predicted a decline in new HPV infections with both vaccines and vaccination coverage levels (Figure S20 and S21). Although the decline in age-standardized HPV incidence was greater with the nonavalent vaccine and a 90% coverage, there was significant overlap in the estimates, with almost overlapping values for both coverage levels compared in the gender-neutral scenario. By 2030, females vaccinated with the nonavalent vaccine had an age-standardized carcinogenic HPV incidence of 13·0 cases per 100 woman-years (range: 11·2–14·9) with a coverage of 51% for girls and 16% for boys, and 10·8 cases per 100 woman-years (range: 8·9–12·7) with a coverage of 90% in the gender-neutral vaccination scenario. For the quadrivalent vaccine, the corresponding values were 14·9 cases per 100 woman-years (range: 12·7–16·7) with a coverage of 51% for girls and 16% for boys, and 14·0 cases per 100 woman-years (range: 12·2–15·8) with a coverage of 90%. In the girls-only vaccination scenario, there were slightly higher incidences, specially with lower coverage levels, while in the case of 90% coverage, over time the difference between girls-only and gender-neutral estimates was almost zero (Table S10 and S11).

Breakthrough infections and herd immunity

Results on breakthrough infections and herd immunity are shown in Table S12.

Projections on cervical cancer incidence

The projected impact of HPV prevalence reduction in cervical cancer incidence is shown in Figure S22 as well as Tables S13 (age- standardized using 2015 World Female Population) and Table S14 (1996 Segi Segi/Doll standard population). The modest gap between the HPV type–attributable fractions and the projected reduction in cervical cancer reflects residual transmission and breakthrough infections allowed in the model, leading to a small number of vaccine-type cancers persisting even under near-elimination scenarios.

Sensitivity analysis for waning vaccine-induced immunity

Results (Table S15) are consistent with the main analysis, showing a modest delay in achieving cervical cancer elimination (approximately three years under 90% coverage and eight years under 51% coverage).

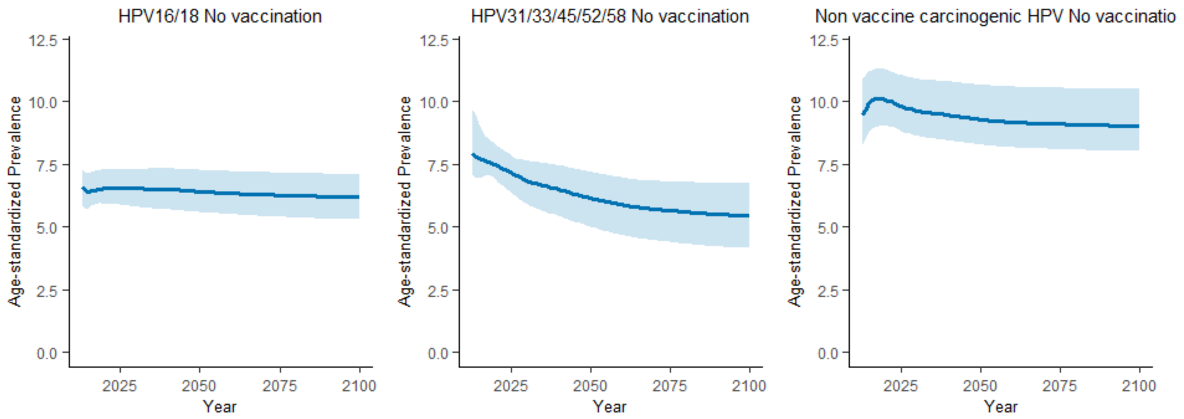
HPV16/18 No vaccination
HPV31/33/45/52/58 No vaccination
Non vaccine carcinogenic HPV No vaccinatio
Age-standardized Prevalence
Year
0.0
2.5
5.0
7.5
10.0
12.5
2025
2050
2075
2100

A

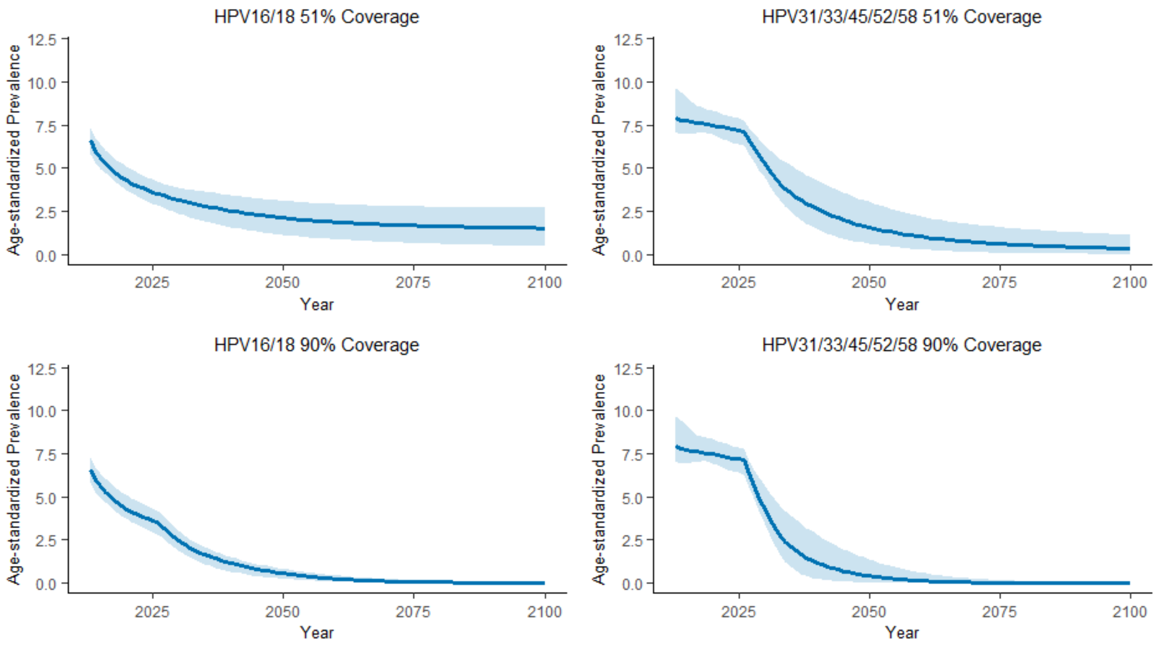
HPV16/18 51% Coverage
HPV31/33/45/52/58 51% Coverage
HPV16/18 90% Coverage
HPV31/33/45/52/58 90% Coverage
Age-standardized Prevalence
Year
0.0
2.5
5.0
7.5
10.0
12.5
2025
2050
2075
2100

B

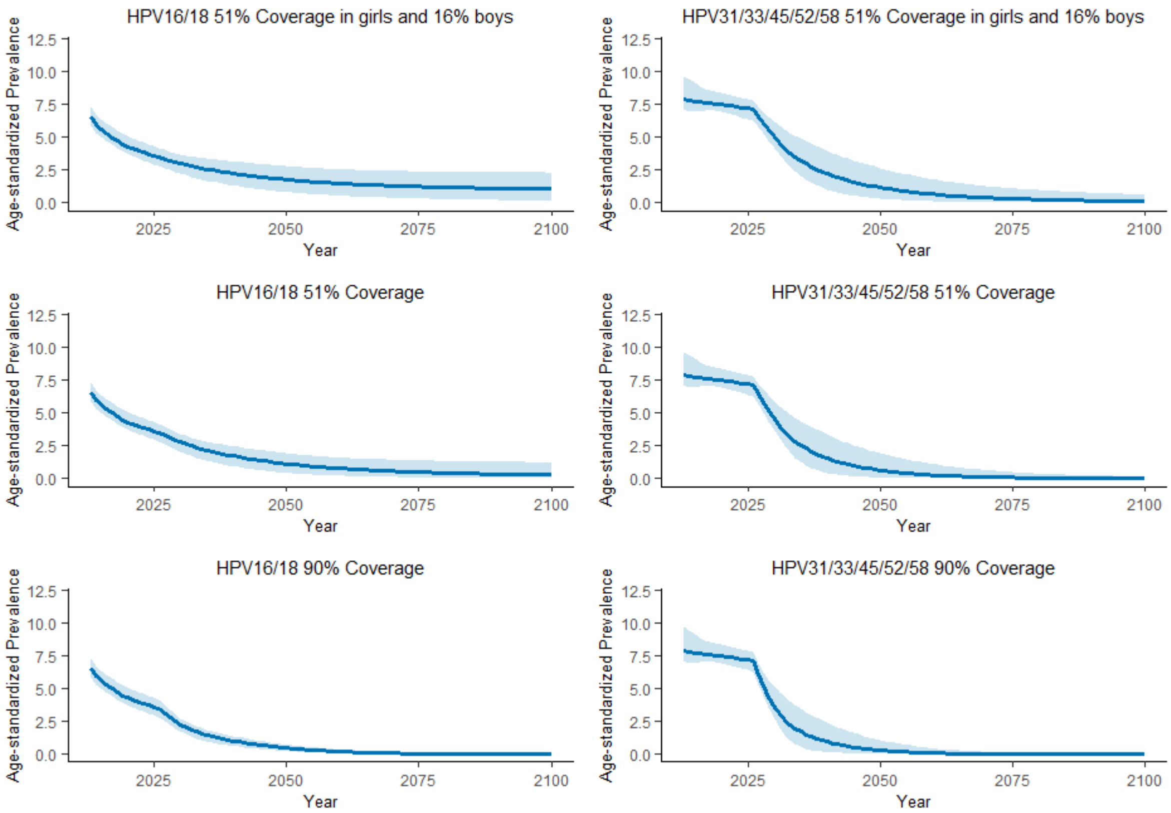

C

Figure S12. Age-standardized HPV prevalence by HPV types in females (A) in the no vaccination scenario, (B) in girls-only scenario, and (C) in gender-neutral scenario.

Note: The reduction in HPV16/18 is with either the quadrivalent or the nonavalent HPV vaccines as both vaccines have proven to be equally effective, while the reduction in HPV 31/33/45/52/58 prevalence is observed only in the case of the nonavalent HPV vaccine. Vaccination with the quadrivalent vaccine started in 2013 and with the nonavalent vaccine in 2026.

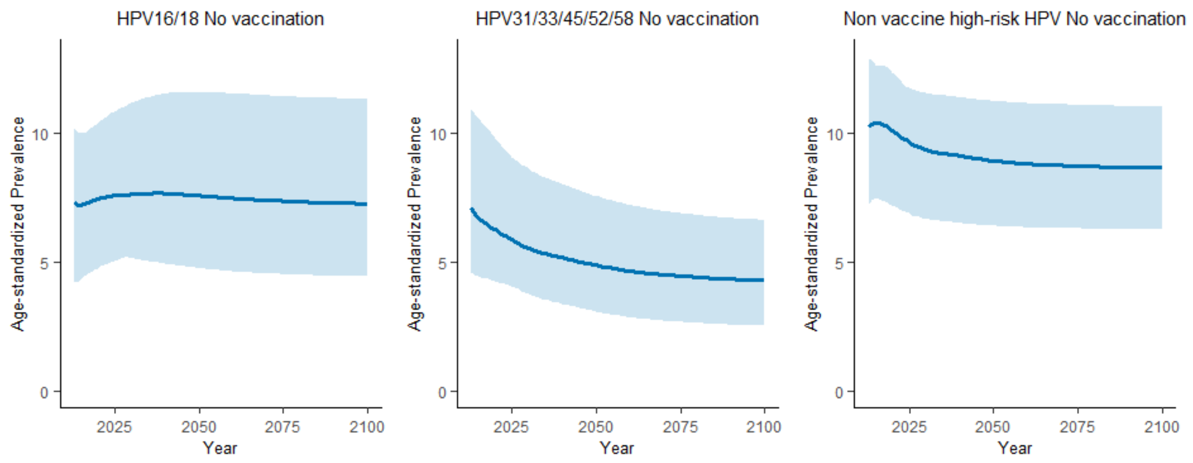
HPV16/18 No vaccination
HPV31/33/45/52/58 No vaccination
Non vaccine high-risk HPV No vaccination
Age-standardized Prevalence
0
5
10
2025
2050
2075
2100
Year

A

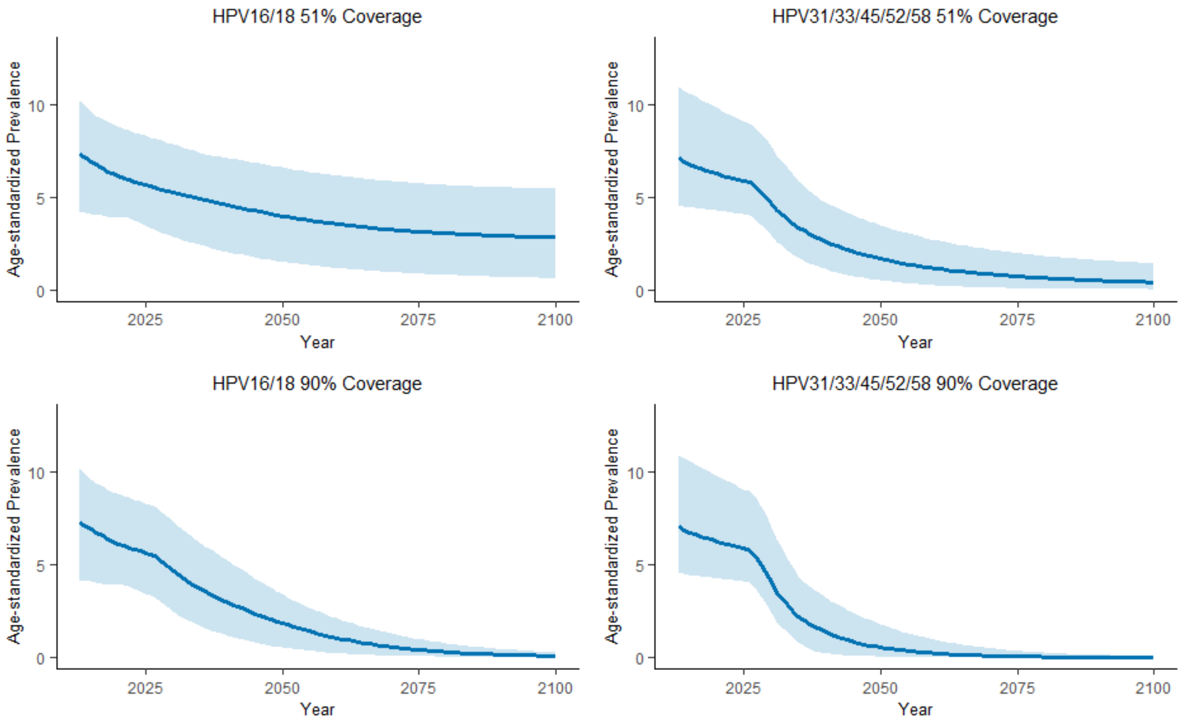
HPV16/18 51% Coverage
HPV31/33/45/52/58 51% Coverage
HPV16/18 90% Coverage
HPV31/33/45/52/58 90% Coverage
Age-standardized Prevalence
0
5
10
2025
2050
2075
2100
Year

B

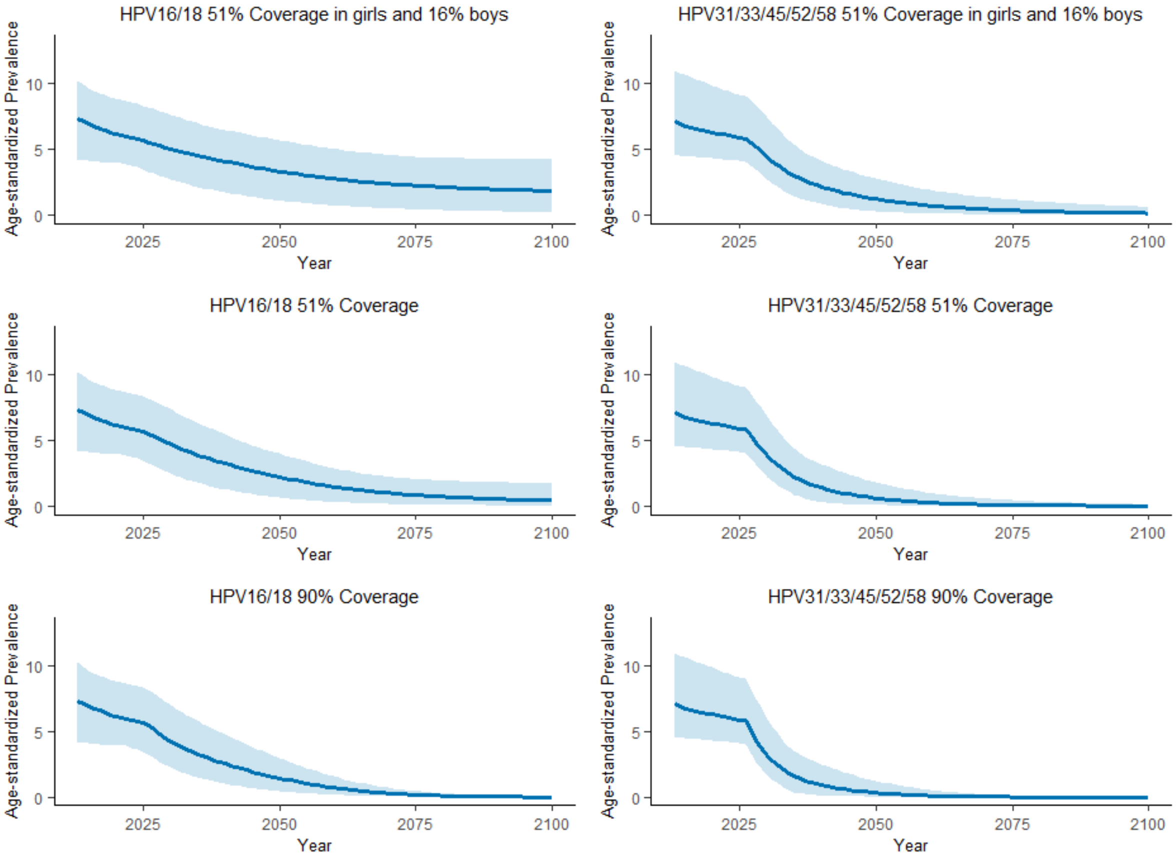

C

Figure S13. Age-standardized HPV prevalence by HPV types in males (A) in the no vaccination scenario, (B) in girls-only scenario, and (C) in gender-neutral scenario.

Note: Vaccination coverage corresponds to girls-only vaccination, thus, the reduction in HPV prevalence is due to herd-effect immunity. The reduction in HPV16/18 is with either the quadrivalent or the nonavalent HPV vaccines as both vaccines have proven to be equally effective, while the reduction in HPV 31/33/45/52/58 prevalence is observed only in the case of the nonavalent HPV vaccine. Vaccination with the quadrivalent vaccine started in 2031 and with the nonavalent vaccine in 2026.

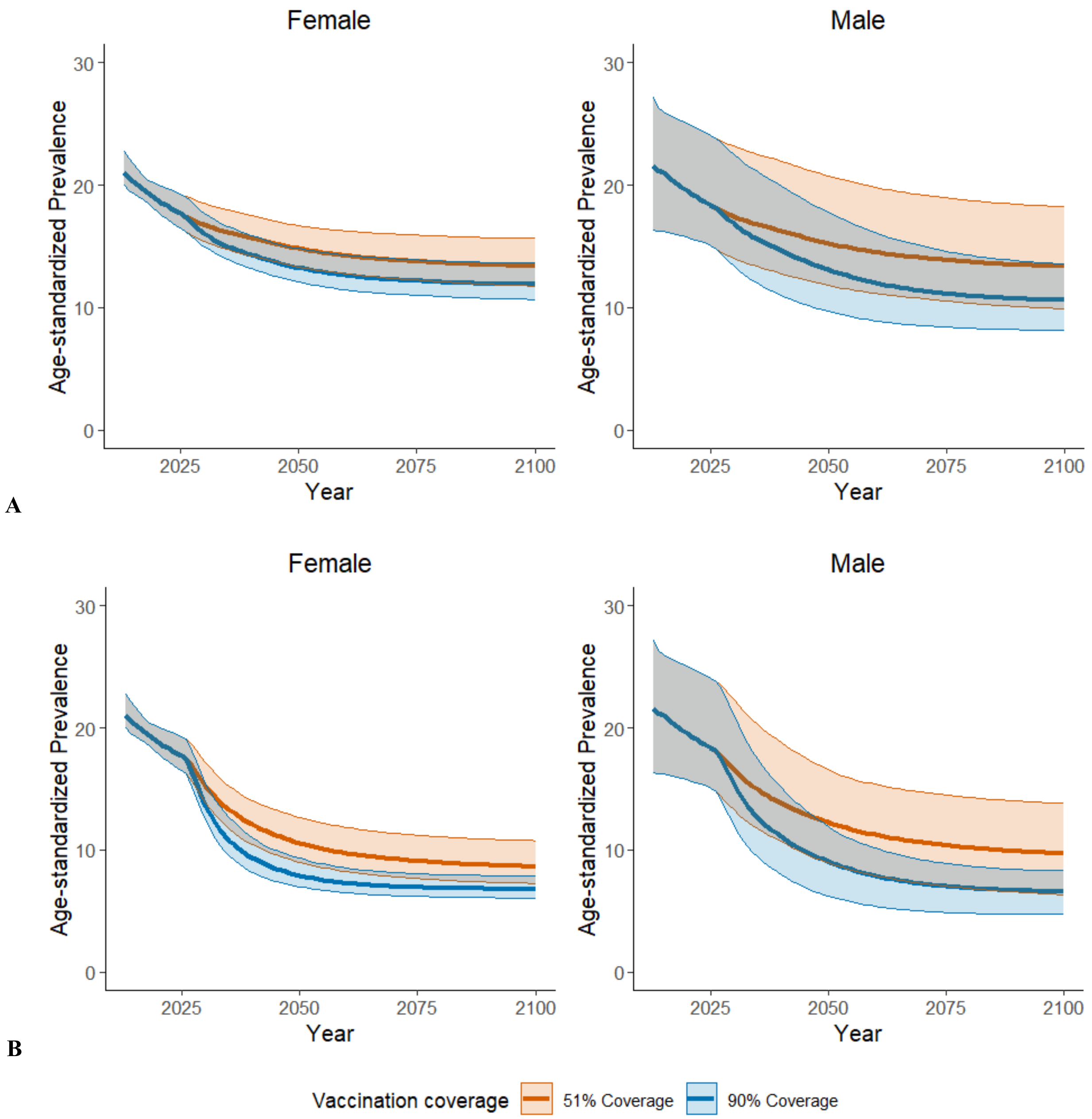

Figure S14. Age-standardized HPV prevalence for carcinogenic HPV types after girls-only vaccination with the (A) quadrivalent and (B) nonavalent HPV vaccines with different vaccination coverages.

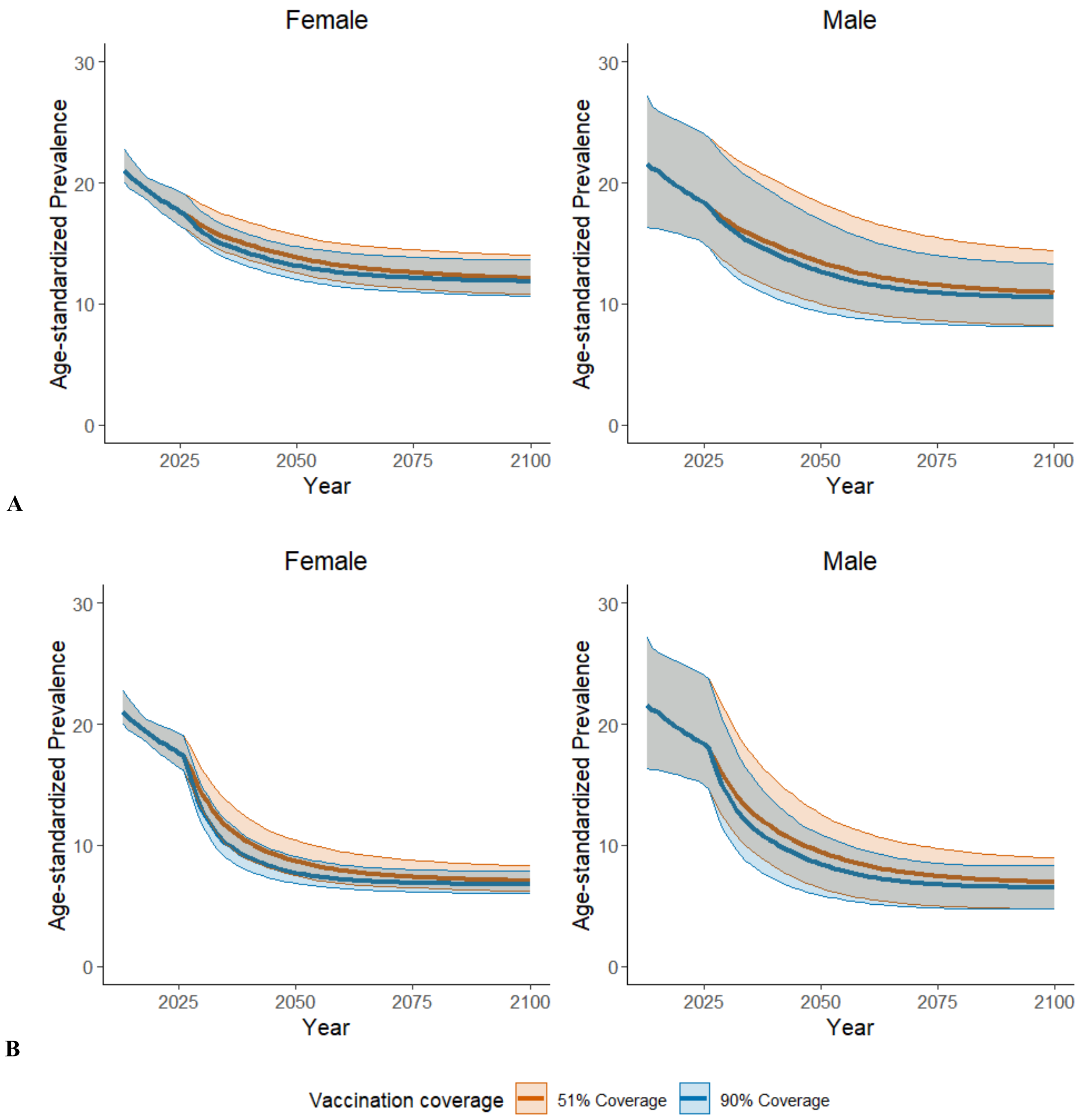

Figure S15. Age-standardized HPV prevalence for carcinogenic HPV types after gender-neutral vaccination with the (A) quadrivalent and (B) nonavalent HPV vaccines with different vaccination coverages.

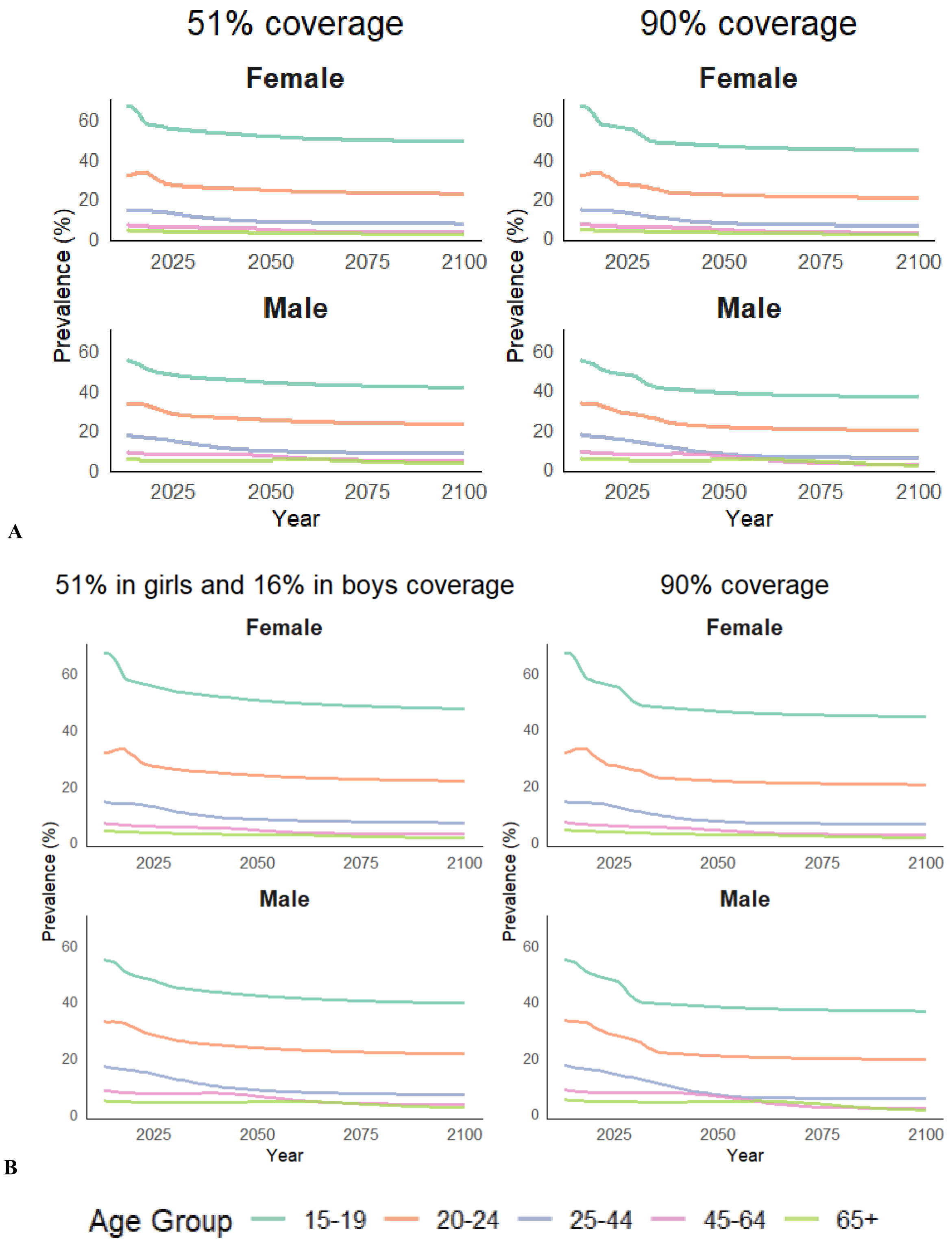

Figure S16. Carcinogenic HPV prevalence by age group and sex after (A) girls-only (B) and gender-neutral vaccination with the quadrivalent vaccine.

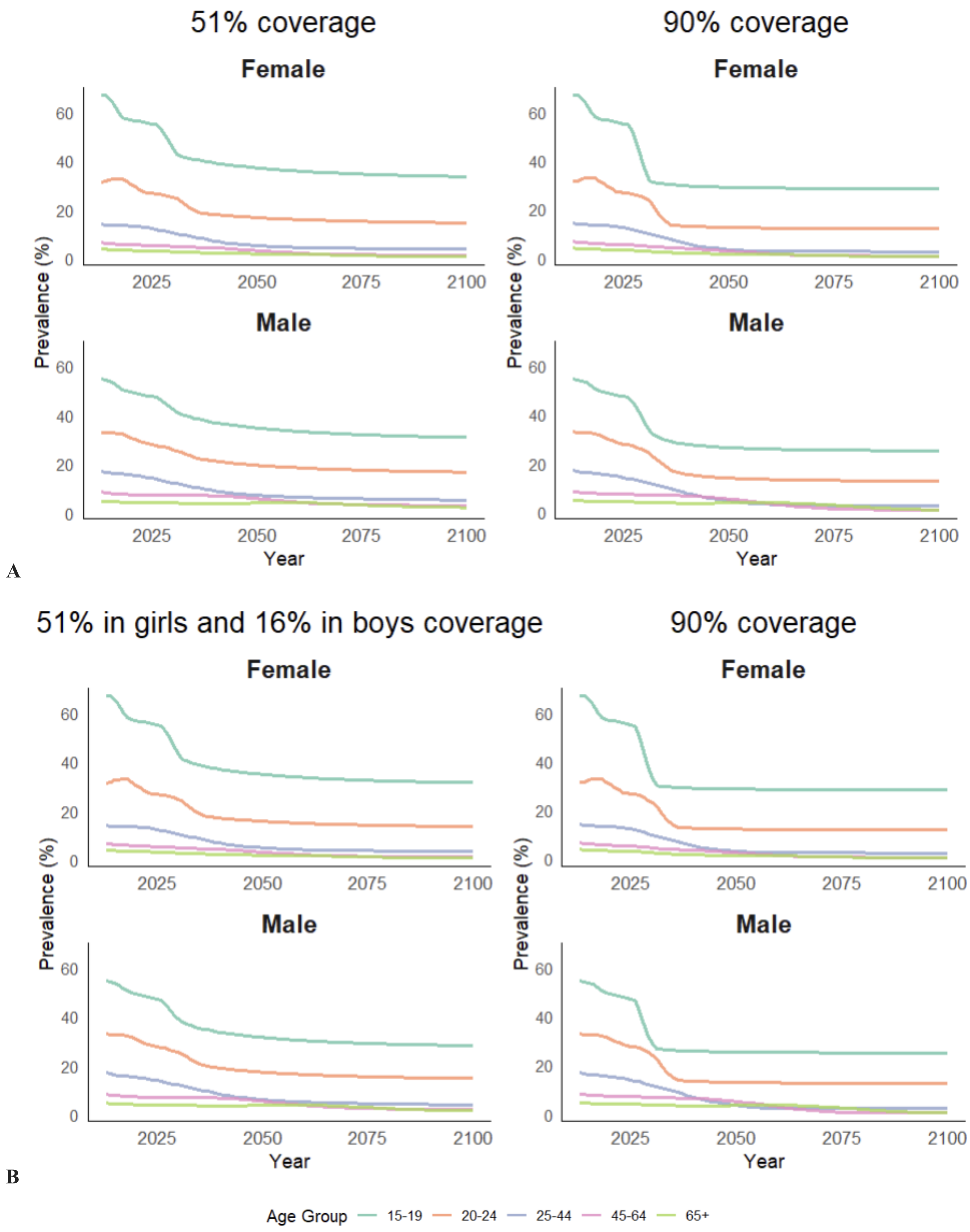

Figure S17. Carcinogenic HPV prevalence by age group and sex after (A) girls-only (B) and gender-neutral vaccination with the nonavalent vaccine.

Table S9. Age-standardized carcinogenic HPV prevalence over time among individuals aged 15 years and older, for groups of HPV types according to HPV vaccination scenario and coverage. The values correspond to the mean (range) of the 50 estimates from the parameter sets.

	HPV vaccination scenario	HPV vaccination coverage	Female			Male		
			Year 2030	Year 2040	Year 2100	Year 2030	Year 2040	Year 2100
HPVs 16/18[b]	No vaccine[e]	0%	6·5% (5·8-7·3)	6·5% (5·7-7·3)	6·2% (5·3-7·1)	7·6% (5·2-11·2)	7·7% (5·0-11·6)	7·3% (4·5-11·3)
	Girls-only	51%	3·2% (2·4-3·9)	2·5% (1·6-3·4)	1·5 (0·5-2·7)	5·3% (2·9-7·9)	4·6% (2·1-7·1)	2·8% (0·6-5·5)
		90%	2·4% (1·9-3·0)	1·2% (0·7-1·5)	0·01% (<0·01-0·04)	4·7% (2·5-7·4)	3·0% (1·2-5·2)	0·1% (<0·01--0·3)
	Gender-neutral	G51% & B16%	3·0% (2·2-3·7)	2·3% (1·3-3·2)	1·0% (0·1-2·3)	5·0% (2·7-7·6)	4·1% (1·7-6·4)	1·8% (0·2-4·2)
		51%	2·8% (2·1-3·4)	1·7% (0·9-2·4)	0·3% (0·01-1·2)	4·7% (2·5-7·3)	3·2% (1·3-5·5)	0·4% (0·01-1·7)
		90%	2·2% (1·7-2·8)	1·0% (0·6-1·4)	<0·01% (<0·01-0·01)	4·2% (2·2-7·0)	2·5% (1·0-4·8)	0·01% (<0·01-0·03)
HPVs 31/33/45/52/58	No vaccine[e]	0%	6·9% (5·9-7·7)	6·5% (5·5-7·5)	5·5% (4·2-6·7)	5·5% (3·8-8·6)	5·2% (3·4-8·1)	4·3% (2·5-6·7)
	Girls-only	51%	5·2% (4·4-6·3)	2·6% (1·5-4·3)	0·4% (0·01-1·1)	4·7% (2·9-7·7)	2·6% (1·1-4·7)	0·4% (0·01-1·4)
		90%	4·2% (3·4-5·6)	1·2% (0·2-2·8)	<0·01% (<0·01-0·02)	4·0% (2·4-7·0)	1·3% (0·2-3·2)	0·01% (<0·01-0·06)
	Gender-neutral	G51% & B16%	5·0% (4·2-6·2)	2·2% (1·0-4·0)	0·1% (<0·01-0·6)	4·4% (2·7-7·4)	2·1% (0·8-4·1)	0·1% (<0·01-0·6)
		51%	4·5% (3·7-5·9)	1·5% (0·4-3·4)	0·01% (<0·01-0·1)	3·8% (2·2-6·8)	1·4% (0·3-3·2)	0·01% (<0·01-0·1)
		90%	3·6% (2·7-5·2)	0·9% (0·1-2·4)	<0·01% (<0·01-<0·01)	3·1% (1·7-6·0)	0·9% (0·1-2·5)	<0·01% (<0·01-<0·01)
HPVs 16/18/31/33/45/52/58	No vaccine[e]	0%	12·9% (11·9-14·1)	12·5% (11·3-13·9)	11·2% (9·9-12·4)	12·7% (9·5-17·9)	12·5% (9·3-17·6)	11·3% (8·2-16·1)
	Girls-only	51%	8·0% (6·8-9·6)	4·9% (3·3-7·0)	1·9% (0·6-2·9)	9·6% (6·9-14·5)	7·0% (4·5-11·2)	3·2% (0·9-6·0)
		90%	6·3% (5·2-8·2)	2·2% (1·2-4·0)	0·01% (<0·01-0·04)	8·4% (6·0-13·2)	4·2% (2·4-7·5)	0·09% (0·01-0·3)
	Gender neutral	G51% & B16%	7·6% (6·4-9·3)	4·3% (2·6-6·4)	1·2% (0·2-2·3)	9·0% (6·5-13·9)	6·0% (3·7-10·0)	1·9% (0·3-4·2)
		51%	7·0% (5·7-8·8)	3·1% (1·7-5·3)	0·3% (0·01-1·2)	8·2% (5·8-13·0)	4·5% (2·6-7·9)	0·4% (0·02-1·7)
		90%	5·6% (4·4-7·5)	1·9% (1·0-3·4)	<0·01% (<0·01-0·01)	7·1% (4·9-11·7)	3·4% (2·0-6·0)	0·01% (<0·01-0·03)
Other carcinogenic HPVs	No vaccine	0%	9·6% (8·6-10·9)	9·5% (8·5-10·8)	9·0% (8·0-10·5)	9·4% (6·7-11·6)	9·1% (6·5-11·4)	8·7% (6·3-11·1)
All carcinogenic HPVs	No vaccine[a]	0%	20·1% (19·0-21·8)	19·7% (18·4-21·4)	18·1% (16·5-19·9)	19·8% (16·2-25·7)	19·4% (16·0-25·3)	17·8% (14·7-23·3)
	Girls-only 9v vaccine	51%	15·2% (13·8-17·1)	12·1% (10·4-14·0)	8·6% (7·3-10·8)	16·6% (13·3-22·3)	13·9% (10·7-18·8)	9·7% (6·3-13·8)
		90%	13·6% (12·5-15·5)	9·4% (8·2-11·0)	6·8% (6·1-7·9)	15·5% (12·0-21·0)	11·1% (8·0-15·1)	6·6% (4·7-8·3)
	Gender neutral	G51% & B16%	14·9% (13·5-16·8)	11·4% (9·8-13·4)	7·9% (6·6-9·8)	16·1% (12·7-21·8)	12·9% (9·7-17·6)	8·5% (5·3-11·9)
		51%	14·2% (12·9-16·3)	10·3% (8·8-12·2)	7·1% (6·2-8·3)	15·3% (11·9-20·8)	11·4% (8·3-15·5)	6·9% (4·8-9·0)

	9v vaccine	90%	12·8% (11·6-14·8)	9·0% (7·9-10·6)	6·8% (6·0-7·9)	14·2% (10·7-19·5)	10·2% (7·2-13·6)	6·5% (4·7-8·3)
	Girls-only 4v vaccine	51%	16·8% (15·4-18·5)	15·7% (14·2-17·6)	13·4% (11·8-15·7)	17·4% (14·0-23·2)	16·3% (12·8-22·0)	13·4% (9·9-18·3)
		90%	16·1% (15·0-17·7)	14·3% (13·2-15·9)	11·9% (10·7-13·6)	16·9% (13·4-22·5)	14·7% (11·0-19·9)	10·6% (8·2-13·6)
	Gender neutral 4v vaccine	G51% & B16%	16·6% (15·3-18·4)	15·4% (14·0-17·3)	12·9% (11·3-15·2)	17·2% (13·8-22·9)	15·8% (12·2-21·3)	12·4% (9·1-16·9)
		51%	16·4% (15·2-18·1)	14·9% (13·6-16·7)	12·1% (10·8-14·0)	16·9% (13·4-22·5)	14·9% (11·3-20·3)	11·0% (8·3-14·4)
		90%	15·9% (14·9-17·5)	14·2% (13·1-15·7)	11·9% (10·7-13·6)	16·4% (12·8-22·0)	14·2% (10·6-19·1)	10·5% (8·1-13·3)

HPV: Human papillomavirus, G: Girls, B: Boys, 9v: Nonavalent, 4v: Quadrivalent. Note: HPVs 16/18 refers to infection with HPV16 and/or 18. HPVs 31, 33, 45, 52, and 58 refers to infection with any of HPV types 31, 33, 45, 52, and 58 and all carcinogenic refers to infection with any of HPV types 16, 18, 31, 33, 35, 39, 45, 51, 52, 56, 58, 59, and 68.

[a] Changes in prevalence in the no vaccine scenario are due solely to demographic changes expected over the 21st century.

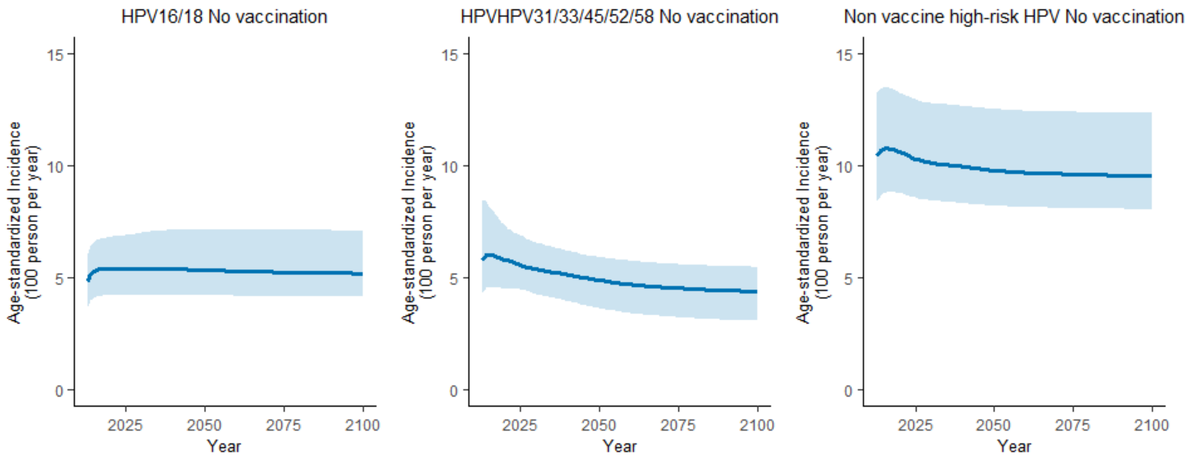
HPV16/18 No vaccination
HPVHPV31/33/45/52/58 No vaccination
Non vaccine high-risk HPV No vaccination
Age-standardized Incidence
(100 person per year)
0
5
10
15
2025
2050
2075
2100
Year

A

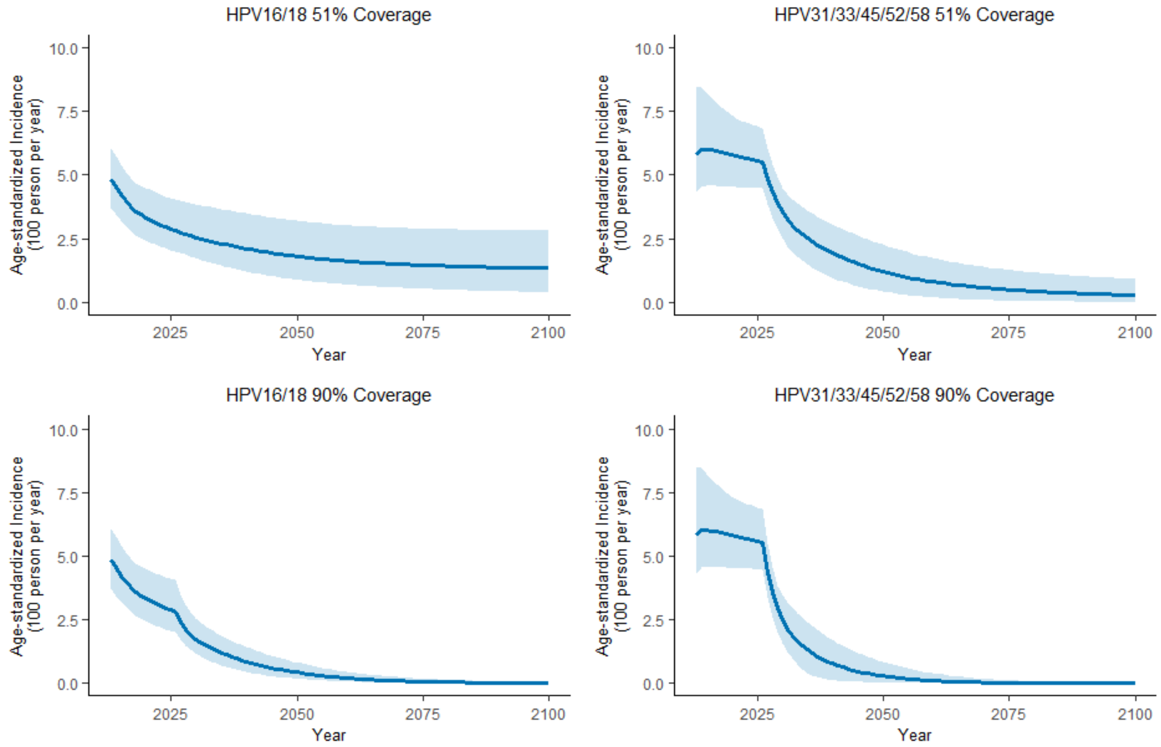
HPV16/18 51% Coverage
HPV31/33/45/52/58 51% Coverage
HPV16/18 90% Coverage
HPV31/33/45/52/58 90% Coverage
Age-standardized Incidence
(100 person per year)
0.0
2.5
5.0
7.5
10.0
2025
2050
2075
2100
Year

B

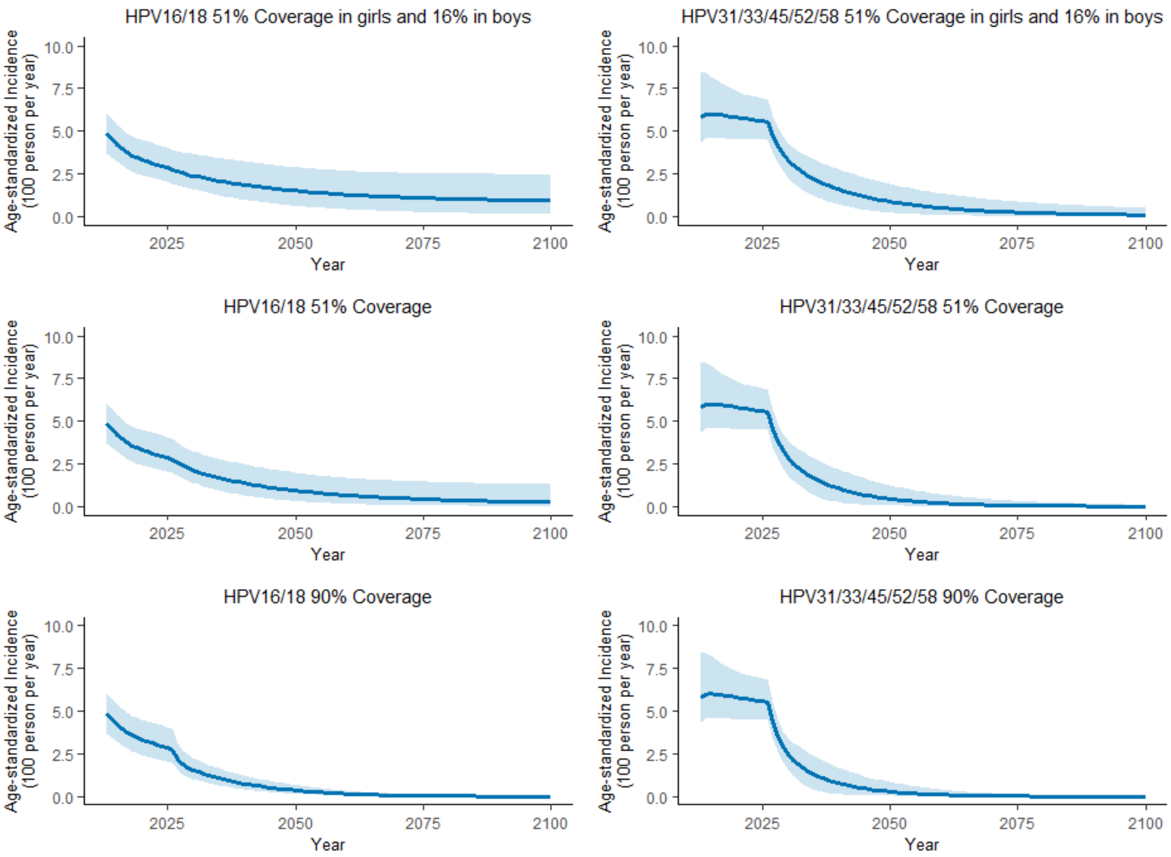

C

Figure S18. Age-standardized HPV incidence rates (per 100 person-years) by HPV types in females (A) in the no vaccination scenario, (B) in girls-only scenario, and (C) in gender-neutral scenario.

Note: The reduction in HPV16/18 is with either the quadrivalent or the nonavalent HPV vaccines as both vaccines have proven to be equally effective, while the reduction in HPV 31/33/45/52/58 prevalence is observed only in the case of the nonavalent HPV vaccine.

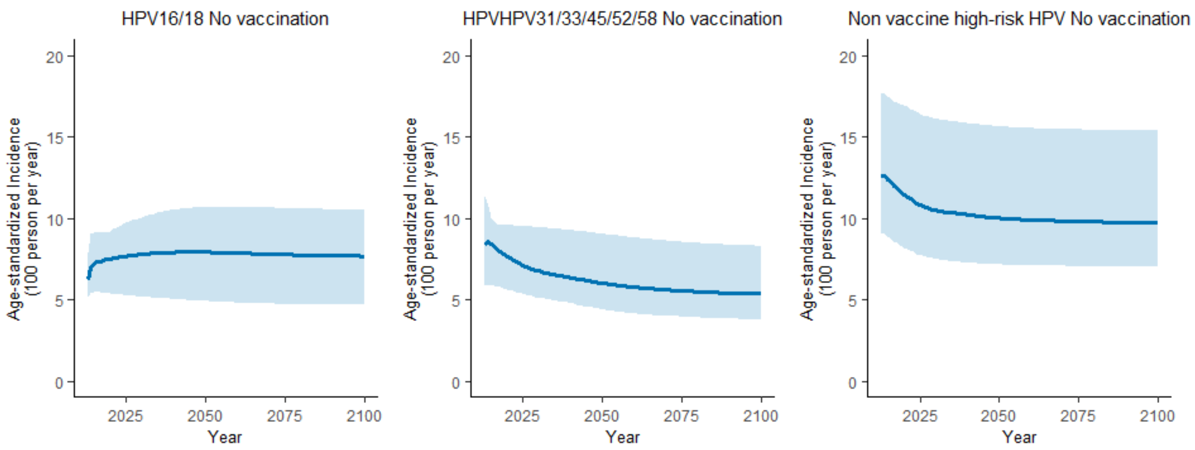
HPV16/18 No vaccination
HPVHPV31/33/45/52/58 No vaccination
Non vaccine high-risk HPV No vaccination
Age-standardized Incidence
(100 person per year)
Year
0
5
10
15
20
2025
2050
2075
2100

A

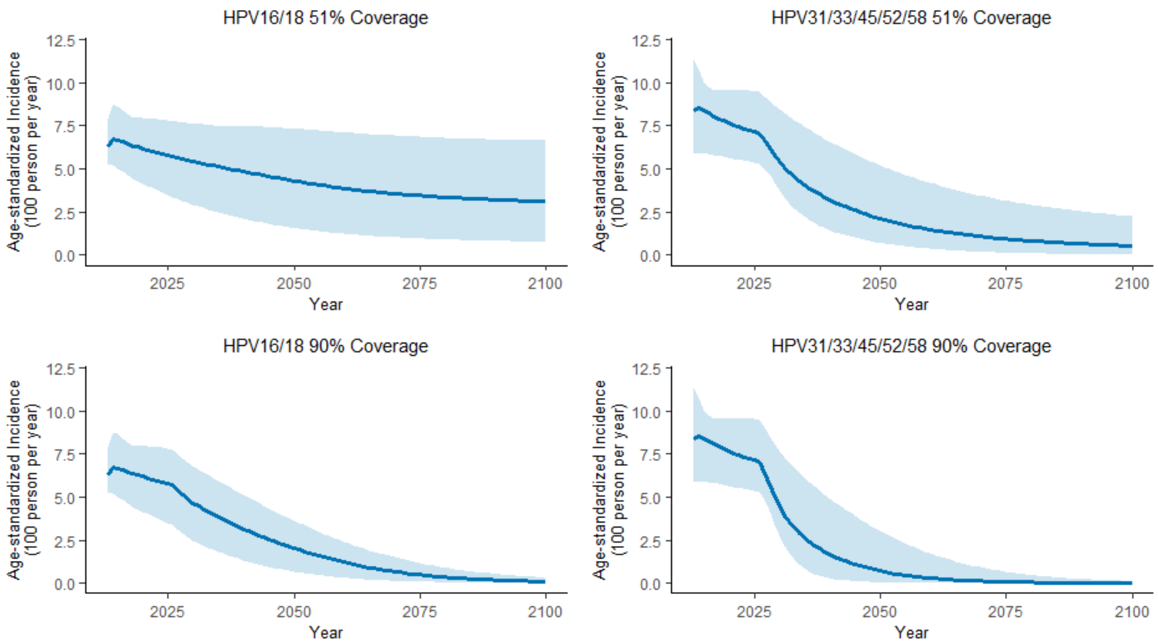
HPV16/18 51% Coverage
HPV31/33/45/52/58 51% Coverage
HPV16/18 90% Coverage
HPV31/33/45/52/58 90% Coverage
Age-standardized Incidence
(100 person per year)
Year
0.0
2.5
5.0
7.5
10.0
12.5
2025
2050
2075
2100

B

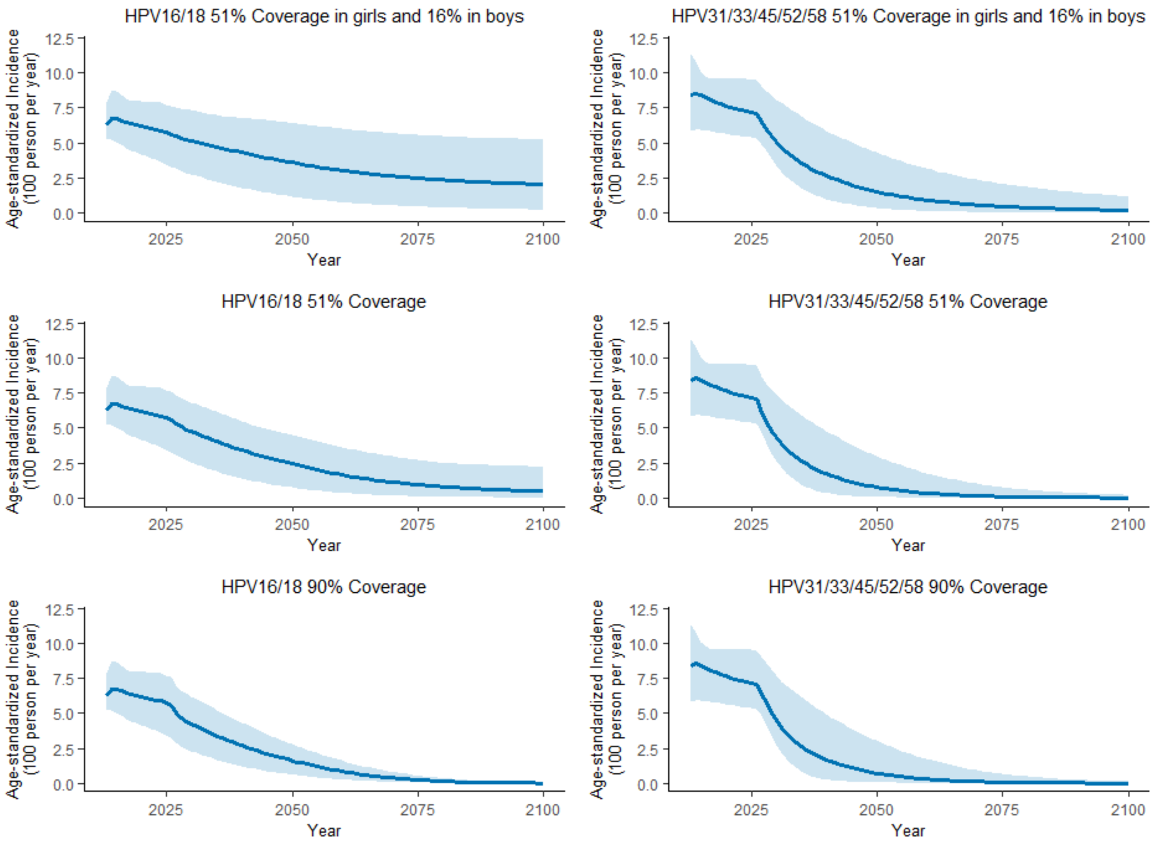

C

Figure S19. Age-standardized HPV incidence rates (per 100 person-years) by HPV types in males (A) in the no vaccination scenario, (B) in girls-only scenario, and (C) in gender-neutral scenario.

Note: The reduction in HPV16/18 is with either the quadrivalent or the nonavalent HPV vaccines as both vaccines have proven to be equally effective, while the reduction in HPV 31/33/45/52/58 prevalence is observed only in the case of the nonavalent HPV vaccine.

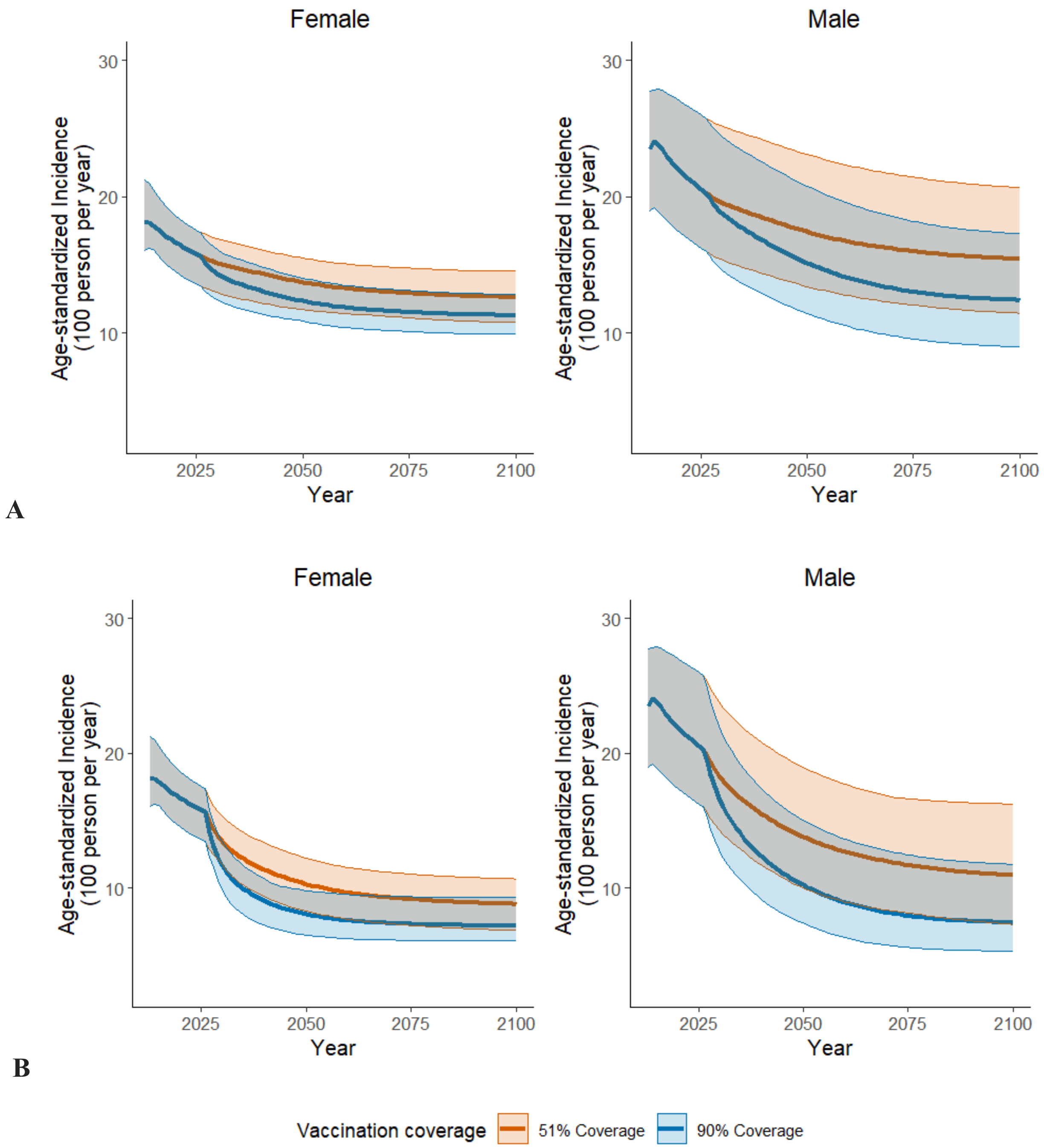

Figure S20. Age-standardized HPV incidence rates (per 100 person-years) for carcinogenic HPV types after girls-only vaccination with the (A) quadrivalent and (B) the nonavalent HPV vaccines with different vaccination coverages.

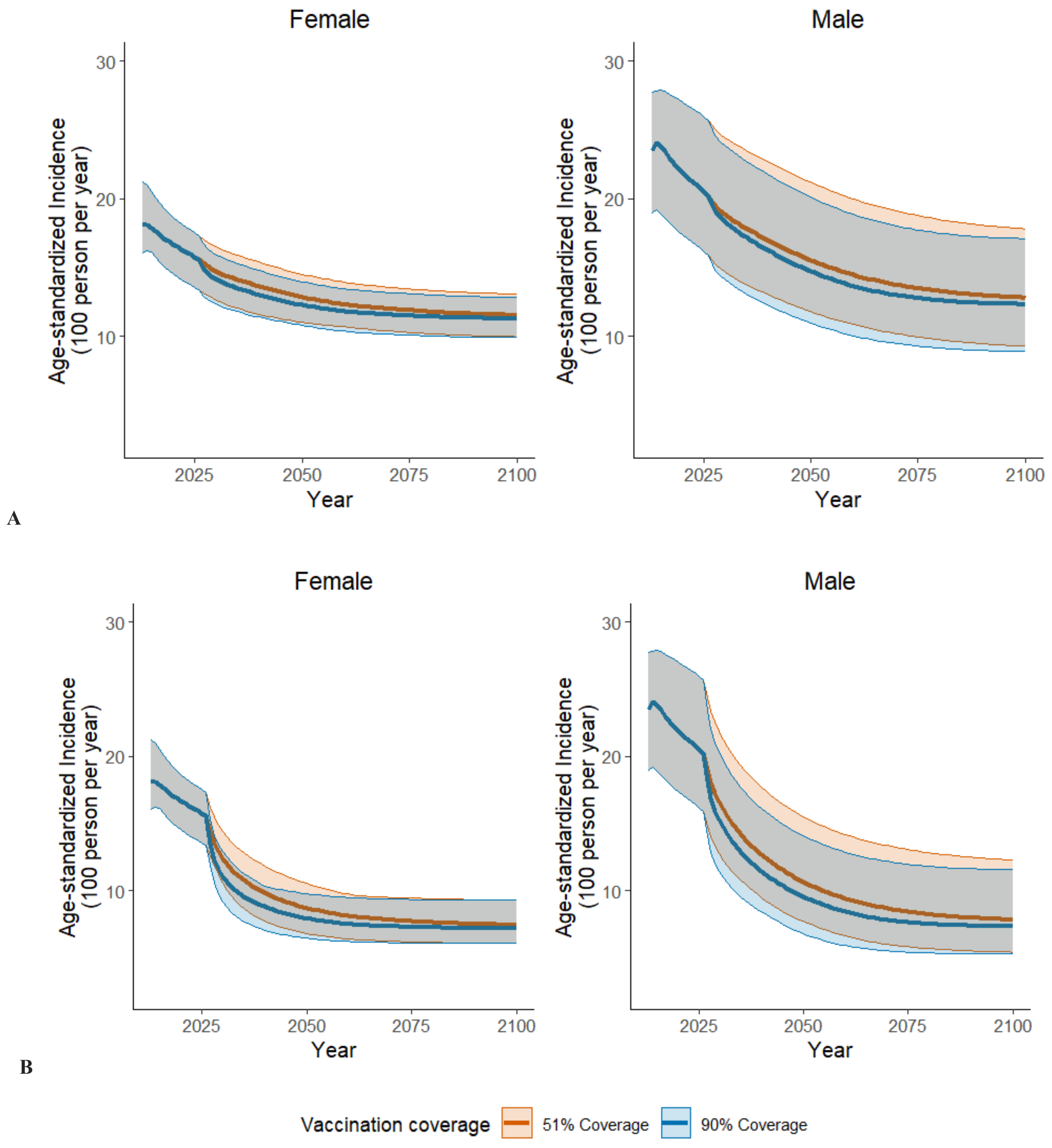

Figure S21. Age-standardized HPV incidence rates (per 100 person-years) for carcinogenic HPV types after gender-neutral vaccination with the (A) quadrivalent and (B) the nonavalent HPV vaccines with different vaccination coverages.

Table S10. Age-standardized carcinogenic HPV incidence rates (per 100 person-years) by HPV type group, HPV vaccine, vaccination strategy, and vaccination coverage. The values correspond to the mean (range) of the 50 estimates from the parameter sets.

	Vaccination strategy	Vaccination coverage	Female Year 2030	Female Year 2040	Female Year 2100	Male Year 2030	Male Year 2040	Male Year 2100
HPV type groups								
HPV 16/18	No vaccine	0%	5·4 (4·2-7·1)	5·4 (4·2-7·1)	5·2 (4·1-7·1)	7·9 (5·2-10·3)	7·9 (5·0-10·6)	7·6 (4·6-10·3)
	Girls-only	51%	2·5 (1·7-3·8)	2·1 (1·2-3·5)	1·3 (0·4-2·8)	5·4 (2·8-7·7)	4·7 (2·0-7·5)	3·1 (0·7-6·5)
		90%	1·6 (1·1-2·5)	0·8 (0·4-1·3)	0·01 (<0·01-0·04)	4·5 (2·3-6·7)	2·9 (1·2-4·8)	0·08 (<0·01-0·3)
	Gender-neutral	G51% & B16%	2·3 (1·5-3·6)	1·8 (0·9-3·2)	0·9 (0·1-2·4)	5·0 (2·6-7·3)	4·2 (1·6-6·7)	2·0 (0·2-5·2)
		51%	2·1 (1·3-3·2)	1·3 (0·6-2·4)	0·2 (0·01-1·3)	4·6 (2·3-6·7)	3·2 (1·2-5·2)	0·5 (0·01-2·2)
		90%	1·5 (0·9-2·2)	0·7 (0·3-1·1)	<0·01 (<0·01-0·01)	4·0 (2·1-6·0)	2·4 (1·1-3·9)	0·01 (<0·01-0·03)
HPV 31/33/45/52/58	No vaccine	0%	5·4 (4·3-6·6)	5·1 (4·0-6·2)	4·4 (3·1-5·5)	6·7 (5·1-9·4)	6·3 (4·8-9·2)	5·3 (3·8-8·2)
	Girls-only	51%	3·5 (2·5-4·4)	1·8 (0·9-2·9)	0·3 (0·01-0·9)	5·3 (3·7-8·3)	3·0 (1·4-6·3)	0·5 (0·01-2·2)
		90%	2·4 (1·5-3·3)	0·7 (0·1-1·5)	<0·01 (<0·01-0·01)	4·3 (2·7-7·4)	1·4 (0·2-4·5)	0·01 (<0·01-0·1)
	Gender-neutral	G51% & B16%	3·3 (2·3-4·2)	1·5 (0·7-2·6)	0·1 (<0·01-0·5)	4·9 (3·3-7·9)	2·4 (0·9-5·5)	0·2 (<0·01-1·1)
		51%	2·8 (1·8-3·8)	0·9 (0·2-2·0)	0·1 (<0·01-0·1)	4·1 (2·5-7·1)	1·5 (0·3-4·3)	0·1 (<0·01-0·2)
		90%	1·9 (1·0-2·9)	0·5 (0·1-1·2)	<0·01 (<0·01-<0·01)	3·1 (1·6-6·2)	0·9 (0·1-3·2)	<0·01 (<0·01-0·01)
Others high risk HPV	NI	0%	10·1 (8·4-12·8)	10·0 (8·3-12·7)	9·6 (8·1-12·4)	10·4 (7·4-16·1)	10·2 (7·3-15·8)	9·8 (7·0-15·4)
HPV 16/18/31/33/45/52/58	No vaccine	0%	10·3 (8·2-12·0)	10·1 (7·8-11·8)	9·1 (6·9-11·0)	13·9 (10·5-17·8)	13·6 (10·0-17·7)	12·3 (8·6-16·2)
	Girls-only	51%	5·8 (4·1-6·8)	3·7 (2·1-5·1)	1·5 (0·5-2·8)	10·2 (6·8-14·1)	7·4 (3·7-11·4)	3·3 (0·7-6·2)
		90%	3·9 (2·6-5·1)	1·4 (0·7-2·5)	0·01 (<0·01-0·03)	8·4 (5·3-12·2)	4·1 (1·6-7·3)	0·08 (<0·01-0·25)
	Gender-neutral	G51% & B16%	5·4 (3·7-6·5)	3·2 (1·7-4·6)	0·9 (0·1-2·3)	9·5 (6·2-13·3)	6·2 (2·9-10·0)	2·0 (0·2-4·8)
		51%	4·7 (3·1-5·9)	2·2 (1·1-3·6)	0·2 (0·01-1·2)	8·3 (5·1-11·8)	4·4 (1·8-7·4)	0·4 (0·01-2·0)
		90%	3·2 (2·1-4·5)	1·1 (0·5-2·0)	<0·01 (<0·01-0·01)	6·8 (3·8-10·0)	3·1 (1·3-5·5)	0·01 (<0·01-0·03)
All carcinogenic HPV	No vaccine	0%	18·0 (16·0-20·0)	17·6 (15·7-19·7)	16·4 (14·7-18·5)	21·9 (17·4-27·4)	21·5 (16·9-26·9)	19·8 (15·2-24·9)
Vaccination strategy for all carcinogenic HPV								
Nonavalent vaccine	Girls-only	51%	13·3 (11·6-15·2)	11·2 (9·2-13·2)	8·8 (6·8-10·7)	18·1 (14·1-23·6)	15·2 (11·5-20·6)	10·8 (7·2-16·1)
		90%	11·5 (9·7-13·3)	8·9 (7·1-10·5)	7·2 (3·1-9·3)	16·3 (12·6-21·7)	11·9 (8·7-16·9)	7·4 (5·3-11·7)
	Gender-neutral	G51% & B16%	13·0 (11·2-14·9)	10·7 (8·6-12·7)	8·2 (6·3-10·1)	17·4 (13·5-22·8)	14·1 (10·5-19·3)	9·4 (6·2-14·8)
		51%	12·2 (10·4-14·3)	9·7 (7·6-11·7)	7·4 (6·1-9·3)	16·2 (12·5-21·5)	12·2 (9·1-17·2)	7·8 (5·4-12·2)
		90%	10·8 (8·9-12·7)	8·6 (6·9-10·2)	7·2 (6·1-9·3)	14·7 (11·1-19·7)	10·9 (8·0-15·6)	7·3 (5·3-11·6)
Quadrivalent vaccine	Girls-only	51%	15·1 (12·8-16·8)	14·3 (12·1-16·1)	12·6 (10·7-14·5)	19·4 (15·2-25·1)	18·3 (14·2-24·0)	15·3 (11·4-20·6)

		90%	14·2 (12·3-16·0)	13·0 (11·3-14·8)	11·3 (9·9-12·8)	18·6 (14·5-24·3)	16·4 (12·5-22·2)	12·34 (8·9-17·2)
		G51% & B16%	14·9 (12·7-16·7)	14·0 (11·9-15·8)	12·2 (10·4-14·1)	19·1 (14·9-24·8)	17·7 (13·7-23·4)	14·2 (10·5-19·5)
	Gender-neutral	51%	14·6 (12·5-16·4)	13·5 (11·5-15·3)	11·5 (10·0-13·0)	18·6 (14·5-24·3)	16·7 (12·8-22·5)	12·7 (9·2-17·7)
		90%	14·0 (12·2-15·8)	12·9 (11·3-14·7)	11·3 (9·9-12·8)	18·0 (13·9-23·6)	15·9 (12·0-21·4)	12·3 (8·9-17·1)

HPV: human papillomavirus, G: Girls, B: Boys, NI: not included in the vaccine. Note: The values correspond to the mean (range) of the 50 simulations.

Table S11. Relative reduction in age-standardized carcinogenic HPV incidence rates (per 100 person-years) by HPV type group, HPV vaccine, vaccination strategy, and vaccination coverage. The values correspond to the mean (range) of the 50 estimates from the parameter sets.

	Vaccination strategy	Vaccination coverage	Female Year 2030	Female Year 2040	Female Year 2100	Male Year 2030	Male Year 2040	Male Year 2100
Nonavalent vs No vaccination	Girls-only	51%	0·74 (0·69-0·78)	0·64 (0·57-0·69)	0·53 (0·46-0·61)	0·82 (0·78-0·88)	0·71 (0·62-0·79)	0·54 (0·42-0·70)
		90%	0·64 (0·58-0·69)	0·50 (0·43-0·59)	0·44 (0·37-0·57)	0·74 (0·67-0·82)	0·55 (0·45-0·65)	0·37 (0·28-0·52)
	Gender-neutral	G51% & B16%	0·72 (0·66-0·76)	0·60 (0·53-0·66)	0·50 (0·42-0·59)	0·79 (0·74-0·85)	0·65 (0·56-0·74)	0·47 (0·34-0·64)
		51%	0·68 (0·62-0·73)	0·55 (0·47-0·61)	0·45 (0·40-0·57)	0·74 (0·67-0·81)	0·57 (0·47-0·65)	0·39 (0·31-0·52)
		90%	0·60 (0·53-0·66)	0·49 (0·42-0·59)	0·44 (0·37-0·57)	0·67 (0·59-0·75)	0·51 (0·42-0·59)	0·37 (0·28-0·52)
Quadrivalent vs No vaccination	Girls-only	51%	0·84 (0·80-0·86)	0·81 (0·77-0·84)	0·77 (0·71-0·81)	0·88 (0·83-0·92)	0·85 (0·77-0·90)	0·77 (0·66-0·86)
		90%	0·79 (0·75-0·83)	0·74 (0·68-0·79)	0·69 (0·61-0·75)	0·85 (0·80-0·89)	0·76 (0·70-0·82)	0·62 (0·52-0·71)
	Gender-neutral	G51% & B16%	0·83 (0·79-0·86)	0·80 (0·75-0·83)	0·74 (0·69-0·78)	0·87 (0·82-0·91)	0·82 (0·74-0·87)	0·72 (0·60-0·81)
		51%	0·81 (0·78-0·84)	0·77 (0·73-0·80)	0·70 (0·64-0·75)	0·85 (0·80-0·89)	0·78 (0·70-0·83)	0·64 (0·55-0·71)
		90%	0·78 (0·74-0·82)	0·73 (0·67-0·79)	0·69 (0·61-0·75)	0·82 (0·78-0·86)	0·74 (0·69-0·80)	0·62 (0·51-0·71)
Nonavalent vs Quadrivalent	Girls-only	51%	0·89 (0·85-0·92)	0·79 (0·72-0·85)	0·70 (0·63-0·78)	0·93 (0·90-0·96)	0·83 (0·75-0·90)	0·70 (0·61-0·81)
		90%	0·81 (0·74-0·86)	0·68 (0·60-0·75)	0·64 (0·58-0·76)	0·88 (0·82-0·93)	0·72 (0·62-0·82)	0·60 (0·48-0·74)
	Gender-neutral	G51% & B16%	0·87 (0·83-0·91)	0·76 (0·69-0·83)	0·67 (0·60-0·77)	0·91 (0·87-0·95)	0·79 (0·70-0·87)	0·66 (0·55-0·79)
		51%	0·84 (0·78-0·89)	0·71 (0·63-0·79)	0·65 (0·58-0·76)	0·87 (0·81-0·92)	0·73 (0·63-0·83)	0·61 (0·48-0·75)
		90%	0·77 (0·69-0·84)	0·67 (0·59-0·74)	0·64 (0·58-0·76)	0·81 (0·74-0·88)	0·68 (0·59-0·79)	0·59 (0·47-0·74)
Nonavalent	Girls-only	90% vs 51%	0·86 (0·81-0·90)	0·79 (0·73-0·88)	0·82 (0·70-0·94)	0·90 (0·86-0·93)	0·78 (0·72-0·86)	0·69 (0·49-0·91)
	Gender-neutral	90% vs G51% & B16%	0·83 (0·77-0·89)	0·81 (0·74-0·91)	0·88 (0·74-0·98)	0·84 (0·80-0·90)	0·78 (0·71-0·89)	0·79 (0·54-0·97)
		90% vs 51%	0·88 (0·83-0·93)	0·89 (0·83-0·96)	0·97 (0·85-1·00)	0·91 (0·88-0·94)	0·89 (0·85-0·96)	0·94 (0·76-1·00)
Quadrivalent	Girls-only	90% vs 51%	0·94 (0·91-0·97)	0·91 (0·85-0·95)	0·90 (0·79-0·97)	0·96 (0·93-0·97)	0·90 (0·84-0·94)	0·81 (0·63-0·94)
	Gender-neutral	90% vs G51% & B16%	0·96 (0·93-0·98)	0·95 (0·90-0·98)	0·98 (0·89-1·00)	0·97 (0·95-0·98)	0·95 (0·91-0·99)	0·97 (0·84-1·00)
		90% vs 51%	0·96 (0·93-0·98)	0·95 (0·90-0·98)	0·98 (0·89-1·00)	0·97 (0·95-0·98)	0·95 (0·91-0·99)	0·97 (0·84-1·00)

HPV: human papillomavirus, G: Girls, B: Boys, NI: not included in the vaccine. Note: The values correspond to the mean (range) of the 50 simulations.

Breakthrough infections and herd immunity

Table S12. Age-standardized carcinogenic HPV prevalence among individuals aged 15 years and older by HPV type, vaccination strategy, vaccination coverage, and vaccination status. The values correspond to the mean (range) of the 50 estimates from the parameter sets.

	Vaccination strategy	Vaccination coverage	Vaccinated Year 2030	Vaccinated Year 2040	Vaccinated Year 2100	Unvaccinated Year 2030	Unvaccinated Year 2040	Unvaccinated Year 2100
HPV 16/18	Girls-only	51%	0·09% (0·05-0·15)	0·10% (0·05-0·17)	0·08% (0·02-0·17)	4·72% (2·96-6·19)	4·20% (2·15-5·99)	2·81% (0·68-5·09)
		90%	0·07% (0·04-0·11)	0·04% (0·02-0·07)	<0·01% (<0·01%-0·01)	4·06% (2·48-5·57)	2·46% (1·10-3·84)	0·09% (<0·01-0·28)
	Gender-neutral	G51% & B16%	0·12% (0·07-0·20)	0·13% (0·06-0·25)	0·12% (0·01-0·31)	4·58% (2·84-6·06)	3·88% (1·86-5·63)	2·05% (0·22-4·50)
		51%	0·12% (0·06-0·20)	0·12% (0·05-0·25)	0·06% (<0·01-0·26)	4·41% (2·70-5·90)	3·23% (1·42-4·94)	0·64% (0·02-2·53)
		90%	0·08% (0·04-0·11)	0·05% (0·02-0·09)	<0·01% (<0·01%-0·01)	3·92% (2·38-5·46)	2·32% (1·04-3·70)	0·04% (<0·01-0·11)
HPV 31/33/45/52/58	Girls-only	51%	0·09% (0·07-0·11)	0·10% (0·06-0·14)	0·02% (<0·01-0·08)	5·11% (3·83-6·99)	2·92% (1·51-4·71)	0·48% (0·01-1·50)
		90%	0·07% (0·05-0·08)	0·04% (0·01-0·08)	<0·01% (<0·01%-0·01)	4·37% (3·13-6·30)	1·43% (0·28-3·23)	0·01% (<0·01%-0·07)
	Gender-neutral	G51% & B16%	0·08% (0·06-0·09)	0·08% (0·04-0·12)	0·01% (<0·01-0·04)	4·96% (3·67-6·87)	2·55% (1·15-4·41)	0·17% (<0·01-0·86)
		51%	0·07% (0·05-0·09)	0·04% (0·01-0·08)	<0·01% (<0·01%-0·01)	4·63% (3·32-6·59)	1·85% (0·50-3·77)	0·02% (<0·01-0·22)
		90%	0·04% (0·03-0·06)	0·01% (<0·01-0·04)	<0·01% (<0·01%-<0·01)	3·91% (2·65-5·92)	1·18% (0·16-2·90)	<0·01% (<0·01%-0·03)
HPV 16/18/31/33/45/52/58	Girls-only	51%	0·17% (0·12-0·25)	0·19% (0·12-0·25)	0·10% (0·03-0·19)	9·42% (7·38-12·62)	6·88% (4·60-9·95)	3·25% (0·99-5·66)
		90%	0·14% (0·10-0·19)	0·07% (0·04-0·13)	<0·01% (<0·01%-0·01)	8·08% (6·30-11·24)	3·78% (2·24-6·23)	0·09% (0·01-0·29)
	Gender-neutral	G51% & B16%	0·19% (0·12-0·28)	0·20% (0·11-0·31)	0·13% (0·02-0·31)	9·15% (7·12-12·39)	6·22% (3·95-9·27)	2·21% (0·38-4·54)
		51%	0·18% (0·11-0·27)	0·16% (·08-0·28)	0·06% (<0·01-0·26)	8·67% (6·68-11·97)	4·93% (2·85-7·84)	0·66% (0·03-2·53)
		90%	0·12% (0·07-0·16)	0·06% (0·03-0·11)	<0·01% (<0·01-0·01)	7·52% (5·77-10·79)	3·40% (2·01-5·68)	0·04% (<0·01-0·12)

HPV: human papillomavirus, G: Girls, B: Boys. Note: HPVs 16/18 refers to infection with HPV16 and/or 18. And HPVs 31/33/45/52/58 refers to infection with any of HPV types 31, 33, 45, 52, and 58.

Age-standardized cervical cancer incidence

Table S13. Projections on cervical cancer incidence based on carcinogenic HPV prevalence. The values correspond to the mean (range) of 50 simulations. Age- standardization was performed using 2015 World Female Population.

HPV vaccination and coverage scenarios	Year for reaching threshold in in cervical cancer incidence (range)	Expected age-standardized incidence rate of cervical cancer cases per 100,000 females by 2100 (range)
**Girls-only vaccination**		
Nonavalent 51% coverage	2082 (2068-2100)	3·77 (2·08-5·08)
Nonavalent 90% coverage	2060 (2050-2066)	0·87 (0·81-0·95)
Quadrivalent 51% coverage	Not achievable	7·34 (5·64-8·81)
Quadrivalent 90% coverage	Not achievable	4·69 (4·63-4·77)
Gender-neutral vaccination		
Nonavalent G51% B16% coverage	2076 (2062-2099)	2·81 (1·39-4·34)
Nonavalent 51% coverage	2064 (2054-2072)	1·43 (0·89-2·64)
Nonavalent 90% coverage	2058 (2048-2064)	0·83 (0·80-0·86)
Quadrivalent G51% B16% coverage	Not achievable	6·53 (5·01-8·16)
Quadrivalent 51% coverage	Not achievable	5·23 (4·68-6·46)
Quadrivalent 90% coverage	Not achievable	4·65 (4·62-4·68)

Table S14. Projections on cervical cancer incidence based on carcinogenic HPV prevalence. The values correspond to the mean (range) of 50 simulations. Age- standardization was performed using 1996 Segi Segi/Doll standard population.

HPV vaccination and coverage scenarios	Year for reaching threshold in in cervical cancer incidence (range)	Expected age-standardized incidence rate of cervical cancer cases per 100,000 females by 2100 (range)
**Girls-only vaccination**		
Nonavalent 51% coverage	2076 (2062-2097)	3·26 (1·79-4·40)
Nonavalent 90% coverage	2056 (2048-2061)	0·74 (0·70-0·80)
Quadrivalent 51% coverage	Not achievable	6·35 (4·88-7·63)
Quadrivalent 90% coverage	Not achievable	4·04 (4·00-4·10)
Gender-neutral vaccination		
Nonavalent G51% B16% coverage	2068 (2058-2080)	2·42 (1·19-3·77)
Nonavalent 51% coverage	2060 (2050-2066)	1·23 (0·76-2·28)
Nonavalent 90% coverage	2054 (2045-2061)	0·71 (0·69-0·73)
Quadrivalent G51% B16% coverage	Not achievable	5·64 (4·33-7·07)
Quadrivalent 51% coverage	Not achievable	4·52 (4·04-5·59)
Quadrivalent 90% coverage	2098 (2096-2100)	4·01 (4·00-4·03)

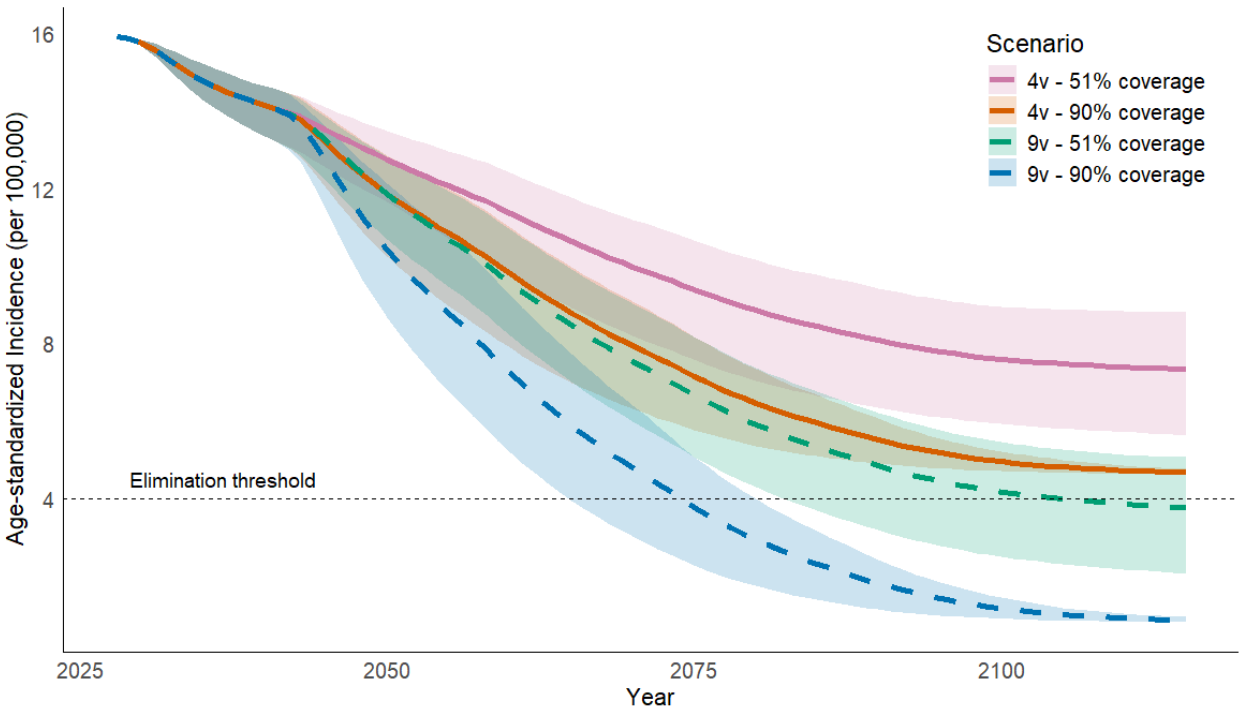

Figure S22. Estimated age-standardized cervical cancer incidence projections in girls-only vaccination scenarios.
Note: The thick lines represent the mean and the shaded areas indicate the range of 50 simulations. Age-standardization was performed using the 2015 World Female Population. [3]

Table S15. Sensitivity analysis considering waning vaccine-immunity in cervical cancer incidence projections. The values correspond to the mean (range) of 50 simulations. Age- standardization was performed using 2015 World Female Population. [3]

HPV vaccination and coverage scenarios	Year for reaching threshold in in cervical cancer incidence (range)	Expected age-standardized incidence rate of cervical cancer cases per 100,000 females by 2100 (range)
Gender-neutral vaccination		
Nonavalent 51% coverage	2072 (2057-2093)	2·16 (0·99-4·63)
Nonavalent 90% coverage	2061 (2049-2070)	0·96 (0·83-1·15)

Note: For this analysis, we assumed that vaccine-induced immunity fully wanes 20 years after vaccination.

REFERENCES

1. Malagon T, Lemieux-Mellouki P, Laprise JF, Brisson M. Bias due to correlation between times-at-risk for infection in epidemiologic studies measuring biological interactions between sexually transmitted infections: A case study using human papillomavirus type interactions. *Am J Epidemiol* 2016; **184**(12): 873-83.
2. Karline S, Thomas P, Woodrow SR. Solving Differential Equations in R: Package deSolve. *J Stat Softw* 2010; **33**(9): 1-25.
3. Population Division. Department of Economic and Social Affairs. World Population Prospects 2024. Online Edition. 2024. https://population.un.org/wpp/downloads (accessed 30/01/2024).
4. Population Division. Department of Economic and Social Affairs. Standard Projections (Estimates and Projections scenarios). 2022. https://population.un.org/wpp/Download/ (accessed Mar 24 2023).
5. Burchell AN, Tellier PP, Hanley J, Coutlee F, Franco EL. Human papillomavirus infections among couples in new sexual relationships. *Epidemiology* 2010; **21**(1): 31-7.
6. Walker R, Nickson C, Lew JB, Smith M, Canfell K. A revision of sexual mixing matrices in models of sexually transmitted infection. *Stat Med* 2012; **31**(27): 3419-32.
7. Garnett GP, Anderson RM. Balancing sexual partnerships in an age and activity stratified model of HIV transmission in heterosexual populations. *IMA J Math Appl Med Biol* 1994; **11**(3): 161-92.
8. World Health Organization. Human papillomavirus vaccines: WHO position paper (2022 update). *Weekly epidemiological record* 2022; **50**(97): 645-72.
9. Bogaards JA, Xiridou M, Coupe VM, Meijer CJ, Wallinga J, Berkhof J. Model-based estimation of viral transmissibility and infection-induced resistance from the age-dependent prevalence of infection for 14 high-risk types of human papillomavirus. *Am J Epidemiol* 2010; **171**(7): 817-25.
10. Insinga RP, Perez G, Wheeler CM, et al. Incidence, duration, and reappearance of type-specific cervical human papillomavirus infections in young women. *Cancer Epidemiol Biomarkers Prev* 2010; **19**(6): 1585-94.
11. Giuliano AR, Lee J-H, Fulp W, et al. Incidence and clearance of genital human papillomavirus infection in men (HIM): a cohort study. *Lancet* 2011; **377**(9769): 932-40.
12. Carter JJ, Koutsky LA, Hughes JP, et al. Comparison of human papillomavirus types 16, 18, and 6 capsid antibody responses following incident infection. *J Infect Dis* 2000; **181**(6): 1911-9.
13. Tota JE, Giuliano AR, Goldstone SE, et al. Anogenital human papillomavirus (HPV) infection, seroprevalence, and risk factors for HPV seropositivity among sexually active men enrolled in a global HPV vaccine trial. *Clin Infect Dis* 2022; **74**(7): 1247-56.
14. Brown DR, Castellsague X, Ferris D, et al. Human papillomavirus seroprevalence and seroconversion following baseline detection of nine human papillomavirus types in young women. *Tumour Virus Res* 2022; **13**: 200236.
15. Artemchuk H, Triglav T, Ostrbenk A, Poljak M, Dillner J, Faust H. Seroprevalences of Antibodies to 11 Human Papillomavirus (HPV) Types Mark Cumulative HPV Exposure. *J Infect Dis* 2018; **218**(3): 398-405.
16. Edelstein ZR, Carter JJ, Garg R, et al. Serum antibody response following genital alpha9 human papillomavirus infection in young men. *J Infect Dis* 2011; **204**(2): 209-16.
17. Malagon T, Trottier H, El-Zein M, Villa LL, Franco EL, Ludwig-McGill Cohort S. Human papillomavirus intermittence and risk factors associated with first detections and redetections in the Ludwig-McGill cohort study of adult women. *J Infect Dis* 2023; **228**(4): 402-11.
18. National Center for Health Statistics. Centers for Disease Control and Prevention. National Survey of Family Growth Homepage. 2023. https://www.cdc.gov/nchs/nsfg/index.htm (accessed Mar 31 2023).
19. Malagon T, Burchell A, El-Zein M, et al. Assortativity and mixing by sexual behaviors and sociodemographic characteristics in young adult heterosexual dating partnerships. *Sex Transm Dis* 2017; **44**(6): 329-37.
20. Garnett GP, Hughes JP, Anderson RM, et al. Sexual mixing patterns of patients attending sexually transmitted diseases clinics. *Sex Transm Dis* 1996; **23**(3): 248-57.
21. Waldrop-Valverde DG, Davis TL, Sales JM, Rose ES, Wingood GM, DiClemente RJ. Sexual concurrency among young African American women. *Psychol Health Med* 2013; **18**(6): 676-86.
22. Wang SS, Schiffman M, Herrero R, et al. Determinants of human papillomavirus 16 serological conversion and persistence in a population-based cohort of 10 000 women in Costa Rica. *Br J Cancer* 2004; **91**(7): 1269-74.

23. FUTURE II Study Group. Quadrivalent vaccine against human papillomavirus to prevent high-grade cervical lesions. *N Engl J Med* 2007; **356**(19): 1915-27.
24. Joura EA, Giuliano AR, Iversen O-E, et al. A 9-valent HPV vaccine against infection and intraepithelial neoplasia in women. *N Engl J Med* 2015; **372**(8): 711-23.
25. Giuliano AR, Joura EA, Garland SM, et al. Nine-valent HPV vaccine efficacy against related diseases and definitive therapy: comparison with historic placebo population. *Gynecol Oncol* 2019; **154**(1): 110-7.
26. Owusu-Edusei KP, C.; Ovcinnikova, O.; Favato, G.; Daniels, V. Assessing the Health and Economic Outcomes of a 9-Valent HPV Vaccination Program in the United Kingdom. *J Health Econ Outcomes Res* 2022; **9**(1): 140-50.
27. Wellings K, Collumbien M, Slaymaker E, et al. Sexual behaviour in context: a global perspective. *Lancet* 2006; **368**(9548): 1706-28.
28. Ministerio de Salud y Desarrollo Social. Encuesta Mundial de Salud Escolar 2018. Resumen ejecutivo. Buenos Aires, Argentina: Ministerio de Salud y Desarrollo Social; 2018. p. 13.
29. Newman MEJ. Mixing patterns in networks. *Physical Review*; **E2003**.
30. Molano M, Posso H, Weiderpass E, et al. Prevalence and determinants of HPV infection among Colombian women with normal cytology. *Br J Cancer* 2002; **87**(3): 324-33.
31. Del Rio-Ospina L, Soto-De Leon SC, Camargo M, et al. The Prevalence of High-Risk HPV Types and Factors Determining Infection in Female Colombian Adolescents. *PLoS One* 2016; **11**(11): e0166502.
32. Puerto D, Reyes V, Lozano C, et al. Detection and Genotyping of HPV DNA in a Group of Unvaccinated Young Women from Colombia: Baseline Measures Prior to Future Monitoring Program. *Cancer Prev Res* 2018; **11**(9): 581-92.
33. Leon S, Sanchez R, Patarroyo MA, et al. Prevalence of HPV-DNA and anti-HPV antibodies in women from Girardot, Colombia. *Sex Transm Dis* 2009; **36**(5): 290-6.
34. Munoz N, Kato I, Bosch FX, et al. Risk factors for HPV DNA detection in middle-aged women. *Sex Transm Dis* 1996; **23**(6): 504-10.
35. Velicer C, Zhu X, Vuocolo S, Liaw KL, Saah A. Prevalence and incidence of HPV genital infection in women. *Sex Transm Dis* 2009; **36**(11): 696-703.
36. Correa RM, Baena A, Valls J, et al. Distribution of human papillomavirus genotypes by severity of cervical lesions in HPV screened positive women from the ESTAMPA study in Latin America. *PLoS One* 2022; **17**(7): e0272205.
37. Baena A, Maribel A. Prevalencia de VPH en estudio ESTAMPA. Personal comunication.
38. Castellsague X, Ghaffari A, Daniel RW, Bosch FX, Munoz N, Shah KV. Prevalence of penile human papillomavirus DNA in husbands of women with and without cervical neoplasia: a study in Spain and Colombia. *J Infect Dis* 1997; **176**(2): 353-61.
39. Sudenga SL, Torres BN, Silva R, et al. Comparison of the natural history of genital HPV infection among men by bountry: Brazil, Mexico, and the United States. *Cancer Epidemiol Biomarkers Prev* 2017; **26**(7): 1043-52.
40. Carnell R. lhs: Latin Hypercube Samples. 2024.
41. Ferlay J, Ervik M, Lam F, et al. Global Cancer Observatory: Cancer Today. 2024. https://gco.iarc.who.int/today (accessed 04/020/2025).
42. Ervik M, Lam F, Laversanne M, et al. Global Cancer Observatory: Cancer Over Time. 2024. https://gco.iarc.who.int/overtime/en/about (accessed 09/10/2025).
43. Kreimer AR, Herrero R, Sampson JN, et al. Evidence for single-dose protection by the bivalent HPV vaccine-Review of the Costa Rica HPV vaccine trial and future research studies. *Vaccine* 2018; **36**(32 Pt A): 4774-82.
