# Supplementary material for: Impact of switching from a quadrivalent to a nonavalent HPV vaccine on HPV infections and cervical cancer in Colombia: a mathematical modelling study
Source: Lancet Reg Health Am. 2026 May 5;58:101483. doi: 10.1016/j.lana.2026.101483 (PMC13158420; doi:10.1016/j.lana.2026.101483)
Supplement: Supplementary Material [file mmc2.docx]

**RESUMEN**

**Introducción**: En 2022, Colombia reportó 13·7 casos de cáncer de cuello uterino por 100,000 mujeres. Dado el persistente bajo nivel de cobertura de vacunación contra el virus del papiloma humano (VPH) en niñas (51%), se están considerando intervenciones alternativas. Nuestro objetivo fue evaluar el impacto a nivel poblacional de cambiar de una vacuna tetravalente a una vacuna nonavalente, así como de aumentar los niveles de cobertura al objetivo de la Organización Mundial de la Salud de 90% (≥1 dosis).

**Métodos:** Desarrollamos un modelo dinámico de la transmisión y vacunación del VPH carcinogénico en la población colombiana de15 años a más, estratificada según su estado de salud, sexo, edad, nivel de actividad sexual y estado de vacunación contra el VPH, considerando la latencia de VPH. Evaluamos la vacunación rutinaria de niños y niñas, así como la de solo niñas (<15 años), con una dosis, bajo los niveles de cobertura actuales en Colombia y 90%. El modelo fue calibrado con datos de prevalencia de VPH de Colombia y América Latina. Calculamos la prevalencia e incidencia estandarizadas por edad del VPH, así como la reducción relativa en la prevalencia, y en la incidencia de cáncer de cuello uterino en un periodo de 88 años.

**Hallazgos:** Ambas vacunas redujeron la prevalencia estandarizada por edad del VPH, observándose mayores reducciones con niveles de cobertura de 90% y con la vacuna nonavalente. Cambiar a una vacuna nonavalente con la cobertura actual reduciría la prevalencia de VPH en mujeres al año 2100 en 39% (rango: 33%-46%), comparado con 8% (rango: 1%-17%) cuando solo se incrementa la cobertura de una vacuna tetravalente a 90% en un escenario de vacunación de niños y niñas. Únicamente una vacuna nonavalente redujo la incidencia estandarizada por edad de cáncer de cuello uterino proyectada por debajo de 4 casos por 100,000 mujeres tan temprano como 2058.

**Interpretación:** El cambio a una vacuna nonavalente acelerará la reducción de las infecciones por VPH, agilizando así el progreso hacia la eliminación del cáncer cervicouterino.
